# Control of Gemcitabine Activity With Blue and Red Light

**DOI:** 10.1002/chem.71363

**Published:** 2026-07-06

**Authors:** Nils F. Kersten, Konstantin L. Stock, Marius Hyprath, Hannah Weisser, Timo Weinrich, Dieter Steinhilber, Alexander Heckel

**Affiliations:** ^1^ Institute for Organic Chemistry and Chemical Biology Goethe‐University Frankfurt am Main Germany; ^2^ Department of Chemistry, and FRQNT Centre for Green Chemistry and Catalysis McGill University Montreal Quebec Canada; ^3^ Institute of Pharmaceutical Chemistry Goethe‐University Frankfurt am Main Germany

**Keywords:** cytostatics, cytotoxicity, photoactivated chemotherapy, photochemistry, photoremovable protecting groups

## Abstract

Treating cancer with chemotherapy is generally associated with many side effects. This problem can be addressed by selectively releasing the desired drugs with light. In a screening approach, we modified known antimetabolite cytostatics with photoremovable protecting groups (PPGs). Sixteen new light‐activatable cytostatics with a blue light‐activatable diethylaminocoumarin group (DEACM) were synthesized, and their (photo‐)chemical properties were tested. The most promising compounds were subjected to cytotoxicity studies with HT‐29 colon cancer cells. In our experiments, the DEACM‐gemcitabine derivative showed the best properties with full recovery of gemcitabine activity after irradiation. In the absence of blue light, this compound was 29 times less toxic. In the next step, based on these results, we designed a red‐light activatable derivative of gemcitabine, featuring a heptamethine (Cy7) PPG. We were able to confirm the photorelease of gemcitabine with tissue‐penetrating red light in HT‐29 cells. Our “gem‐Cy‐tabine” was almost inactive in the absence of light, but its activity increased >33 fold after irradiation with red light. It is, therefore, an interesting candidate for more specific cancer therapy by photopharmacology.

## Graphical abstract

Treating cancer with chemotherapy is generally associated with many side effects, which can be addressed by selectively releasing the desired drugs with visible light. Sixteen new blue light‐activatable cytostatics were introduced, with the gemcitabine derivative exhibiting ideal properties. Subsequently, we designed the red‐light activatable derivative “gem‐Cy‐tabine”, allowing us to control cancer activity in the “phototherapeutic window”.

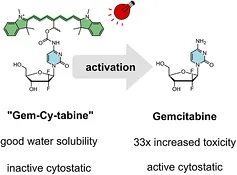

## 1 Introduction

Cancer is one of the leading causes of death in most industrialized countries, with steadily increasing incidence and mortality [1, 2]. The challenge of selective therapeutic targeting of cancer cells remains one of the major focus points of modern drug development. The diversity of different cell types that can be affected by cancer and the immense number of possible mutations that can occur make treatment even more complicated. To date, several treatment options are available. The traditionally well‐established therapeutic options are surgery, radiotherapy, and chemotherapy, which are widely used [3, 4, 5, 6]. In addition, modern therapeutic approaches like immunotherapy, targeted tumor therapy, and gene therapy play an increasingly important role in cancer treatment [3, 4]. Although surgery and radiotherapy can be highly effective, limitations can be the location and state of the disease, which makes these treatment options unsuitable. In chemotherapy, nucleic acids can be targeted with alkylating agents [7] or platinum complexes, which lead to cross‐linked DNA strands [8, 9]. Another approach uses antimetabolites, which comprise two main classes: nucleotide analogues and antifolates. These drugs mimic their native counterparts and interfere with the *de novo* DNA synthesis by inhibiting key enzymes of ribonucleotide metabolism [10, 11, 12]. Another therapeutic approach in oncology is immunotherapy [3, 4, 13]. Although this relatively new treatment modality provides a highly valuable option, chemotherapeutics remain an important backbone of treatment guidelines for most solid tumors [4, 14]. However, a significant problem of all chemotherapeutic approaches is that not only malignant cells but also healthy cells are affected, leading to severe side effects of chemotherapy [15, 16, 17, 18, 19].

Modern approaches aim to combine the advantages of high effectiveness with a decreased severity of side effects, but often come with the cost of high expenses, limited availability, and the need for extensive personalized diagnostics. A promising approach for cancer therapy is therefore the combination of highly effective chemotherapeutics and innovative approaches to directly or selectively target the cancer cells. Targeted cancer therapy approaches can include more selective agents such as antibody‐drug conjugates (ADCs) [20], nanocarriers [21, 22], or light [23, 24, 25, 26].

Light was first used in cancer treatment decades ago in photodynamic therapy. In this strategy, reactive oxygen species (ROS) are released as singlet oxygen (type‐II) or as radical species (type‐I) after local excitation of so‐called photosensitizers [27, 28, 29, 30, 31]. Although light offers excellent spatiotemporal control, a major limitation of type‐II photodynamic therapy is its dependence on molecular oxygen for the photochemical reaction [32, 33, 34]. Tumor tissue, however, is frequently hypoxic [35, 36, 37, 38, 39]. Thus, newer studies focus on oxygen‐independent (type‐I) photosensitizers based on, e.g., nanomaterials [40, 41, 42], organic compounds [43, 44, 45, 46], or metal complexes [47, 48, 49, 50]. The benefits and limitations of these photosensitizers were reviewed extensively already elsewhere [51, 52, 53].

Photosensitizers are not the only way to control biological function with light. Trauner and Feringa coined the term “photopharmacology” [54, 55, 56] for the implementation of photoswitches into biologically active compounds to gain control over their pharmacological properties with light. In a seminal contribution, Trauner demonstrated how photoswitchable “azo‐propofol” could be used to reversibly sedate living tadpoles with light of two different wavelengths [57]. In the domain of cytostatics, methotrexate has been made light‐controllable by this “azalogisation strategy”—the engineering of an azobenzene photoswitch into the drug [58, 59].

An alternative to reversible photoswitching is irreversible “photocaging”. While the former sounds more versatile due to the formal choice of controlling ON and OFF with two different wavelengths [60], this strategy can be very difficult to implement. If several ON/OFF cycles are unnecessary, photocaging is more straightforward [61, 62] and usually leads to much cleaner ON and OFF states. Photocaging uses photoremovable protecting groups (PPGs), which block the biological activity of the desired payload [63]. After excitation of the photoremovable moiety with light of a suitable wavelength, the unmodified payload can be released due to a defined bond scission taking place upon irradiation [64, 65, 66]. The irradiation wavelength is determined by the properties of the applied PPG. While UV A‐activatable PPGs like nitroaryl groups or blue light activatable PPGs like coumarin derivatives have long been known, red‐light activatable alternatives are generally desired [66]. Red light can penetrate deeper into tissues [67, 68]. The term “phototherapeutic window” (650–900 nm) [69, 70, 71] has been coined. Consequently, considerable efforts have been made to establish red‐light activatable PPGs in the last decade. These new PPGs are based on coumarin [72], porphyrin‐ [73], BODIPY‐ [74, 75, 76], heptamethine‐ [77, 78, 79, 80, 81, 82, 83], and xanthene‐scaffolds [84, 85] and were utilized to release a variety of payloads.

Using photocages to release cytostatics is an emerging strategy, generally described as photoactivated chemotherapy [23, 24, 86, 87] (PACT). PACT could prove to be complementary to type‐I and especially type‐II PDT since PPGs usually release payloads in an oxygen‐independent manner. Additionally, due to the different underlying mechanisms of PACT, advantages and limitations are mainly determined by the intrinsic characteristics of the cytostatic payload.

Although PPGs exist with a wide range of release wavelengths as described previously, existing light‐activatable cytostatics still mostly rely on UV light‐activatable PPGs. Most PACT strategies are based on alkylating agents (chlorambucil [88], phosphoramide mustards [89], duocarmycin [90]), taxanes (paclitaxel) [91], anthracyclines (doxorubicine [91]), kinaseinhibitors (vemurafenib [92], imatinib [93]), and antimetabolites (fluoropyrimidines [94, 95, 96] and methotrexate [97]), studies for most of the nucleoside analogues are missing [23, 87]. In this study, we addressed this issue and further investigated the light control of nucleoside analogues. First, we screened the viability of different photorelease strategies of antimetabolites with a more established blue light‐activatable PPG. Based on the findings for this first set of compounds, we designed a red‐light activatable derivative to control cancer activity with red light.

## 2 Results and Discussion

### 2.1 Chemical Syntheses

Diethylaminocoumarin (DEACM) was chosen as a blue light–activatable PPG because it can be synthesized on a multigram scale and has already been widely applied in various photochemical and biological contexts. Two groups of antimetabolite cytostatics were considered in this work, the antifolates pemetrexed (**PMX**), raltitrexed (**RTX**), and methotrexate (**MTX**). The nucleoside analogue antimetabolites 6‐mercaptopurin (**6MP**), gemcitabine (**Gem**), clofarabine (**Clf**), cladribine (**Cla**), fludarabine (**araF**), decytabine (**Dec**), cytarabine (**araC**), 5‐fluorouracil (**5FU**), tegafur (**Teg**), and floxuridine (**Flox**) were also chosen as potential targets for light‐activatable anti‐cancer drugs. Figure 1 gives an overview of the synthesized photocaged derivatives with different caging strategies.

**FIGURE 1:**
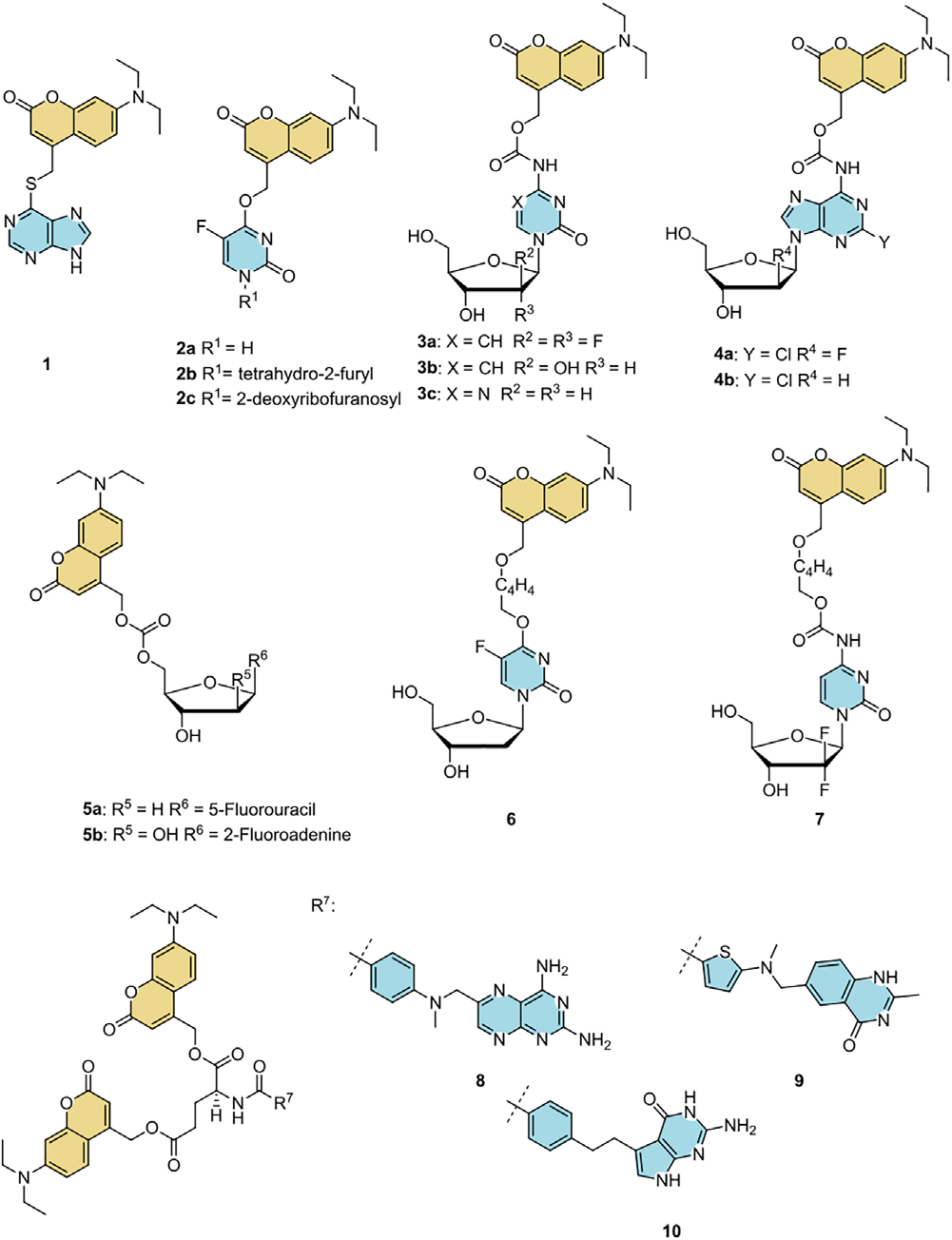
Overview of coumarin‐substituted antimetabolite cytostatics synthesized in this work. The photolabile DEACM group is highlighted in yellow.

Coumarin alcohol (**DEACM‐OH**, highlighted in yellow) was used as the main starting material for the synthesis of light‐activatable cytostatics and prepared according to Weinrich et al. [98]. Introduction of **DEACM‐OH** (Figures S31–S34) afforded thioether **1**, iminols **2a**–**c**, and carbamates **3a**–**c** and **4a**–**b**.

Following similar synthetic approaches, quinone methide‐based self‐immolative linkers were installed affording compounds **6** and **7** using the corresponding coumarin benzylalcohol (**DEACM‐Bn‐OH**, Figures S32 and S33) [99]. The implementation of this linker is a known strategy to enhance uncaging of nucleobase payloads as demonstrated by Choi et al. [99].

Photocaging of the sugar‐residue was explored by attaching the coumarin group via a carbonate linker to the 5′‐postion of floxuridine (Figure S32)–yielding compound **5a**. In the case of **araF** derivative **5b**, the same linker strategy was applied, although in a different synthetic approach (Figure S35). A carbonate linker was used in these cases because the direct attachment of the coumarin group as an ether could not be achieved.

The antifolate cytostatics **MTX**, **RTX,** and **PMX** were also considered as possible candidates for this study. They possess a glutamate residue, which was doubly substituted with the alcohol **DEACM‐OH** (compounds **8–10**, Figures 1 and S36). Attachment of the coumarin group to other residues of the antifolates was extensively investigated, but did not yield the desired results.

Thus, a total of 16 compounds (Figure 1) bearing blue light‐absorbing DEACM‐moieties were synthesized. In the next step, they were screened for selected (photo)chemical properties.

### 2.2 Characterization of Coumarin‐Substituted Cytostatics

The synthesized compounds were investigated in aqueous solutions (0.1 x PBS) containing 5% DMSO as a cosolvent (Table 1). Compounds **8**–**10** exhibited limited solubility. Therefore 10% DMSO was used in these cases. All derivatives possess an absorption maximum around 396 nm with extinction coefficients ranging from 12,700 to 62,700 M^−1^cm^−1^. The corresponding absorption spectra are shown in Figure 2a. In the case of the decitabine derivative **3c**, no clear data was obtained due to an almost immediate decomposition in water. Hydrolysis of the azacytidine scaffold was reported before by Nguyen et al. [100]. Compounds **5a** and **5b** showed hydrolysis in PBS over 2 d (Figures S13 and S14), while compound **7** showed some hydrolysis over 12 d in PBS (Figure S15). The rest of the presented derivatives did not show any decomposition in aqueous solutions (PBS, 5% DMSO) over several days in the dark. For stability tests under cell culture conditions *vide infra*.

**TABLE 1:** Basic (photo‐)chemical properties of compounds **1**–**10**. Absorption spectra were measured in 0.1 x PBS containing 5%–50% DMSO as a cosolvent. Photolysis and stability were investigated in 0.1 x PBS (25–50 µM, 5%–10% DMSO, ambient temperature) or in cell culture medium (DMEM, in the presence of HT‐29 cells, 37°C). Irradiations were conducted at 455 nm with an in‐house built LED‐device (455 nm, 13 mW/cm^2^).

Compound	Payload	λ_max_/nm	ε_max_/M^−1 ^cm^−1^	Quantum yield Φ/%	Photolysis	Solubility	Stability (PBS, r.t.)	Stability (DMEM, 37°C)
				(PBS)	(DMEM)			
**1**	6MP	389	18900	n.d.^a^	×	>50 µM, (5% DMSO)	✓	✓
**2a**	5FU	389	25600	4.0	n.d.	>50 µM, (5% DMSO)	✓	n.d.
**2b**	Teg	394	18200	n.d.^b^	n.d.	<50 µM, (5% DMSO)	✓	n.d.
**2c**	Flox	389	24200	6.9	✓	>50 µM, (5% DMSO)	✓	✓
**3a**	Gem	394	20700	5.5	✓	>50 µM, (5% DMSO)	✓	✓
**3b**	araC	394	22700	4.5	✓	>50 µM, (5% DMSO)	✓	✓
**3c**	Dec	n.d.	n.d.	n.d.	n.d.	hydrolysis (1 h)	hydrolysis (1h)	n.d.
**4a**	Clf	394	17000	3.0	✓	>50 µM, (5% DMSO)	✓	✓
**4b**	Cla	389	18000	0.7	✓	>50 µM, (5% DMSO)	✓	✓
**5a**	Flox	394	12700	10.6	✓	>50 µM, (5% DMSO)	hydrolysis (2 d)	hydrolysis, (3 d)
**5b**	araF	398	14600	12.4	✓	>50 µM, (5% DMS.)	hydrolysis (2 d)	hydrolysis, (3 d)
**6**	Flox	398	13200	n.d.^a^	n.d.	>50 µM, (5% DMSO)	n.d.	n.d.
**7**	Gem	394	13800	n.d.^a^	n.d.	>50 µM, (5% DMSO)	hydrolysis (12 d)	n.d.
**8**	MTX	389	48000	n.d.^c^	n.d.	<25 µM, (10% DMSO)	✓	n.d.
**9**	RTX	394	62700	n.d.^c^	n.d.	<25 µM, (10% DMSO)	✓	n.d.
**10**	PMX	394	21400	n.d.^c^	n.d.	<25 µM, (10% DMSO)	✓	n.d.

^a^ Not determined due to incomplete photorelease.

^b^ Not determined due to low solubility.

^c^ Not determined due to decomposition.

**FIGURE 2:**
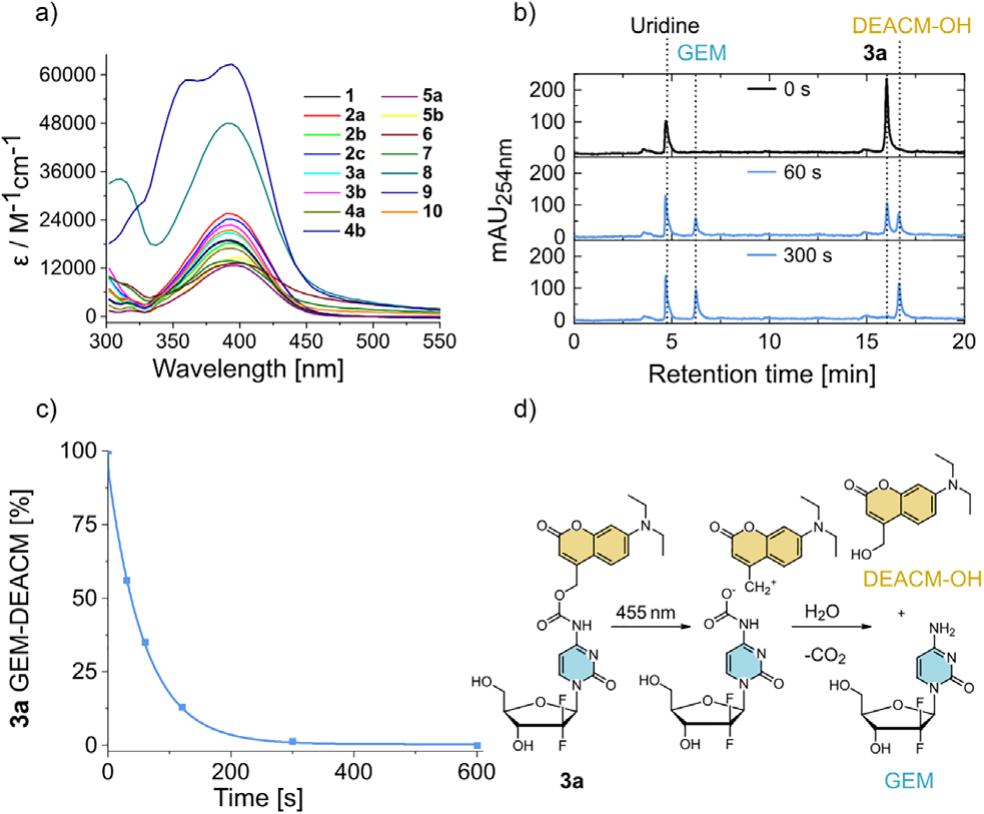
(a) Absorption spectra of coumarin‐substituted compounds **1–10** in PBS/DMSO (5%–50%). (b) HPLC‐traces of **3a** in PBS/DMSO (5%) before and after irradiation with blue LEDs (455 nm, 13 mW/cm^2^) in the presence of uridine as a standard. (c) Photolysis kinetic of **3a** after irradiation with blue LEDs (455 nm, 13 mW/cm^2^) at different time points. (d) Proposed reaction scheme of the photodeprotection reaction of **3a**.

Next, the photolytic behavior of the coumarin derivatives was investigated by RP‐HPLC (Figures S6–S18) and HPLC‐MS (Figures S20–S29). When irradiated at a wavelength of 455 nm, most of the coumarin derivatives showed a fast uncaging by reduction of the starting material peak and two new emerging signals corresponding to the respective payload and the cleaved PPG (Figure 2b). A representative time‐course of the photolysis of compound **3a** is shown in Figure 2c and the corresponding quantum yields are shown in Table 1. The corresponding reaction is shown exemplarily for compound **3a** in Figure 2d. The starting material was usually entirely consumed after 3 min of irradiating at 455 nm (13 mW/cm^2^). Fluoropyrimidines **2a**‐**2c** showed additional peaks after irradiation (Figures S8 and S16) with a similar retention, probably due to an undesired photochemical rearrangement where the PPG shifted from the O^4^ to the N^3^ position of the nucleobase, which is known for this kind of coumarin constructs [99, 101]. A similar behavior could be observed for thioether **1**, which released a side‐product after irradiation. Although some release of the desired **6MP** was visible, the overall photoreaction was incomplete and did not proceed in a clean manner (Figure S7).

In the case of thymidine payloads, an O^4^ to N^3^ shift can sometimes be avoided by using quinone methide‐based self‐immolative linkers (compounds **6** and **7**). Unfortunately, in our case, gemcitabine derivative **7** and floxuridine derivative **6** also showed only an incomplete release of the desired payload after irradiation under the previously mentioned conditions (Figure S15). These results are in contrast to previously reported benzyl linker‐based photocaged compounds [99] which utilized the same PPG, but different payloads like thymidine.

Antifolates **8–10** behaved in a similar fashion. In all three cases multiple photoproducts were visible in the resulting HPLC traces (Figure S17). Unfortunately, only trace amounts of the desired payload were released after extensive irradiation at 455 nm. Since neither the native cytostatic nor the photoproduct **DEACM‐OH** were clearly detectable in these experiments, no quantum yield was determined. These results indicate that instead of a clean photoreaction, photodegradation pathways seem to be dominant for coumarin‐substituted antifolates. This is a surprising result because carboxylic acids, like the glutamate residue, are good leaving groups and are usually released in a clean manner in photoreactions (including coumarins), highlighting the substrate specificity of the applied caging strategy. Due to the unfavorable photolytic properties accompanied by the low solubility in the case of compounds **8–10**, the self‐immolative linker strategy (derivatives **6** and **7**) and the antifolates were not further investigated in the following cytotoxicity studies.

After excluding the products with undesired properties as discussed, compounds **1**, **2c**, **3a**, **3b**, **4a**, **4b**, **5a** and **5b** were considered for further investigations. They were incubated in cell culture medium (DMEM) at 37°C in the presence of cells to investigate their corresponding resistance to hydrolysis (Table 1). Out of the eight tested compounds, floxuridine derivative **5a** and fludarabine derivative **5b** again showed nearly complete hydrolysis, whereas the remaining compounds showed no significant decomposition (Figures S6–S14). Additionally, the uncaging reactions of compounds **2c**, **3a**, **3b**, **4a**, **4b**, **5a** and **5b** could be reconfirmed.

### 2.3 Cell Culture Studies

Initially, two different colon cancer cell lines were considered as a model system for the previously characterized compounds. HT‐29 and HCT‐116 cells were treated with the free coumarin alcohol **DEACM‐OH** and floxuridine to test their respective sensitivities (Figure 3a). Overall, **DEACM‐OH** showed low intrinsic toxicity, reflected in high IC_50_ values of 16.5 ± 9.8 µM for HT‐29 cells and greater than 200 µM for HCT‐116 cells. As expected, floxuridine showed a higher cell toxicity in both cell lines, indicated by the significantly lower IC_50_ values of 42 ± 16 nM (HT‐29) and 400 ± 40 nM (HCT‐116). Due to their higher sensitivity, further experiments were focused on HT‐29 cells. Next, the effect of light on this cancer cell line was tested by irradiation with wavelengths ranging from 365 to 780 nm at a power of 13 mW/cm^2^ for 3 min. This photon flux is sufficient to efficiently photolyse 5 nmol of each previously synthesized compound. As shown in Figure 3b, no phototoxicity was observed under these conditions (even for UV A irradiation).

**FIGURE 3:**
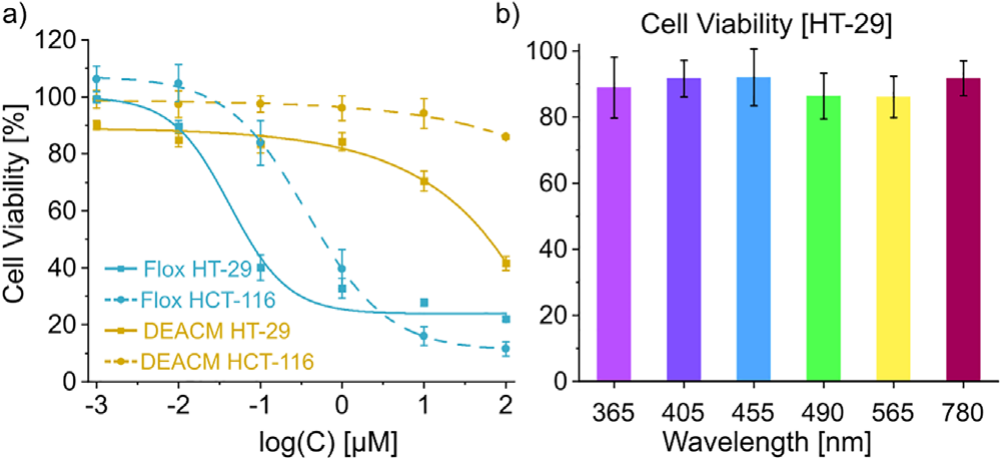
(a) Cell viability of HT‐29 (continuous lines) and HCT‐116 (dashed lines) colon cancer cells in the presence of the native PPG **DEACM‐OH** (yellow curves) and unmodified Floxuridine (**Flox**, blue curves). Concentrations measured from 1 nM–100 µM, cell viability measured by WST‐assay. (b) Cell viability of HT‐29 cells measured by WST‐assay after irradiation with LED‐light (365–780 nm, 13 mW/cm^2^ for 3 min). Cell viability data is presented as the mean + SEM from three independent experiments.

Subsequently, HT‐29 cells were treated with the remaining coumarin derivatives **1**, **2c**, **3a**, **3b**, **4a**, **4b**, **5a**, and **5b** and dose response curves were measured in concentrations ranging from 0.1 nM to 100 µM in the dark (black curves) and after irradiation with blue LEDs (455 nm, 13 mW/cm^2^, red curves) (Figure 4). The dose responses of the corresponding unmodified cytostatics were also measured as a control and are shown as blue curves.

**FIGURE 4:**
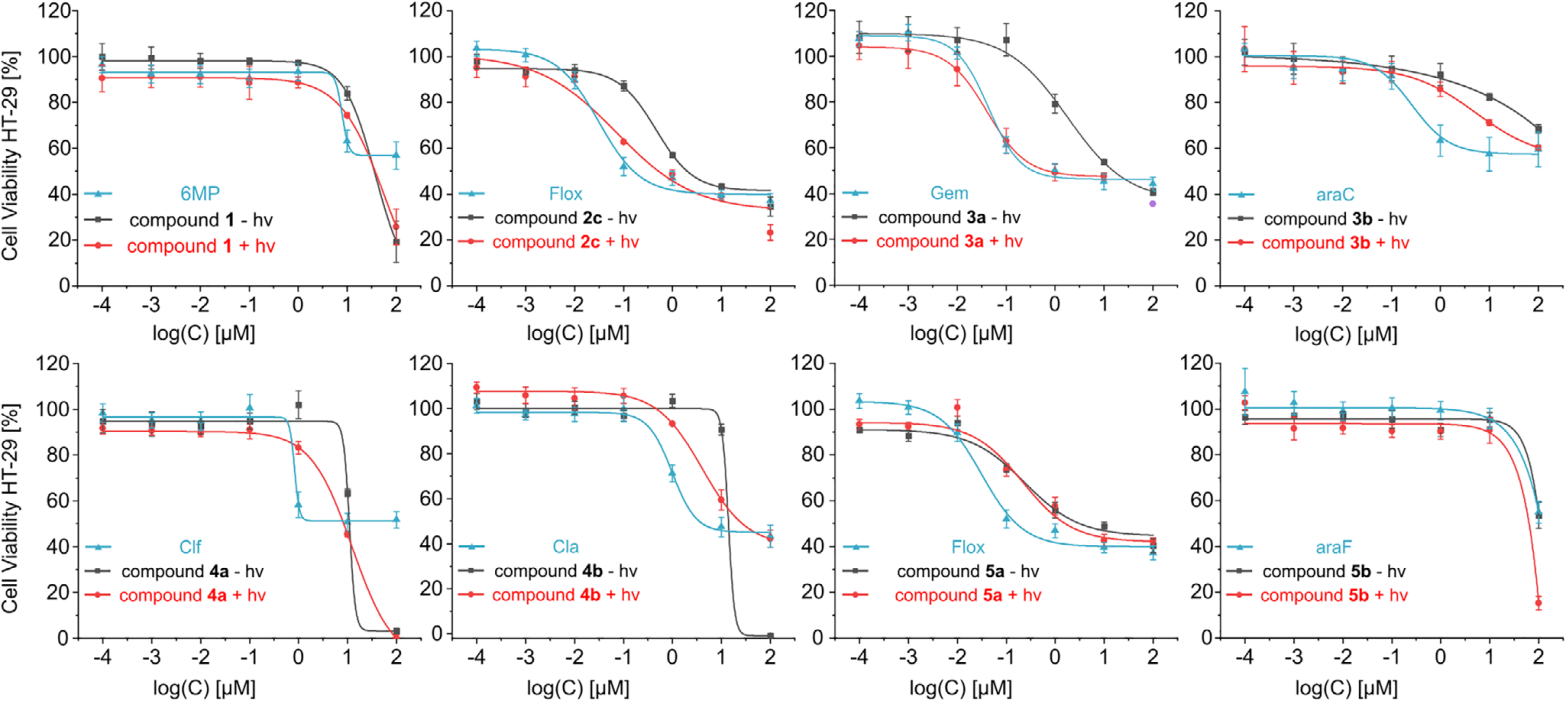
Cell viability of HT‐29 colon cancer cells in the presence of the native cytostatics **Gem**, **6MP**, **araF**, **Cla**, **Clf**, **araC**, **Flox** (blue curves), compounds **1**, **2c**, **3a**, **3b**, **4a**, **4b**, **5a** and **5b** in the dark (black curves) and after irradiation (3 min) at 455 nm (13 mW/cm^2^, red curves). The cell viability was measured by the WST‐assay. Cell viability data is presented as the mean + SEM from three independent experiments. In the case of **3a**, one point (100 µM, red curve, marked in purple) was not used for the determination of the IC_50_‐value.

Under our experimental conditions, HT‐29 colon cancer cells were already quite tolerant to mercaptopurine (**6MP**) and fludarabine (**araF**), observable by no distinct change in the dose response curves of the caged derivatives **1** and **5b** before or after uncaging.

For clofarabine (**Clf**) and cladribine (**Cla**), cytotoxic effects were visible with the investigated cell line, but the IC_50_ values were still relatively high (ca. 1 µM, Table 2). Their photocaged counterparts **4a** and **4b** had an IC_50_ value which was higher by approximately one order of magnitude. However, irradiation only had a moderate effect. We define the phototherapeutic index (PI) as ratio between the IC_50_ values before and after irradiation. For compounds **4a** and **4b**, the PI value was only 1.4 and 3.5, respectively. Cytarabine (**araC**) and floxuridine (**Flox**) both showed submicromolar IC_50_ values. Photocaging raised these IC_50_ values by a factor of 42 (**3b**), 17 (**2c**), and 6 (**5a**), respectively. However, the PI values obtained were only 2.7 (**3b**), 7 (**2c**), and 1 (**5**). Strikingly, there was a large difference between the two photocaged floxuridine derivatives **2c** and **5a**, which were modified on the nucleobase (**2c**) and the deoxyribose (**5a**). This allows the conclusion that nucleobase caging is the superior approach.

**TABLE 2:** IC_50_‐values derived from HT‐29 colon cancer cells treated with compounds **1**, **2c**, **3a**, **3b**, **4a**, **4b**, **5a** and **5b** (Figure 4) in the dark and after irradiation at 455 nm (13 mW/cm^2^), 3 min, compared to the values derived from treatment with unmodified cytostatics (native). PI‐values are derived from the ratio between the IC_50_‐values with irradiation and without irradiation.

Compound	1/6MP	2c/Flox	3a/Gem	3b/araC	4a/Clf	4b/Cla	5a/Flox	5b/araF
After irradiation	>50 µM	0.10 µM	0.035 µM	5.3 µM	10 µM	4.0 µM	0.22 µM	>50 µM
Before irradiation	>50 µM	0.70 µM	1.0 µM	11 µM	14 µM	14 µM	0.24 µM	>50 µM
Native	8.0 µM	0.04 µM	0.04 µM	0.26 µM	0.83 µM	0.98 µM	0.04 µM	>50 µM
PI	—	7.0	29	2.7	1.4	3.5	1	—

The best compound of the series was gemcitabine derivative **3a**, which showed a 40 nM IC_50_ value in HT‐29 cells. A 25‐fold decrease in toxicity upon photocaging was observed, and the activity was fully restored within error limits upon irradiation (**3a**, PI value of 29). This PI value is within the range of previously FDA‐approved photocaged small molecule‐derived drugs [87]. However, the activation of the gemcitabine compound **3a** still requires blue light, which does not penetrate deeply into blood‐perfused tissues. Therefore, as next step, we decided to replace the DEACM photocaging group with a red‐light activatable one.

### 2.4 Red Light‐Release of Gemcitabine

Recent red light‐photoactivation approaches use PPGs based on xanthene derivatives, BODIPY, porphyrin or heptamethine (Cy7) [73, 74, 77, 80, 81, 82, 84, 102, 103]. We adapted a photocaging strategy from Štacko et al. [82] and applied it to our previously established caging protocol (Figure 5). Briefly, starting from gemcitabine, the *para*‐nitrophenol derivative **11a** was used to synthesize the pyridine derivative **11b** in one step with a yield of 42%. Afterwards, the pyridine derivative **11b** was treated with 2,4‐dinitrophenyltriflate to obtain the desired Zincke salt **11c,** which was subsequently converted with 1,2,3,3‐tetramethyl‐3*H*‐indolium iodide to the Cy7‐substituted gemcitabine **11**.

**FIGURE 5:**
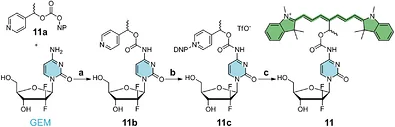
Synthesis of Cy7‐substituted gemcitabine **11**, (a) LiHMDS in THF, −78°C to rt, 42%; (b) 2,4‐dinitrophenyltriflate in ACN, rt, crude; (c) 1,2,3,3‐tetramethy‐3*H*‐indol‐1‐ium iodide, AcOK in MeCN, rt, 5.8%. NP = 4‐nitrophenyl.

Photochemical studies showed a strong absorption in the red region of the spectrum (Figure 6c) with a maximum at 784 nm and an extinction coefficient of 50,300 M^−1^cm^−1^, which is in good agreement with previous reports [80, 82]. Water solubility can often be an issue with large PPGs. Satisfyingly, the Cy7‐caged species (**11**) showed a comparable water solubility to the much smaller coumarin compound **3a** in PBS with no precipitation with just 1% DMSO added as a cosolvent. RP‐HPLC uncaging studies (Figure 6a,b) in aqueous solutions allowed us to determine a quantum yield of 0.015% for this photoreaction. The corresponding HPLC traces showed a fast decrease in the starting material signal (19.7 min) with new signals arising at 17.1 min (uncaging byproduct) and 6.2 min (gemcitabine) retention times when irradiated at a wavelength of either 695 or 780 nm, even in degassed solutions (Figure S18). These results could also be confirmed with ^1^H‐NMR irradiation experiments (Figure S3) in deuterated DMSO/D_2_O as a solvent and HPLC‐MS investigations (Figure S30). Nevertheless, a slight hydrolysis of compound **11** was observed during stability tests (55% remaining after 67 h, Figure S18).

**FIGURE 6:**
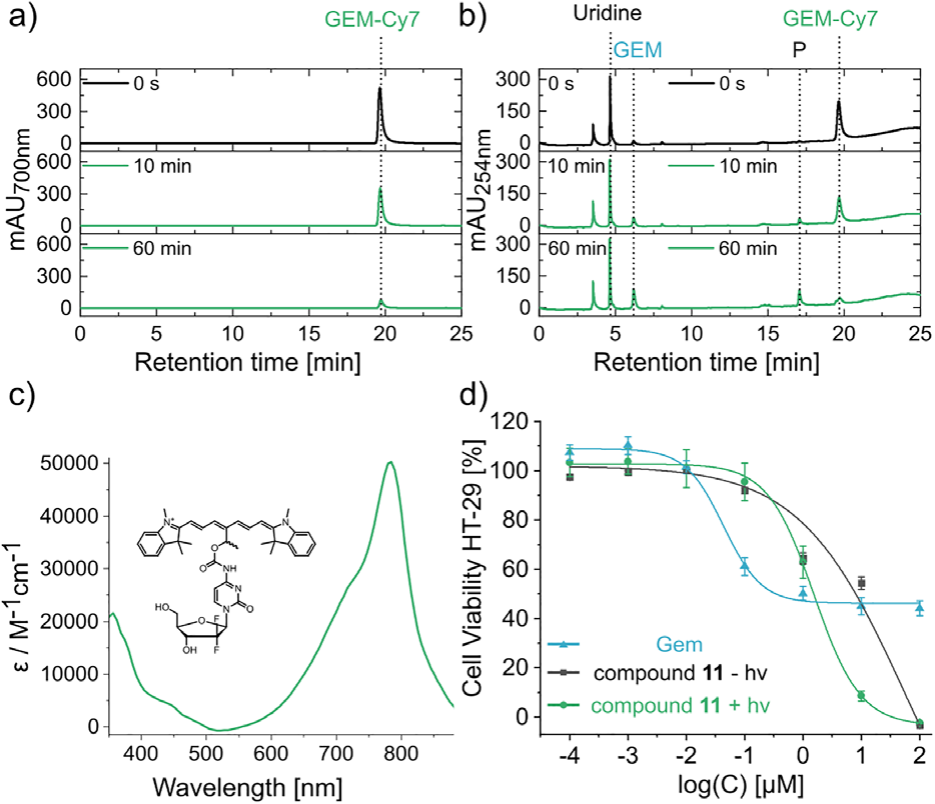
(a) HPLC‐traces of Cy7‐gemcitabine **11** at 700 nm and (b) 254 nm detection, samples (100 µM, PBS x 0.1, 5% DMSO) irradiated for 10 and 60 min with red light (695 nm, 16 mW/cm^2^) are shown in green, whereas a freshly prepared sample is shown in black. (c) Absorption spectrum of **11** (PBS x 0.1, 5% DMSO). (d) Cell viability of HT‐29 cancer cells treated with varying concentrations of compound **11** in DMEM (1% DMSO) kept in the dark (black curve, – hv) and irradiated for 2 h at 695 nm (16 mW/cm^2^, green curve, + hv). Cell viability data is presented as the mean + SEM from three independent experiments.

Then, the same cell studies as before were conducted (Figure 6d). Without irradiation, we determined an IC_50_ value of > 50 µM. After the irradiation of HT‐29 cells in the presence of compound **11** at 695 nm, a dose response with an IC_50_ value of 1.5 µM was determined. With these values, the Cy7‐caged gemcitabine **11** is less toxic than the **DEACM**‐caged gemcitabine **3a** before irradiation. However, after irradiation with red light, the IC_50_ value of unmodified gemcitabine could not be restored.

A possible explanation for this behavior could be the intrinsic high concentration dependence of the photorelease process, which could be observed for this type of Cy7‐PPGs [82]. This could lead to an inefficient photorelease of the desired payload in the nM range required for cell culture studies of gemcitabine. Additionally, it is known for Cy7‐PPG strategies that the overall chemical yield for the release of the desired payload of only 30%–60% as shown in studies by Štacko and Schnermann [77, 80, 81, 82]. Even lower chemical yields of 16% for the release of Arylamine payloads have also been reported [79]. From the integrals of our HPLC analyses, we determine a chemical yield of ca. 30% for the release of gemcitabine, which could explain the observed behavior in Figure 6d. Nevertheless, it was possible to estimate our PI value for compound **11** to be >33. Other studies on red‐light photorelease of cytostatic drugs reported PI value ranges from 2 to 16 [73, 77, 80]. This is the first example of successful red‐light activatable Cy7‐nucleobase photocaging and might find further application in oligonucleotide chemistry.

## 3 Conclusion

In this work, we presented different synthetic approaches to new light‐activatable cytostatics based on different antimetabolite scaffolds like antifolates, pyrimidine, and purine analogs. We prepared and studied sixteen new coumarin‐substituted compounds. These structurally diverse compounds were based on different linker strategies and targeted different functional groups of the previously mentioned anti‐cancer medications. The nucleobase‐photocaged derivatives showed superior results—especially with carbamate linkers for the amine payloads. We investigated uncaging behavior, solubility, and stability, and performed cancer cell toxicity studies with HT‐29 cell lines. Gemcitabine with a blue light‐cleavable coumarin‐derived **DEACM** photocaging group attached to *N*4 of the cytosine ring via a carbamate (**3a**) showed the most favorable overall properties, although the photocaged floxuridine analog **2c** also exhibited clear photoactivatable cytotoxicity. The photocaged DEACM gemcitabine derivative showed uncompromised solubility and stability in buffer and cell medium as well as fast photolysis under blue light irradiation (455 nm). Upon light‐activation, we obtained a 29‐fold increase in IC_50_ value (phototherapeutic index). A red light–activatable Cy7‐photocaged derivative (**11**) was even less toxic prior to irradiation, yet exhibited a >33‐fold increase in IC_50_ upon light activation—among the highest phototherapeutic indices reported for organic PPGs carrying an anticancer payload. We propose that our gemcitabine derivatives are new, promising tools for light‐assisted chemotherapy in the phototherapeutic window.

## 4 Materials and Methods

### 4.1 Reagents and Materials

Antimetabolite cytostatics were purchased from BLDpharm, TCI, or ABCR. Chemical reagents and solvents were purchased from Doug Discovery, TCI, ABCR, Carl Roth, VWR, and Sigma–Aldrich and used without further purification. DMEM (no phenol red), penicillin/streptomycin, sodium pyruvate, and glutamate (Glutamax) were purchased from Gibco/Thermo Fisher, and fetal calf serum (FCS) from Capricorn Scientific.

### 4.2 Chemical Synthesis

Chemical synthesis was conducted in dry solvents under exclusion of water in an argon atmosphere unless stated otherwise. Microwave heating was performed in sealed vessels in a Biotage Initiator microwave reactor (max. microwave power 400 W, frequency 2450 MHz, 220–240 V). Reactions were monitored by TLC (silica gel 60–coated aluminum sheet, UV254 indicator, Macherey‐Nagel), and the crude products were purified by column chromatography with silica gel (silica gel 60, Macherey‐Nagel). Purity of final compounds was then evaluated by NMR‐spectroscopy and RP‐HPLC. Further characterization was performed by mass spectrometry. Low‐resolution electrospray ionization mass spectra were measured on a Thermo Fisher Surveyor MSQ device. High‐resolution mass spectra were obtained on a Thermo Fisher LTQ Orbitrap XL (MALDI‐HRMS) device, a Thermo Fisher Q Exactive Plus device or a Bruker MicroTof‐QII device (ESI‐HRMS).

### 4.3 Cell Culture

Wild‐type colon cancer cell lines HT‐29 and HCT‐116 were purchased from Deutsche Sammlung für Mikroorganismen und Zellkulturen (DSMZ, Braunschweig) and maintained in Dulbecco's modified Eagle medium (DMEM, without phenol red) containing 10% FCS, 100 µg/mL streptomycin and 100 U/mL penicillin, 1% pyruvate, 1% glutamate. The cell lines were cultured at 37°C in the presence of 5% CO_2_ and passaged twice a week.

### 4.4 Cytotoxicity Assay of the Desired Compounds

The HT‐29 or HCT‐116 cancer cells were counted by trypan blue staining with an automated cell counter (TC10 automated cell counter, Bio‐Rad, Munich) and seeded into 96‐well plates (clear polystyrene plates, Greiner Bio‐One GmbH, Frickenhausen) with a density of 5 × 10^3^ cells/well, contained in 100 µL of cell medium (DMEM). After 24 h, the medium was changed to fresh DMEM containing the desired compounds at concentrations ranging from 0.1 nM to 200 µM (stock solutions of compounds in DMSO at 50–100 mM concentrations). As control, the supernatant was exchanged to fresh cell culture medium with nothing or only the cosolvent (DMSO) added. Light‐sensitive solutions were added to the cells in the dark. After the addition of the desired compounds, the plates were directly irradiated with either blue (455 nm, 13 mW/cm^2^) or red (695 nm, 16 mW/cm^2^) light with in‐house built LED‐based irradiation devices or kept entirely in the dark. After 72 h at 37°C in the presence of 5% CO_2_, cell viability was determined utilizing the WST‐1 assay (Roche, distributed by Merck KGaA, Darmstadt, Germany). 10 µL of WST‐1 reagent were added to each well and incubated for 1 h at 37°C. Then, the optical densities of the wells were measured with a TECAN infinite 200 plate reader (TECAN group, Männedorf, Switzerland). Cell viability was calculated as the ratio between the optical densities of the wells containing compounds and to that of the untreated wells containing only cell culture medium. The obtained values were averaged between 3–6 wells of the same condition and repeated at three different days (*n* = 3).

### 4.5 Stability Assay of Target Compounds in the Presence of HT‐29 Cells

As described above, HT‐29 cancer cells were seeded into 96‐well plates with a density of 5 × 10^3^ cells/well contained in 100 µL of cell medium (DMEM). After 24 h, the medium was renewed to fresh DMEM containing the desired compounds at a concentration of 100 µM (stock solutions of compounds in DMSO at 50–100 mM concentrations). Light‐sensitive solutions were added to the cells in the dark. After the addition of the desired compounds, plates were directly irradiated with either blue (455 nm, 13 mW/cm^2^) light with an in‐house built LED‐based irradiation device (*vide supra*) or kept entirely in the dark. After 72 h at 37°C in the presence of 5% CO_2_, the supernatants were removed, wells were washed with 100 µL of PBS and the combined solutions were subjected to a C_18_ solid phase extraction (adapted from *Werz and Steinhilber* [104], conducted only in the presence of red light) to remove water‐soluble compounds. The aqueous solutions were applied to a C_18_ solid phase column preconditioned with 1 mL of methanol and 1 mL of water, and then washed afterwards with 1 mL of water and 1 mL of 25% methanol. The organic compounds were then eluted with 300 µL of methanol and analyzed by RP‐HPLC.

### 4.6 RP‐High‐Performance Liquid Chromatography

High‐performance liquid chromatography (RP‐HPLC) was conducted with a 1260 infinity LC system (Agilent Technologies) equipped with a quaternary pump, automatic liquid sampler, temperature‐controlled column compartment, and a diode array detector. A MultoKrom 100–5 C_18_ column (250 × 4.6 mm) from CS‐Chromatographie Service GmbH was used for separation with a mixture of acetonitrile and 100 mM NH_4_OAc‐buffer in ultra‐pure water (pH 7) as an eluent (concentration gradient between 5%–100% acetonitrile).

### 4.7 Photochemical Experiments

The UV‐Vis spectra were measured with an Ocean Optics USB4000 detector connected with an optical fiber and convex lens, mounted in an adapter, to a CVH100 cuvette holder (Thorlabs). On the opposing side of the holder, a DH‐mini light source (Ocean Optics) was connected in the same way. Measurements were conducted in 1.0 cm quartz fluorescence cuvettes (QS) from Hellma–Analytics with mixtures of 0.1 x PBS and p.a. grade DMSO used as solvents. The results were evaluated using an in‐house programmed software (*PHITS*, Photoswitch Irradiator Test Suite), which is based on LabVIEW [105]. Extinction coefficients were determined by measuring dilution series of the desired compounds with at least four different concentrations in an optical density range between 0.1 and 1. The resulting data sets were then baseline corrected by subtracting the absorption minimum in a region where no absorption was detectable (700 nm in most cases), and the extinction coefficients were calculated according to Lambert–Beer's law.

Irradiation experiments were conducted with the desired compounds in concentration ranges of 25–200 µM with uridine added as an internal standard in mixtures of 0.1 x PBS and p.a. grade DMSO as solvents (between 5%–20% DMSO content) in polystyrene 96‐well plates (clear polystyrene plates, Greiner Bio‐One GmbH, Frickenhausen). The samples were irradiated through the polystyrene cover of the plate with the same in‐house built LED light sources (SI) as used in cell culture at either 455 nm (13 mW/cm^2^), 695 nm (16 mW/cm^2^), or with a 780 nm‐LED manufactured by Thorlabs and then subjected to RP‐HPLC as described above.

Hydrolysis studies were done with the same solutions (kept in the dark) at room temperature for several days. Then, the samples were subjected to the same HPLC analysis.

## Conflicts of Interest

The authors declare no conflicts of interest.

## Supporting information




**Supporting File**: The authors have cited additional references within the Supporting Information [106, 107, 108, 109, 110, 111, 112, 113, 114, 115, 116, 117, 118].

## Data Availability

The data that supports the findings of this study are available in the supplementary material of this article
