# Supplementary material for: Control of Gemcitabine Activity With Blue and Red Light
Source: Chemistry. 2026 Jul 6;32(38):e71363. doi: 10.1002/chem.71363 (PMC13660501; doi:10.1002/chem.71363)
Supplement: Supplementary file 1 [file CHEM-32-e71363-s001.docx]

Supporting Information

Control of Gemcitabine Activity with Blue and Red Light

Nils F. Kersten^[a]^, Konstantin L. Stock^[b]^, Marius Hyprath^[c]^, Hannah Weisser^[c]^, Timo Weinrich^[a]^, Dieter Steinhilber*^[c]^, Alexander Heckel*^[a]^

[a]	N. F. Kersten, Dr. T. Weinrich, Prof. Dr. A. Heckel
Institute for Organic Chemistry and Chemical Biology
Goethe-University
Max-von-Laue-Str. 7, D-60438 Frankfurt am Main, Germany
E-mail: [heckel@uni-frankfurt.de](mailto:heckel@uni-frankfurt.de)

[b]	K. L. Stock
Department of Chemistry, and FRQNT Centre for Green Chemistry and Catalysis
McGill University
801 Sherbrooke Street West, Montreal, QC H3A 0B8, Canada

[c]	M. Hyprath, Dr. H. Weisser, Prof. Dr. D. Steinhilber
Institute of Pharmaceutical Chemistry
Goethe-University
Max-von-Laue-Str. 9, D-60438 Frankfurt am Main, Germany
E-mail: [steinhilber@em.uni-frankfurt.de](mailto:steinhilber@em.uni-frankfurt.de)

**Contents**

[1.	Fluorescence Images of HT-29 Cells incubated with Compounds 1-5	8](#_Toc220086322)

[2.	Dose-Response of 5-Fluorouracil (5-FU)	8](#_Toc220086323)

[3.	Investigation of Photolysis by NMR Spectroscopy	9](#_Toc220086324)

[4.	Irradiation Sources	10](#_Toc220086325)

[4.1 In-House built Devices	10](#_Toc220086326)

[4.2 Other LEDs used in this Study	11](#_Toc220086327)

[5.	HPLC-based Investigation of (photo-)chemical Properties	12](#_Toc220086328)

[6.	HPLC-MS Investigation of the Photorelease	19](#_Toc220086329)

[7.	Synthesis of the Light activatable Cytostatics	26](#_Toc220086330)

[7.1 Synthetic Overview	26](#_Toc220086331)

[7.2. Synthetic Materials and Methods	28](#_Toc220086332)

[7.3	General Synthetic Procedures	29](#_Toc220086333)

[7.3.1	General Procedure A for TBDMS Protection	29](#_Toc220086334)

[7.3.2	General Procedure B for Carbamate Formation	29](#_Toc220086335)

[7.3.3	General Procedure C for TBDMS Deprotection	29](#_Toc220086336)

[7.3.4	General Procedure D for Steglich Esterification	29](#_Toc220086337)

[7.4	Chemical Synthesis	30](#_Toc220086338)

[7.4.1 4-(((9*H*-Purin-6-yl)thio)methyl)-7-(diethylamino)-2*H*-chromen-2-one (6MP-DEACM, 1)	30](#_Toc220086339)

[7.4.2 5-Fluoro-1-(tetrahydrofuran-2-yl)-4-(1*H*-1,2,4-triazol-1-yl)pyrimidin-2(1*H*)-one (Tegafur-Triazole, 2b.1)	30](#_Toc220086340)

[7.4.3 4-((7-(Diethylamino)-2-oxo-2*H*-chromen-4-yl)methoxy)-5-fluoro-1-(tetrahydrofuran-2-yl)pyrimidin-2(1*H*)-one (DEACM-tegafur, 2b)	31](#_Toc220086341)

[7.4.4 4-((7-(Diethylamino)-2-oxo-2*H*-chromen-4-yl)methoxy)-5-fluoropyrimidin-2(1*H*)-one (DEACM-5FU, 2a)	32](#_Toc220086342)

[7.4.5 1‑((2*R*,4*S*,5*R*)‑4‑((*tert*‑Butyldimethylsilyl)oxy)‑5‑(((*tert*‑butyldimethylsilyl)oxy)methyl)tetrahydrofuran-2-yl)-5-fluoropyrimidine-2,4(1*H*,*3H*)-dione (TBDMS-Floxuridine)	33](#_Toc220086343)

[7.4.6 1‑((2*R*,4*S*,5*R*)‑4‑((*tert*‑Butyldimethylsilyl)oxy)‑5‑(((*tert*‑butyldimethylsilyl)oxy)methyl)tetrahydrofuran-2-yl)-5-fluoro-4-(1*H*-1,2,4-triazol-1-yl)pyrimidin-2(1*H*)-one (Triazoyl-TBDMS-Floxuridine, 2c.1)	33](#_Toc220086344)

[7.4.7 1‑((2*R*,4*S*,5*R*)‑4‑((*tert*‑Butyldimethylsilyl)oxy)‑5‑(((*tert*‑butyldimethylsilyl)oxy)methyl)tetrahydrofuran-2-yl)-4-((7-(diethylamino)-2-oxo-2*H*-chromen-4-yl)methoxy)-5-fluoropyrimidin-2(1*H*)-one (TBDMS-DEACM-Floxuridine, 2c.2)	34](#_Toc220086345)

[7.4.8 4‑((7‑(Diethylamino)‑2‑oxo‑2*H*‑chromen‑4‑yl)methoxy)‑5‑fluoro‑1‑((2*R*,4*S*,*5R*)‑4-hydroxy-5-(hydroxymethyl)tetrahydrofuran-2-yl)pyrimidin-2(1*H*)-one (DEACM-Floxuridine, 2c)	35](#_Toc220086346)

[7.4.9 1‑((2*R*,4*S*,5*R*)‑4‑((*tert*‑Butyldimethylsilyl)oxy)‑5‑(((*tert*‑butyldimethylsilyl)oxy)methyl)tetrahydrofuran‑2‑yl)‑4‑((4‑((7‑(diethylamino)‑2‑oxo‑2*H*‑chromen‑4‑yl)methoxy)benzyl)oxy)-5-fluoropyrimidin-2(1*H*)-one (TBDMS-DEACM-Bn-Floxuridine, 6.2)	36](#_Toc220086347)

[7.4.10 4‑((4‑((7‑(Diethylamino)‑2‑oxo‑2*H*‑chromen‑4‑yl)methoxy)benzyl)oxy)‑5‑fluoro‑1-((2*R*,4*S*,5*R*)-4-hydroxy-5-(hydroxymethyl)tetrahydrofuran-2-yl)pyrimidin-2(1*H*)-one (DEACM-Bn-Floxuridine, 6)	37](#_Toc220086348)

[7.4.11 (7-(Diethylamino)-2-oxo-2*H*-chromen-4-yl)methyl(((2*R*,3*S*,5*R*)-5-(5-fluoro-2,4-dioxo-3,4-dihydropyrimidin-1(2*H*)-yl)-3-hydroxytetrahydrofuran-2-yl)methyl) carbonate (5’-DEACM-Floxuridine, 5a)	38](#_Toc220086349)

[7.4.12 4‑Amino‑1‑((2*R*,4*R*,5*R*)‑4‑((*tert*‑butyldimethylsilyl)oxy)‑5‑(((*tert*‑butyldimethylsilyl)oxy)methyl)-3,3-difluorotetrahydrofuran-2-yl)pyrimidin-2(1*H*)-one (TBDMS-Gemcitabine, 3a.1)	39](#_Toc220086350)

[7.4.13 4‑Amino‑1‑((2*R*,4*S*,5*R*)‑4‑((*tert*‑butyldimethylsilyl)oxy)‑5‑(((*tert*‑butyldimethylsilyl)oxy)methyl)tetrahydrofuran-2-yl)-1,3,5-triazin-2(1*H*)-one (TBDMS-Decitabine, 3c.1)	39](#_Toc220086351)

[7.4.14 (7‑(Diethylamino)‑2‑oxo‑2*H*‑chromen‑4‑yl)methyl (1‑((2*R*,4*R*,5*R*)‑4‑((*tert*‑butyldimethylsilyl)oxy)‑5‑(((*tert*‑butyldimethylsilyl)oxy)methyl)‑3,3‑difluorotetrahydrofuran-2-yl)-2-oxo-1,2-dihydropyrimidin-4-yl)carbamate (TBDMS-DEACM-Gemcitabine, 3a.2)	40](#_Toc220086352)

[7.4.15 4-((7-(Diethylamino)-2-oxo-2*H*-chromen-4-yl)methoxy)benzyl 1*H*-imidazole-1-carboxylate (DEACM-Bn-CDI)	41](#_Toc220086353)

[7.4.16 4‑((7‑(Diethylamino)‑2‑oxo‑2H‑chromen‑4‑yl)methoxy)benzyl (1‑((2*R*,4*R*,*5R*)-4-((*tert*-butyldimethylsilyl)oxy)-5-(((*tert*-butyldimethylsilyl)oxy)methyl)-3,3-difluorotetrahydrofuran-2-yl)-2-oxo-1,2-dihydropyrimidin-4-yl)carbamate (TBDMS-DEACM-Bn-Gemcitabine, 7.1)	42](#_Toc220086354)

[7.4.17 (7-(Diethylamino)-2-oxo-2*H*-chromen-4-yl)methyl (5-((2*R*,4*S*,*5R*)-4-((*tert*-butyldimethylsilyl)oxy)-5-(((*tert*-butyldimethylsilyl)oxy)methyl)tetrahydrofuran-2-yl)-4-oxo-4,5-dihydro-1,3,5-triazin-2-yl)carbamate (TBDMS-DEACM-Decitabine, 3c.2)	43](#_Toc220086355)

[7.4.18 (7-(Diethylamino)-2-oxo-2*H*-chromen-4-yl)methyl (1-((2*R*,4*R*,5*R*)-3,3-difluoro-4-hydroxy-5-(hydroxymethyl)tetrahydrofuran-2-yl)-2-oxo-1,2-dihydropyrimidin-4-yl)carbamate (DEACM-Gemcitabine, 3a)	43](#_Toc220086356)

[7.4.19 4-((7-(Diethylamino)-2-oxo-2*H*-chromen-4-yl)methoxy)benzyl (1-((2*R*,4*R*, 5*R*)-3,3-difluoro-4-hydroxy-5-(hydroxymethyl)tetrahydrofuran-2-yl)-2-oxo-1,2-dihydropyrimidin-4-yl)carbamate (DEACM-Bn-Gemcitabine, 7)	44](#_Toc220086357)

[7.4.20 (7-(Diethylamino)-2-oxo-2*H*-chromen-4-yl)methyl (5-((2*R*,4*S*,5*R*)-4‑hydroxy-5-(hydroxymethyl)tetrahydrofuran-2-yl)-4-oxo-4,5-dihydro-1,3,5-triazin-2-yl)carbamate (DEACM-decitabine, 3c)	45](#_Toc220086358)

[7.4.21 (7-(Diethylamino)-2-oxo-2*H*-chromen-4-yl)methyl (1-((2*R*,3*S*,4*S*,5*R*)-3,4-dihydroxy-5-(hydroxymethyl)tetrahydrofuran-2-yl)-2-oxo-1,2-dihydropyrimidin-4-yl)carbamate (DEACM-Cytarabine, 3b)	46](#_Toc220086359)

[7.4.22 9‑((2*R*,3*S*,4*R*,5*R*)‑4‑((*tert*‑Butyldimethylsilyl)oxy)‑5‑(((*tert‑*butyldimethylsilyl)oxy)methyl)-3-fluorotetrahydrofuran-2-yl)-2-chloro-9*H*-purin-6-amine (TBDMS-Clofarabine, 4a.1)	46](#_Toc220086360)

[7.4.23 9‑((2*R*,4*S*,5*R*)‑4‑((t*ert*‑Butyldimethylsilyl)oxy)‑5‑(((*tert*‑butyldimethylsilyl)oxy)methyl)tetrahydrofuran-2-yl)-2-chloro-9*H*-purin-6-amine (TBDMS-Cladribine, 4b.1)	47](#_Toc220086361)

[7.4.24 (7-(Diethylamino)-2-oxo-2*H*-chromen-4-yl)methyl (9-((2*R*,3*S*,4*R*,5*R*)-4-((*tert*‑butyldimethylsilyl)oxy)‑5‑(((*tert*‑butyldimethylsilyl)oxy)methyl)‑3‑fluorotetrahydrofuran-2-yl)-2-chloro-9*H*-purin-6-yl) carbamate (TBDMS-DEACM-Clofarabine, 4a.2)	48](#_Toc220086362)

[7.4.25 (7-(Diethylamino)-2-oxo-2*H*-chromen-4-yl)methyl (9-((2*R*,4*S*,5*R*)-4-((*tert*-butyldimethylsilyl)oxy)-5-(((*tert*-butyldimethylsilyl)oxy)methyl)tetrahydrofuran-2-yl)-2-chloro-9*H*-purin-6-yl)carbamate (TBDMS DEACM-Cladribine, 4b.2)	49](#_Toc220086363)

[7.4.26 (7-(Diethylamino)-2-oxo-2*H*-chromen-4-yl)methyl (2-chloro-9-((2*R*,3*S*,4*R*, 5*R*)-3-fluoro-4-hydroxy-5-(hydroxymethyl)tetrahydrofuran-2-yl)-9*H*-purin-6-yl) carbamate (DEACM-Clofarabine, 4a)	50](#_Toc220086364)

[7.4.27 (7-(Diethylamino)-2-oxo-2*H*-chromen-4-yl)methyl (2-chloro-9-((2*R*,4*S*,5*R*)-4-hydroxy-5-(hydroxymethyl)tetrahydrofuran-2-yl)-9*H*-purin-6-yl)carbamate (DEACM-Cladribine, 4b)	50](#_Toc220086365)

[7.4.28 ((2*R*,3*R*,4*S*,5*R*)-5-(6-Amino-2-fluoro-9*H*-purin-9-yl)-3,4-bis((*tert*-butyldi-methylsilyl)oxy)tetrahydrofuran-2-yl)methanol (TBDMS-Fludarabine, 5b.1)	51](#_Toc220086366)

[7.4.29 ((2*R*,3*R*,4*S*,5*R*)-5-(6-Amino-2-fluoro-9*H*-purin-9-yl)-3,4-bis((*tert*-butyldi-methylsilyl)oxy)tetrahydrofuran-2-yl)methyl 1*H*-imidazole-1-carboxylate (TBDMS-CDI-Fludarabine, 5b.2 )	52](#_Toc220086367)

[7.4.30 ((2*R*,3*R*,4*S*,5*R*)-5-(6-Amino-2-fluoro-9*H*-purin-9-yl)-3,4-bis((*tert*-butyldime-thylsilyl)oxy)tetrahydrofuran-2-yl)methyl ((7-(diethylamino)-2-oxo-2*H*-chrom-en-4-yl)methyl) carbonate (TBDMS-DEACM-Fludarabine, 5b.3)	52](#_Toc220086368)

[7.4.31 ((2*R*,3*S*,4*S*,5*R*)-5-(6-Amino-2-fluoro-9*H*-purin-9-yl)-3,4-dihydroxytetra-hydrofuran-2-yl)methyl ((7-(diethylamino)-2-oxo-2*H*-chromen-4-yl)methyl) carbonate (DEACM-Fludarabine, 5b)	53](#_Toc220086369)

[7.4.32 (*S*)-Bis((7-(Diethylamino)-2-oxo-2*H*-chromen-4-yl)methyl) 2-(4-(((2,4-diamino‑pteridin‑6‑yl)methyl)(methyl)amino)benzamido)pentanedioate (DEACM‑Methotrexate, 8)	54](#_Toc220086370)

[7.4.33 (*S*)-Bis((7-(Diethylamino)-2-oxo-2*H*-chromen-4-yl)methyl) 2-(5-(methyl((2-methyl-4-oxo-1,4-dihydroquinazolin-6-yl)methyl)amino)thiophene-2-carbox-amido)pentanedioate (DEACM-Raltitrexed, 9)	55](#_Toc220086371)

[7.4.34 (*S*)-Bis((7-(Diethylamino)-2-oxo-2*H*-chromen-4-yl)methyl) 2-(4-(2-(2-amino-4-oxo-4,7-dihydro-3*H*-pyrrolo[2,3-*d*]pyrimidin-5‑yl)ethyl)benzamido)pentanedi-oate (DEACM-Pemetrexed, 10)	56](#_Toc220086372)

[7.4.35 1‑(Pyridin‑4‑yl)ethyl(1‑((2*R*,4*R*,5*R*)‑3,3‑difluoro‑4‑hydroxy‑5‑(hydroxymethyl)tetrahydrofuran‑2‑yl)‑2‑oxo‑1,2‑dihydropyrimidin‑4‑yl)carbamate (Gemcitabine-Pyridine, 11a)	57](#_Toc220086373)

[7.4.36 4-(1-(((1-((2*R*,4*R*,5*R*)-3,3-Difluoro-4-hydroxy-5-(hydroxymethyl)tetrahydro-furan‑2‑yl)‑2‑oxo‑1,2‑dihydropyrimidin‑4‑yl)carbamoyl)oxy)ethyl)‑1‑(2,4‑dinitro-phenyl)pyridin-1-ium triflate (Gemcitabine-zincke salt, 11b)	58](#_Toc220086374)

[7.4.37 2-((1*E*,3*Z*,5*E*,7*E*)-4-(1-(((1-((2*R*,4*R*,5*R*)-3,3-Difluoro-4-hydroxy-5-(hydroxy-methyl) tetrahydrofuran-2-yl)-2-oxo-1,2-dihydropyrimidin-4‑yl)carbamoyl)oxy) ethyl)-7-(1,3,3-trimethylindolin-2-ylidene)hepta-1,3,5-trien-1-yl)-1,3,3-trimethyl-3*H*-indol-1-ium (Gem-Cy-tabine, 11)	58](#_Toc220086375)

[8	Appendix	60](#_Toc220086376)

[8.3	NMR Spectra	60](#_Toc220086377)

[8.3.1	6MP-DEACM, Compound 1	60](#_Toc220086378)

[8.3.2	Tegafur-Triazole, Compound 2b.1	61](#_Toc220086379)

[8.3.3	DEACM-Tegafur, Compound 2b	62](#_Toc220086380)

[8.3.4	DEACM-5FU, Compound 2a	64](#_Toc220086381)

[8.3.5	TBDMS-DEACM-Floxuridine, Compound 2c.2	65](#_Toc220086382)

[8.3.6	DEACM-Floxuridine, Compound 2c	67](#_Toc220086383)

[8.3.7	TBDMS-DEACM-Bn-Floxuridine, Compound 6.2	68](#_Toc220086384)

[8.3.8	DEACM-Bn-Floxuridine, Compound 6	70](#_Toc220086385)

[8.3.9	5’-DEACM-Floxuridine, Compound 5a	71](#_Toc220086386)

[8.3.10	TBDMS-Gemcitabine, Compound 3a.1	73](#_Toc220086387)

[8.3.11	TBDMS-Decitabine, Compound 3c.1	74](#_Toc220086388)

[8.3.12	TBDMS-DEACM-Gemcitabine, Compound 3a.2	75](#_Toc220086389)

[8.3.13	DEACM-Bn-CDI	77](#_Toc220086390)

[8.3.14	TBDMS-DEACM-Bn-Gemcitabine, Compound 7.1	78](#_Toc220086391)

[8.3.15	TBDMS-DEACM-Decitabine, Compound 3c.2	79](#_Toc220086392)

[8.3.16	DEACM-Gemcitabine, Compound 3a	80](#_Toc220086393)

[8.3.17	DEACM-Bn-Gemcitabine, Compound 7	82](#_Toc220086394)

[8.3.18	DEACM-Cytarabine, Compound 3b	83](#_Toc220086395)

[8.3.19	TBDMS-Clofarabine, Compound 4a.1	84](#_Toc220086396)

[8.3.20	TBDMS-Cladribine, Compound 4b.1	86](#_Toc220086397)

[8.3.21	TBDMS-DEACM-Clofarabine, Compound 4a.2	87](#_Toc220086398)

[8.3.22	TBDMS DEACM-Cladribine, Compound 4b.2	88](#_Toc220086399)

[8.3.23	DEACM-Clofarabine, Compound 4a	90](#_Toc220086400)

[8.3.24	DEACM-Cladribine, Compound 4b	91](#_Toc220086401)

[8.3.25	TBDMS-Fludarabine, Compound 5b.1	92](#_Toc220086402)

[8.3.26	TBDMS-CDI-Fludarabine, Compound 5b.2	94](#_Toc220086403)

[8.3.27	TBDMS-DEACM-Fludarabine, Compound 5b.3	95](#_Toc220086404)

[8.3.28	DEACM-Fludarabine, Compound 5b	97](#_Toc220086405)

[8.3.29	DEACM‑Methotrexate, Compound 8	98](#_Toc220086406)

[8.3.30	DEACM-Raltitrexed, Compound 9	99](#_Toc220086407)

[8.3.31	DEACM-Pemetrexed, Compound 10	100](#_Toc220086408)

[8.3.32	Gemcitabine-Pyridine, Compound 11a	101](#_Toc220086409)

[8.3.33	Gem-Cy-tabine, Compound 11	103](#_Toc220086410)

[8.4	MS Spectra	105](#_Toc220086411)

[8.4.1	DEACM-6MP, Compound 1	105](#_Toc220086412)

[8.4.2	Tegafur-Triazole, Compound 2b.1	105](#_Toc220086413)

[8.4.3	DEACM-tegafur, Compound 2b	106](#_Toc220086414)

[8.4.4	DEACM-5FU, Compound 2a	106](#_Toc220086415)

[8.4.5	TBDMS-DEACM-Floxuridine, Compound 2c.2	107](#_Toc220086416)

[8.4.6	DEACM-Floxuridine, Compound 2c	107](#_Toc220086417)

[8.4.7	TBDMS-DEACM-Bn-Floxuridine, Compound 6.2	107](#_Toc220086418)

[8.4.8	DEACM-Bn-Floxuridine, Compound 6	108](#_Toc220086419)

[8.4.9	5’-DEACM-Floxuridine, Compound 5a	108](#_Toc220086420)

[8.4.10	TBDMS-Gemcitabine, Compound 3a.1	108](#_Toc220086421)

[8.4.11	TBDMS-Decitabine, Compound 3c.1	109](#_Toc220086422)

[8.4.12	TBDMS-DEACM-Gemcitabine, Compound 3a.2	109](#_Toc220086423)

[8.4.13	DEACM-Bn-CDI	109](#_Toc220086424)

[8.4.14	TBDMS DEACM-Bn-Gem	110](#_Toc220086425)

[8.4.15	TBDMS-DEACM-Decitabine, Compound 3c.2	110](#_Toc220086426)

[8.4.16	DEACM-Gemcitabine, Compound 3a	110](#_Toc220086427)

[8.4.17	DEACM-Bn-Gemcitabine, Compound 7	111](#_Toc220086428)

[8.4.18	DEACM-Cytarabine, Compound 3b	111](#_Toc220086429)

[8.4.19	TBDMS-Clofarabine, Compound 4a.1	111](#_Toc220086430)

[8.4.20	TBDMS-Cladribine, Compound 4b.1	112](#_Toc220086431)

[8.4.21	TBDMS-DEACM-Clofarabine, Compound 4a.2	112](#_Toc220086432)

[8.4.22	TBDMS-DEACM-Cladribine, Compound 4b.2	112](#_Toc220086433)

[8.4.23	DEACM-Clofarabine, Compound 4a	112](#_Toc220086434)

[8.4.24	DEACM-Cladribine, Compound 4b	113](#_Toc220086435)

[8.4.25	TBDMS-Fludarabine, Compound 5b.1	113](#_Toc220086436)

[8.4.26	TBDMS-CDI-Fludarabine, Compound 5b.2	113](#_Toc220086437)

[8.4.27	TBDMS-DEACM-Fludarabine, Compound 5b.3	114](#_Toc220086438)

[8.4.28	DEACM-Fludarabine, Compound 5b	114](#_Toc220086439)

[8.4.29	DEACM-Methotrexate, Compound 8	115](#_Toc220086440)

[8.4.30	DEACM-Raltitrexed, Compound 9	115](#_Toc220086441)

[8.4.31	DEACM-Pemetrexed, Compound 10	116](#_Toc220086442)

[8.4.32	Gemcitabine-Pyridine, Compound 11a	116](#_Toc220086443)

[8.4.33	Gemcitabine-zincke salt, Compound 11b	116](#_Toc220086444)

[8.4.34	Gem-Cy-tabine, Compound 11	116](#_Toc220086445)

[9.	References	117](#_Toc220086446)

# 1. Fluorescence Images of HT-29 Cells incubated with Compounds 1-5

HT-29 cancer cells were seeded into 96-well plates with a concentration of 5 x 10^3^ cells/well contained in 100 µL of cell medium (DMEM). After 24 h, the medium was exchanged to fresh DMEM containing the desired compounds at a concentration of 200 µM (stock solutions of compounds in DMSO at 50-100 mM concentrations) which was done in the dark. After an incubation period of 4 h at 37 °C in the presence of 5% CO_2_, the supernatant was removed, the wells were washed with PBS and transmitted light and fluorescence images were then taken with a Zeiss AX10 microscope (Zeiss E-PL 10x/20 eyepiece and Zeiss LD A-Plan 40x/0.55 objective were used) and attached Zeiss Axiocam 305 color imaging system. For the fluorescence images of coumarin-derivatives, a GFP filter was used.

Figure S1: Fluorescence images of HT-29 cells incubated with compounds **1**-**5** or **DEACM-OH** at a concentration of 200 µM in DMEM for 4 hours. Transmitted light images (TL) are shown at the top and fluorescence pictures taken with a GFP filter are shown at the bottom. Measured at 400X magnification.

# 2. Dose-Response of 5-Fluorouracil (5-FU)

Figure S2: Cell viability of HT-29 (continuous lines) and HCT-116 (dashed lines) colon cancer cells in DMEM in the presence of the native 5-Fluorouracil (**5FU**, blue curves). Concentrations measured from 0.1 nM - 200 µM, cell viability measured by WST-assay. The resulting IC_50_-values were: 16 ± 11 µM (HT-29) and 4.3 ± 0.8 µM (HCT-116). Due to the better response of the floxuridine payload (0.04 µM for HT-29) compound **2a** was not investigated in further in cell studies.

# 3. Investigation of Photolysis by NMR Spectroscopy

Two samples of the desired compound were prepared in a mixture of D_2_O and either DMSO-*d*_6_. Cy7-Gemcitabine derivative **11** was irradiated extensively with a 780 nm LED (Thorlabs, M780L3, operated at 800 mA, 1600 mW, driven by Thorlabs LED driver DC4104 with DC4100-Hub attached) focussed with Thorlabs ACL2520-A Lens.

Figure S3: ^1^H-NMR-spectra of the Irradiation of Cy7-Gemcitabine derivative **11** at 780 nm in D_2_O with 20 % of DMSO-*d*_6_. Gemcitabine signals are highlighted in green.

# 4. Irradiation Sources

## 4.1 In-House built Devices

Two in-house built devices utilizing red (695 nm) or blue (455 nm) LEDs were used in the presented irradiation studies.

Blue light device:

Consisting of 96 generic 5 mm clear lens blue light LEDs (30 ° viewing angle, sourced from *Chanzon*) and driven with a forward voltage of 3.0 – 3.2 V at 20 mA (generic LED driver, input: 100-240 V / ≈ 50 ‑ 60 Hz, output: 12.0 V, 1.0 A). The LEDs were soldered to a PCB with a geometry resembling polystyrene 96-well plates (clear polystyrene plates, *Greiner* Bio-One GmbH, Frickenhausen). The power density was measured at 13 mW/cm^2^ (determined with a PowerMax PS10Q detector connected to a LabMax-TO laser power meter from *Coherent*).

Figure S4: In-house built blue light LED-device.

Red light device:

Consisting of 3 x 3W star LEDs (695 nm) mounted in a geometry for 6-well plates on an aluminum heatsink and a 3d-printed spacer attached. The LEDs were driven with an in-house built driver (input: 230 V / 0.5 A / 47 ‑ 63 Hz, output (max): 32.0 V DC, 660 mA) at maximum intensity which resulted in a power density of 16 mW/cm^2^ (determined with a PowerMax PS10Q detector connected to a LabMax-TO laser power meter from *Coherent*). For 96 well plate irradiations in cell culture, the device was rotated every 30 minutes to ensure uniform intensities.

Figure S5: In-house built red light LED-device with the in-house built driver attached.

## 4.2 Other LEDs used in this Study

Thorlabs LEDs were tuned to a power density of 13 mW/cm^2^ (with the previously mentioned power meter) the following LEDs were used and driven by a Thorlabs LED driver DC4104 (with DC4100-Hub attached) focussed with a Thorlabs ACL2520-A Lens:

365L2 (365 nm, maximal output: 310 mW (700 mA) operated at 433 mA)

405L2 (405 nm, maximal output: 1000 mW (1000 mA) operated at 147 mA)

455L2 (455 nm, maximal output: 1800 mW (1600 mA) operated at 232 mA)

490L2 (490 nm, maximal output: 235 mW (350 mA) operated at 57 mA)

565L3 (565 nm, maximal output: 880 mW (1000 mA) operated at 188 mA)

780L3 (780 nm, maximal output: 200 mW (800 mA) operated at 172 mA)

The 780 nm LED was used in a similar setup in the irradiation of Cy7 derivative **11** at the maximum power of 800 mA.

# 5. HPLC-based Investigation of (photo-)chemical Properties

**Stability and photorelease in cell culture medium (DMEM)**

HT-29 cancer cells were seeded into 96-well plates (clear polystyrene plates, Greiner Bio-One GmbH, Frickenhausen) with a concentration of 5 x 10^3^ cells/well contained in 100 µL of cell medium (DMEM). After 24 h, the medium was exchanged to fresh DMEM containing the desired compounds at a concentration of 100 µM (stock solutions of compounds in DMSO at 50-100 mM concentrations). Light-sensitive solutions were added to the cells in the dark. After the addition of the desired compounds, plates were directly irradiated with blue light (455 nm, 13 mW/cm^2^) with an in-house built LED-based irradiation device (*vide supra*) or kept entirely in the dark. After 72 h at 37 °C in the presence of 5% CO_2_, the supernatants were removed, wells were washed with 100 µL of PBS x 1 and the combined solutions were subjected to a C_18_ solid phase extraction (adapted from *Werz and Steinhilber*,^[1]^ conducted in the presence of red light) to remove water soluble compounds:

The aqueous solutions were applied to a C_18_ solid phase column preconditioned with 1 mL of methanol and 1 mL of water and then washed afterwards with 1 mL of water and 1 mL of 25% methanol. The organic compounds were then eluted with 300 µL of methanol and analyzed by HPLC.

Due to the polarity of the cytostatic payloads they were lost in the solid phase extraction and are not referenced in the following figures due to poor visibility.

**Stability and photorelease in 0.1 x PBS**

Stock solutions of the desired compounds were prepared at 50-100 mM concentrations. Compounds were then dissolved in 0.1 x PBS with 5-20 % DMSO as cosolvent at 25-100 µM concentrations with 50 µM uridine added as an internal standard (unless stated otherwise). Samples were irradiated as 100 µM aliquots in polystyrene 96-well plates (clear polystyrene plates, Greiner Bio-One GmbH, Frickenhausen) directly through the polystyrene cover of the plate. For compounds **1**, **2c**, **3a**, **3b**, **4a-4b**, **5a - 10** the previously mentioned in-house built LED light source was used (455 nm, 13 mW/cm^2^). Cy7 based compound **11** was irradiated with the previously mentioned in-house built LED light source (695 nm, 16 mW/cm^2^). Irradiations at 780 nm were conducted with a Thorlabs LED (M780L3, operated at 800 mA, 1600 mW, driven by Thorlabs LED driver DC4104 with DC4100-Hub attached) focussed with Thorlabs ACL2520-A Lens. Hydrolysis samples were kept in the dark for at least several days.

For the irradiation of compound **11** under the exclusion of oxygen two samples (0.5 mL) were prepared as described above and degassed extensively with argon. One sample was irradiated as described above at 780 nm while the other sample was kept in the dark as a control.

The desired samples were then analyzed with an HPLC system (1260 infinity LC system, Agilent Technologies) equipped with a quaternary pump, automatic liquid sampler, temperature-controlled column compartment and a diode array detector. A MultoKrom 100-5 C_18_ column (250 x 4.6 mm) from CS-Chromatographie Service GmbH was used for separation with a mixture of acetonitrile (B) and 100 mM NH_4_OAc-buffer in ultra-pure water (pH 7) as an eluent (concentration gradient between 5%-100% acetonitrile).

Two different gradients were used

Gradient I (used unless stated otherwise):

0 min 5 % B – 24 min 100 % B – 28 min 100 % B at a flow rate of 1.0 mL/min

Gradient II:

0 min 5 % B – 34 min 100 % B – 38 min 100 % B at a flow rate of 1.0 mL/min

Figure S6: a) HPLC-traces of **DEACM-OH** 72 h after incubation in the presence of HT-29 cells in DMEM (100 µM, 0.1% DMSO, 37 °C). Freshly prepared reference sample of **DEACM-OH** (yellow curve) is shown at the bottom. The sample kept in the dark (- hv) is shown in black, the irradiated sample is shown in red (+ hv, 455 nm) and shows no difference to the sample kept in the dark. This indicates a high stability of the used PPG in DMEM with no unexpected side reactions. b) HPLC-traces of the DMEM control (without phenol red) treated as described in the results section. One sample was kept in the dark (black curve) and one was irradiated (red curve). Several signals are visible which can be seen in every other sample incubated in the same way.

Figure S7: a) HPLC-traces of compound **1** 72 h after incubation in the presence of HT-29 cells in DMEM (100 µM, 0.1% DMSO, 37 °C). Freshly prepared reference samples are shown for compound **1** (grey curve) and **DEACM-OH** (yellow curve) at the bottom. The sample kept in the dark (- hv) is shown in black, the absence the of **DEACM-OH** signal is indicating a high stability in the dark. The irradiated sample is shown in red (+ hv, 455 nm) with an insignificant amount of **DEACM-OH** being visible which shows the poor photolytic behavior of compound **1**. b) HPLC-traces of compound **1** before (black curve), and after irradiation (455 nm, blue curves) in 0.1 x PBS (50 µM, 5% DMSO, uridine as internal standard added). A clear decrease in starting material is visible after irradiation, accompanied by the release of **DEACM-OH** and 6-mercaptopurine (**6MP**), although at low intensities. The hydrolytic stability was tested in the dark for 4 d (grey curve) with no release of **6MP** being detectable. The decrease of starting material **1** can be attributed to precipitation. c) Magnified HPLC-traces of compound **1** before (black curve), and after irradiation (455 nm, blue curves) in 0.1 x PBS (50 µM, 5% DMSO) to visualize a possible photolysis side product.

Figure S8: a) HPLC-traces of compound **2c** 72 h after incubation in the presence of HT-29 cells in DMEM (100 µM, 0.1% DMSO, 37 °C). Freshly prepared reference samples are shown for compound **2c** (grey curve) and **DEACM-OH** (yellow curve) at the bottom. The sample kept in the dark (- hv) is shown in black, the low intensity of the **DEACM-OH** signal is indicating a high stability in the dark. The irradiated sample is shown in red (+ hv, 455 nm), no starting material is remaining and the presence of **DEACM-OH** shows the fast photolysis of **2c** in DMEM. A second photolysis product, which might be attributed to a rearrangement reaction,^[2]^ is clearly visible here. b) HPLC-traces of compound **2c** before (black curve), and after irradiation (455 nm, blue curves) in 0.1 x PBS (50 µM, 5% DMSO, uridine as internal standard added). A clear decrease in starting material is visible after irradiation, accompanied by the release of **DEACM-OH**, floxuridine (**flox**) and a photolysis side product. The hydrolytic stability was tested in the dark for 2 d (grey curve) with no release of **flox** being detectable.

Figure S9: a) HPLC- traces of compound **3a** 72 h after incubation in the presence of HT-29 cells in DMEM (100 µM, 0.1% DMSO, 37 °C). Freshly prepared reference samples are shown for compound **3a** (grey curve) and **DEACM-OH** (yellow curve) at the bottom. The sample kept in the dark (- hv) is shown in black, the low intensity of the **DEACM-OH** signal is indicating a high stability in the dark. The irradiated sample is shown in red (+ hv, 455 nm), little starting material is remaining and the presence of **DEACM-OH** shows the fast photolysis of **3a** in DMEM. b) HPLC-traces of compound **3a** before (black curve), and after irradiation (455 nm, blue curves) in 0.1 x PBS (50 µM, 5% DMSO, uridine as internal standard added). A clear decrease in starting material is visible after irradiation, accompanied by the release of **DEACM-OH** and gemcitabine (**gem**). The hydrolytic stability was tested in the dark for 4 d (grey curve) with only small amounts of **gem** being detectable.

Figure S10: a) HPLC-traces of compound **3b** 72 h after incubation in the presence of HT-29 cells in DMEM (100 µM, 1% DMSO, 37 °C). Freshly prepared reference samples are shown for compound **3b** (grey curve) and **DEACM-OH** (yellow curve) at the bottom. The sample kept in the dark (- hv) is shown in black, the low intensity of the **DEACM-OH** signal is indicating a high stability in the dark. The irradiated sample is shown in red (+ hv, 455 nm), significantly less starting material is remaining and the presence of **DEACM-OH** shows the fast photolysis of **3b** in DMEM. b) HPLC-traces of compound **3b** before (black curve), and after irradiation (455 nm, blue curves) in 0.1 x PBS (50 µM, 5% DMSO). A clear decrease in starting material is visible after irradiation, accompanied by the release of **DEACM-OH** and cytarabine (**araC**). The hydrolytic stability was tested in the dark for 5 d (grey curve) with only small amounts of **araC** being detectable.

Figure S11: a) HPLC-traces of compound **4a** 72 h after incubation in the presence of HT-29 cells in DMEM (100 µM, 0.1% DMSO, 37 °C). Freshly prepared reference samples are shown for compound **4a** (grey curve) and **DEACM-OH** (yellow curve) at the bottom. The sample kept in the dark (- hv) is shown in black, the low intensity of the **DEACM-OH** signal is indicating a high stability in the dark. The irradiated sample is shown in red (+ hv, 455 nm), little starting material is remaining and the presence of **DEACM-OH** shows the fast photolysis of **4a** in DMEM. b) HPLC-traces of compound **4a** before (black curve), and after irradiation (455 nm, blue curves) in 0.1 x PBS (50 µM, 5% DMSO, uridine as internal standard added). A clear decrease in starting material is visible after irradiation, accompanied by the release of **DEACM-OH** and clofarabine (**clf**). The hydrolytic stability was tested in the dark for 3 d (grey curve) with no **clf** being detectable.

Figure S12: a) HPLC-traces (gradient II used) of compound **4b** 72 h after incubation in the presence of HT-29 cells in DMEM (100 µM, 0.1% DMSO, 37 °C). Freshly prepared reference samples are shown for compound **4b** (grey curve) and **DEACM-OH** (yellow curve) at the bottom. The sample kept in the dark (- hv) is shown in black, the low intensity of the **DEACM-OH** signal is indicating a high stability in the dark. The irradiated sample is shown in red (+ hv, 455 nm), significantly less starting material is remaining and the presence of **DEACM-OH** shows the fast photolysis of **4b** in DMEM. b) HPLC-traces (gradient II used) of compound **4b** before (black curve), and after irradiation (455 nm, blue curves) in 0.1 x PBS (50 µM, 5% DMSO, uridine as internal standard added). A clear decrease in starting material is visible after irradiation, accompanied by the release of **DEACM-OH** and cladribine (**cla**). The hydrolytic stability was tested in the dark for 1 d (grey curve) with no **cla** being detectable.

Figure S13: a) HPLC-traces (gradient II used) of compound **5a** 72 h after incubation in the presence of HT-29 cells in DMEM (100 µM, 0.1% DMSO, 37 °C). Freshly prepared reference samples are shown for compound **5a** (grey curve) and **DEACM-OH** (yellow curve) at the bottom. The sample kept in the dark (- hv) is shown in black, the high intensity of the **DEACM-OH** signal in addition to the low intensity of **5a** is indicating a low stability in DMEM. The irradiated sample is shown in red (+ hv, 455 nm), significantly less starting material is remaining and the high intensity of **DEACM-OH** shows the fast photolysis of **5** in DMEM. b) HPLC-traces (gradient I used) of compound **5a** before (black curve), and after irradiation (455 nm, blue curves) in 0.1 x PBS (50 µM, 5% DMSO, uridine as internal standard added). A clear decrease in starting material isn’t visible after irradiation due to similar retention times with the **DEACM-OH** signal. The release of floxuridine (**flox**) is clearly visible. c) HPLC- traces (gradient II used) of compound **5a** in 0.1 x PBS (50 µM, 5% DMSO, uridine as internal standard added) kept in the dark for several hours (black curve) or several days (grey curves). After 2 d only small amounts of **flox** are visible indicating a high hydrolytic stability.

Figure S14: a) HPLC-traces of compound **5b** 72 h after incubation in the presence of HT-29 cells in DMEM (100 µM, 0.1% DMSO, 37 °C). Freshly prepared reference samples are shown for compound **5b** (grey curve) and **DEACM-OH** (yellow curve) at the bottom. The sample kept in the dark (- hv) is shown in black, the high intensity of the **DEACM-OH** signal accompanied by the low intensity of **5b** is indicating a low stability in DMEM. The irradiated sample is shown in red (+ hv, 455 nm), no remaining starting material and the presence of **DEACM-OH** shows the photolysis of **5b** in DMEM. Additional side products are visible in this case. b) HPLC-traces of compound **5b** before (black curve), and after irradiation (455 nm, blue curves) in 0.1 x PBS (50 µM, 5% DMSO, uridine as internal standard added). A clear decrease in starting material is visible after irradiation, accompanied by the release of **DEACM-OH**, fludarabine (**araF**), and some unidentified side products. The hydrolytic stability was tested in the dark for 2 d (grey curve) with only small amounts of **araF** being detectable.

Figure S15: a) HPLC-traces of compound **3c** before (black curve), and after irradiation (455 nm, blue curves) in 0.1 x PBS (50 µM, 5% DMSO, uridine as internal standard added). The hydrolytic stability was tested in the dark for 1 h (grey curve). Due to fast hydrolysis and similar retention times between decitabine and Uridine no clear signals could be assigned for neither compound 3c nor decitabine (dec). Only **DEACM-OH** was clearly visible in all cases. The fast hydrolytic behavior is in agreement with previous reports.^[3]^ b) HPLC-traces of compound **6** before (black curve), and after irradiation (455 nm, blue curves) in 0.1 x PBS (50 µM, 5% DMSO, uridine as internal standard added). A slow decrease in starting material is visible after irradiation, accompanied by the appearance of floxuridine (**flox**) and several other signals indicating an unclean photoreaction. c) HPLC-traces of compound **7** before (black curve), and after irradiation (455 nm, blue curves) in 0.1 x PBS (50 µM, 5% DMSO, uridine as internal standard added). A slow decrease in starting material is visible after irradiation, accompanied by the release of small amounts of gemcitabine (**gem**). The hydrolytic stability was tested in the dark for 4 d (grey curve) with small amounts of **gem** being detectable.

Figure S16: a) HPLC-traces of compound **2b** before (black curve), and after irradiation (455 nm, blue curves) in 0.1 x PBS (50 µM, 5% DMSO, uridine as internal standard added). A clear decrease in starting material is visible after irradiation, accompanied by the release of **DEACM-OH**, tegafur (**teg**) and an unidentified side product. The hydrolytic stability was tested in the dark for 2 d (grey curve) with no **teg** being detectable. b) HPLC-traces of compound **2a** before (black curve), and after irradiation (455 nm, blue curves) in 0.1 x PBS (50 µM, 5% DMSO). A reference sample of **DEACM-OH** is shown at the bottom (yellow trace, 50 µM, 5% DMSO, uridine as internal standard added). A clear decrease in starting material is visible after irradiation, accompanied by the release of **DEACM-OH**, 5-fluorouracil (**5FU**) and several unidentified side products indicating an unclean photoreaction.^[2]^ c) HPLC-traces of compound **2a** before (black curve), and after irradiation (455 nm, blue curves) in 0.1 x PBS (50 µM, 5% DMSO, uridine as internal standard added). A clear decrease in starting material is visible after irradiation, accompanied by the release of **DEACM-OH** and several unidentified side products indicating an unclean photoreaction.^[2]^ The release of **5FU** can’t be seen in this case due to a similar retention with the uridine standard. The hydrolytic stability was tested in the dark for 2 d (grey curve) with no **DEACM-OH** being detectable. The low overall signal intensity of **2b** (in comparison to compounds **2a** and **2c**) can be attributed to the poor solubility of compound **2b**.

Figure S17: a) HPLC-traces of compound **8** before (black curve), and after irradiation (455 nm, blue curves) in 0.1 x PBS (25 µM, 10% DMSO, uridine as internal standard added). A clear decrease in starting material is visible after extensive irradiation which isn’t accompanied by the release of **DEACM-OH** or the desired methotrexate (**MTX**). Several unidentified side products are visible instead indicating a decomposition. The hydrolytic stability was tested in the dark for 2 d (grey curve) with no **MTX** being detectable. b) HPLC-traces of compound **9** before (black curve), and after irradiation (455 nm, blue curves) in 0.1 x PBS (25 µM, 10% DMSO, uridine as internal standard added). A clear decrease in starting material is visible after extensive irradiation which is not accompanied by the release of the desired raltitrexed (**RTX**). Only small amounts of **DEACM-OH** are visible accompanied by the appearance of several unidentified side products indicating a decomposition. The hydrolytic stability was tested in the dark for 2 d (grey curve) with no **RTX** being detectable. c) HPLC-traces of compound **10** before (black curve), and after irradiation (455 nm, blue curves) in 0.1 x PBS (25 µM, 10% DMSO, uridine as internal standard added). A clear decrease in starting material is visible after extensive irradiation which is accompanied by the appearance of small amounts of pemetrexed (**PMX**) and **DEACM-OH**. Several unidentified side products are visible indicating a decomposition. The hydrolytic stability was tested in the dark for 2 d (grey curve) with no **PMX** being detectable.

Figure S18: a) HPLC-traces of compound **11** before (black curve), and after irradiation (695 nm, green curves) in 0.1 x PBS (100 µM, 5% DMSO, uridine as internal standard added). A clear decrease in starting material is visible after irradiation, accompanied by the release of an unidentified side product and gemcitabine (**GEM**). The hydrolytic stability was tested in the dark for 2.5 d (grey curve) with **GEM** being detectable indicating a slow hydrolysis of compound **11**. b) HPLC-traces of compound **11** before (black curve), and after irradiation (780 nm, green curves) in 0.1 x PBS (100 µM, 5% DMSO, uridine as internal standard added). Absorption is shown at 254 nm and at 700 nm (upper and lower 2 traces). Similar to the irradiation at 695 nm, a clear decrease in starting material is visible after irradiation. This is accompanied by the release of an unidentified side product and gemcitabine (**GEM**). c) HPLC-traces of compound **11** before (black curve), and after irradiation (780 nm, green curves) in degassed 0.1 x PBS (100 µM, 5% DMSO, uridine as internal standard added). Absorption is shown at 254 nm and at 700 nm (upper and lower 2 traces). As in previous cases, a decrease of the starting material accompanied by the release of **GEM** is clearly visible.

## 5.2 Quantum Yield Determination

Quantum Yields of the synthesized compounds were approximated from the previously described 96-well plate HPLC-irradiation experiments.

Figure S19: Amount of starting material (shown in %) of: a) **6MP**-derivative **1**, **Flox**-derivative **6** and antifolate-derivatives **8**-**10** at different irradiation times. b) compounds **2c**, **2a**, **3b**, **4a**, **4b**, **5a** and **5b** at different irradiation times. Due to overlapping retention times, in the cases of compounds **5a** and **4b** the disappearance of the starting material was calculated by the appearance of the respective payloads in the corresponding traces. c) Cy7-derivative **11** in comparison to the respective DEACM-derivative **3a** at different irradiation times. The disappearance of starting material was determined by the previously described HPLC experiments. Decay curves were fitted by exponential functions; irradiations were conducted with the previously mentioned in-house built light sources (455 nm or 695 nm for compound **11**). Compounds **7** and **2b** did not show a simple decay curve due to precipitation and thus were not included.

The molar photon flux $n_{photons}$ of the irradiation sources was estimated using the previously determined power density to calculate the respective spectral radiant power $P_{\lambda}$ experienced by the irradiated wells. The photon flux was then calculated by the following equation with $E_{\lambda}$being the spectral Energy:

$$n_{photons}=\frac{P_{\lambda}}{E_{\lambda}}= \frac{P_{\lambda}}{h\frac{c}{\lambda}}$$

The quantum yield $\Phi$ was then calculated by using the following equation as previously described by us.^[4]^

$$\Phi= \frac{\left[ c \right]\cdot V\cdot\Delta c}{n_{photons}\cdot{(1-10}^{-A})}$$

$V$ and $\left[ c \right]$ are the volume and the concentration of the irradiated solution and $\Delta c$ is the change in concentration at the initial rate of the photoreaction. $\Delta c$ was determined by HPLC of solutions irradiated for different time intervals as described previously.^[4]^ The corresponding disappearances of starting materials are shown in Supporting Figure S19.

$A$ is the absorbance at the irradiation wavelength $\lambda$ calculated by using the Beer-Lambert law. The corresponding path length was calculated from the volume $V$ of the irradiated solution and the diameter $d$ of the irradiated wells ($d$ = 6.39 mm according to the manufacturer).

# 6. HPLC-MS Investigation of the Photorelease

Coumarin derivatives **1**, **2c**, **3a**, **3b**, **4a**, **4b**, **5a**, **5b** and Cy7 derivative **11** were solved in 0.1 x PBS (5% DMSO) at a concentration of 50 µM. Samples were irradiated as 100 µM aliquots in polystyrene 96-well plates (clear polystyrene plates, Greiner Bio-One GmbH, Frickenhausen) directly through the polystyrene cover of the plate. For compounds **1**, **2c**, **3a**, **3b**, **4a**, **4b**, **5a**, **5b** an in-house built LED light source (*vide supra*) was used (455 nm, 13 mW/cm^2^) for 3 minutes. Cy7 based compound **11** was irradiated for 2 h with an in-house built LED light source (*vide supra*, 695 nm, 16 mW/cm^2^). Control samples were kept in the dark. The desired samples were then analyzed with an HPLC system (Agilent 1260 Infinity II device with a G7117C 1260 HS diode array detector) coupled to a LC/MSD device (G6125B, ESI pos./neg. 100-1000). An ACE UltraCore Super C18 (Aventor, 3 x 150 mm) reversed phase column was used with 0.1% formic acid in water (A) and 0.1% formic acid in acetonitrile (B) as a mobile phase. A gradient of: 0 min: 5% B – 2 min: 80% B – 5 min: 95% B – 7 min: 95% B at a flow rate of 0.6 mL/min was used.

Figure S20: HPLC-MS investigation (positive mode) of the uncaging behaviour of 6-mercaptopurine derivative **1**. The chromatograms at 254 nm are shown with the detected mass spectra added at the corresponding retention times. The sample irradiated at 455 nm is shown in blue (+ hv) and the sample kept in the dark is shown in black (- hv). The desired payload was not detectable (retention time of 6-mercaptopurine: 1.90 min).

Figure S21: HPLC-MS investigation (positive mode) of the uncaging behaviour of floxuridine derivative **2c**. The chromatograms at 254 nm are shown with the detected mass spectra added at the corresponding retention times. The sample irradiated at 455 nm is shown in blue (+ hv) and the sample kept in the dark is shown in black (- hv). The released payload is clearly visible at a retention time of 2.2 min in the irradiated sample. A mass spectrum of this signal was not obtainable in positive mode.

Figure S22: HPLC-MS investigation (negative mode) of the uncaging behaviour of floxuridine derivative **2c**. The chromatograms at 254 nm are shown with the detected mass spectra added at the corresponding retention times. The sample irradiated at 455 nm is shown in blue (+ hv) and the sample kept in the dark is shown in black (- hv). The released payload is clearly visible at a retention time of 2.27 min in the irradiated sample.

Figure S23: HPLC-MS investigation (positive mode) of the uncaging behaviour of gemcitabine derivative **3a**. The chromatograms at 254 nm are shown with the detected mass spectra added at the corresponding retention times. The sample irradiated at 455 nm is shown in blue (+ hv) and the sample kept in the dark is shown in black (- hv). The released payload is clearly visible at a retention time of 1.49 min in the irradiated sample.

Figure S24: HPLC-MS investigation (positive mode) of the uncaging behaviour of cytarabine derivative **3b**. The chromatograms at 254 nm are shown with the detected mass spectra added at the corresponding retention times. The sample irradiated at 455 nm is shown in blue (+ hv) and the sample kept in the dark is shown in black (- hv). The released payload is clearly visible at a retention time of 1.12 min in the irradiated sample.

Figure S25: HPLC-MS investigation (positive mode) of uncaging behaviour of fludarabine derivative **5b**. The chromatograms at 254 nm are shown with the detected mass spectra added at the corresponding retention times. The sample irradiated at 455 nm is shown in blue (+ hv) and the sample kept in the dark is shown in black (- hv). The released payload is clearly visible at a retention time of 2.66 min in the irradiated sample.

Figure S26: HPLC-MS investigation (positive mode) of uncaging behaviour of clofarabine derivative **4a**. The chromatograms at 254 nm are shown with the detected mass spectra added at the corresponding retention times. The sample irradiated at 455 nm is shown in blue (+ hv) and the sample kept in the dark is shown in black (- hv). The released payload is clearly visible at a retention time of 2.90 min in the irradiated sample.

Figure S27: HPLC-MS investigation (positive mode) of uncaging behaviour of cladribine derivative **4b**. The chromatograms at 254 nm are shown with the detected mass spectra added at the corresponding retention times. The sample irradiated at 455 nm is shown in blue (+ hv) and the sample kept in the dark is shown in black (- hv). The released payload is clearly visible at a retention time of 2.81 min in the irradiated sample.

Figure S28: HPLC-MS investigation (positive mode) of uncaging behaviour of floxuridine derivative **5a**. The chromatograms at 254 nm are shown with the detected mass spectra added at the corresponding retention times. The sample irradiated at 455 nm is shown in blue (+ hv) and the sample kept in the dark is shown in black (- hv). The released payload is clearly visible at a retention time of 2.2 min in the irradiated sample. A mass spectrum of this signal was not obtainable in positive mode.

Figure S29: HPLC-MS investigation (negative mode) of uncaging behaviour of floxuridine derivative **5a**. The chromatograms at 254 nm are shown with the detected mass spectra added at the corresponding retention times. The sample irradiated at 455 nm is shown in blue (+ hv) and the sample kept in the dark is shown in black (- hv). The released payload is clearly visible at a retention time of 2.30 min in the irradiated sample.

Figure S30: HPLC-MS investigation (positive mode) of uncaging behaviour of gemcitabine derivative **11**. The chromatograms at 254 nm are shown with the detected mass spectra added at the corresponding retention times. The sample irradiated at 695 nm is shown in green (+ hv) and the sample kept in the dark is shown in black (- hv). The released payload is clearly visible at a retention time of 1.50 min in the irradiated sample. Mass fragments consistent with the oxidative cleavage of the Cy7-scaffold^[5]^ were also detectable.

# 7. Synthesis of the Light activatable Cytostatics

## 7.1 Synthetic Overview

Figure S31: Synthesis of 6-mercaptopurine derivative **1**, tegafur and 5-fluorouracil derivatives **2a** and **2b**. a) **DEACM-Br**, Cs_2_CO_3_, in CH_2_Cl_2_/MeCN 1:1 at rt, 12%; b) 1,2,4-Triazole, POCl_3_, NEt_3_ in MeCN at 0 °C, 98%; c) **DEACM-OH**, DBU in MeCN at rt, 50%; d) TsOH in THF, 35%. Coumarin starting materials used in this studies were **DEACM-Br**,^[6]^ **DEACM-CDI**,^[7]^ **DEACM-PFPC**,^[3]^ **DEACM-OH**^[8]^ and **DEACM-Bn-OH**^[2]^.

Figure S32: Synthesis of floxuridine derivative **2c**, **5a** and **6**. a) TBDMSCl, imidazole in DMF quant.; b) 1,2,4-Triazole, POCl_3_, NEt_3_ in MeCN at 0 °C, 82%; c) **DEACM-OH**, DBU in MeCN at rt, 56% for **2c**; **DEACM-Bn-OH**, DBU in CH_2_Cl_2_, 51% for **6**; d) TBAF in THF/AcOH, 72% for **2c**, 56% for **6**; e) **DEACM-PFPC**, DMAP in DMF 21%.

Figure S33: Synthesis of pyrimidine derivatives **3a-c** and **7**. a) TBDMSCl, imidazole in DMF 65% for **3a**, 90% for **3c**; b) **DEACM-CDI**, LiHMDS in THF -78 °C to rt, 40 % for **3a**, 40% for **3c**, 73% for **7.1**; c) TBAF in THF/AcOH, 48% for **3a**, 40% for **3c**, 50% for **7**; d) **DEACM-PFPC** in pyridine at rt, 9% for **3a**, 34% for **3b**.

Figure S34: Synthesis of purine derivatives **4a** and **4b**. a) TBDMSCl, imidazole in DMF 85% for **4a**, 89% for **4b**; b) **DEACM-CDI**, LiHMDS in THF -78 °C to rt, 93% for **4a**, 90% for **4b**; c) TBAF in THF/AcOH 59% for **4a**, 18% for **4b**.

Figure S35: a) TBDMSCl, imidazole in DMF 52%; b) 1. (**5b.2**): CDI in CH_2_Cl_2_ at 50°C (MW) 2. (**5b.3**): **DEACM-OH**, DMAP in CH_2_Cl_2_ at 50°C (MW) 40 % over both steps; c) TBAF in THF/AcOH, 80%.

Figure S36: Synthesis of antifolate derivatives **8-10**. a) **DEACM-OH**, DMAP, EDC-HCl in CH_2_Cl_2_ 15% for **8**, 76% for **9**, 27% for **10**.

Figure S37: Synthesis of Cy7-substituted gemcitabine **11**, a) NP = 4-nitrophenyl, LiHMDS in THF, -78 °C to rt, 42%; b) 2,4-dinitrophenyltriflate in MeCN, rt, crude; c) 1,2,3,3-tetramethy-3*H*-indol-1-ium iodide, AcOK in MeCN, rt, 5.8%.

## 7.2. Synthetic Materials and Methods

Antimetabolite cytostatics were purchased from BLDpharm, TCI or ABCR. Chemical reagents and solvents were purchased from Doug Discovery, TCI, ABCR, Carl Roth, VWR, and Sigma Aldrich and used without further purification. Chemical synthesis was conducted in dry solvents (Arcos Organics, kept over molecular sieves) under exclusion of water in an argon atmosphere unless stated otherwise. Microwave heating was performed in sealed vessels in a Biotage Initiator microwave reactor (max. microwave power 400 W, frequency 2450 MHz, 220-240 V). Reactions were monitored by TLC (silica gel 60 – coated aluminum sheet, UV254 indicator, Macherey-Nagel) and the crude products were purified by column chromatography with silica gel (silica gel 60, Macherey-Nagel). Flash column chromatography was conducted with a puriFlash® XS420 System (*Interchim*).

Purity of final compounds was then evaluated by NMR-spectroscopy and HPLC-chromatography. Further characterization was performed by mass spectrometry. NMR spectra were recorded in deuterated dimethylsulfoxide (DMSO-*d*_6_), chloroform (CDCl_3_) or methanol (Methanol-*d*_4_) purchased from euroisotop. Measurements were conducted with Bruker Avance III HD 400 MHz, Bruker Avance III HD 500 MHz (with BBO-Prodigy probe head) or Bruker DRX 600 MHz spectrometers. All ^1^H or ^13^C shifts are reported in ppm and were measured with the residual solvent signal (DMSO-*d*_6_: 2.50 ppm (^1^H) / 39.52 ppm (^13^C), CDCl_3_: 7.26 ppm (^1^H) / 77.16 ppm (^13^C), Methanol-*d*_4_: 3.31 ppm (^1^H) / 49.00 ppm (^13^C)) acting as an internal reference. The abbreviations used were s = singlet, d = doublet, t = triplet, q = quartet, m = multiplet, br = broad signal. The measured coupling constants are reported in Hertz (Hz). In the case of molecules containing fluorine atoms the ^19^F - ^13^C coupling constants were reported with the respective ^13^C-NMR spectra. The protons of the sugar moieties were assigned by ^1^H-COSY experiments, ^1^H -^13^C HSQC, HMBC and H2BC experiments were used to confirm chemical structures. ^13^C-^19^F coupled carbon atoms were assigned ^19^F decoupled ^13^C-NMR spectra and by ^1^H-^13^C HSQC and HMBC experiments. The assigned ^13^C chemical shifts and ^13^C-^19^F coupling constants were in good agreement with reported ^13^C-spectra for clofarabine,^[9,10]^ 5-fluorouracil,^[11,12]^ gemcitabine^[10,13,14]^ and fludarabine.^[15,16]^

Low resolution electrospray ionization mass spectra were measured on a Themo Fisher Surveyor MSQ device. High-resolution mass spectra were obtained from a Thermo Fisher LTQ Orbitrap XL (MALDI-HRMS, 5 decimal places) device, Thermo Fisher Q Exactive Plus device (ESI-HRMS, 4 decimal places, used for compounds **3c.1**, **3c.2** and **4b**) or a Bruker MicroTof-QII device (ESI-HRMS, 4 decimal places).

## 7.3  General Synthetic Procedures

## 7.3.1 General Procedure A for TBDMS Protection

The cytostatic was dissolved in dry dimethylformamide (DMF) and imidazole and *tert*-butyldimethysilyl chloride (TBDMSCl) were added at rt. The reaction was stirred at ambient temperature until TLC showed a full conversion of the starting material. The reaction mixture was diluted with CH_2_Cl_2_, washed with saturated NaHCO_3_ (sat.) and distilled H_2_O, dried over Na_2_SO_4_, filtered and the solvent was removed under reduced pressure. The crude product was purified by column chromatography to yield the desired product.

## 7.3.2 General Procedure B for Carbamate Formation

A Schlenk flask was charged with TBDMS protected cytostatic (1 equiv.) and three vacuum-argon cycles were conducted. Dry THF was added, and it was cooled to −78 °C. Lithium bis(trimethylsilyl)amide (LiHMDS, 1 mol/L solution in THF) was added under argon atmosphere, the reaction mixture was stirred for 5 min and was allowed to reach ambient temperature over 30 min. **DEACM-CDI** was dissolved in dry THF (usually 2 mL), added dropwise to the reaction and it was stirred for one hour at ambient temperature. The reaction was monitored by TLC with *c*-Hex/EtOAc mixtures. All volatiles were removed under reduced pressure and the crude product was purified by column chromatography with 1% NEt_3_ added to yield the desired product.

## 7.3.3 General Procedure C for TBDMS Deprotection

The TBDMS protected product was dissolved in THF/AcOH (4:1), the reaction was stirred at ambient temperature and tetra-*n*-butylammonium fluoride (TBAF) was added in aliquots (usually 2-3 eq. over several hours) until TLC indicated a full conversion of the starting material. It was diluted with EtOAc and saturated NaHCO_3_ solution was added. The reaction mixture was transferred into a separation funnel and washed with saturated NaHCO_3_ solution, four times with saturated NH_4_Cl solution and once with saturated NaCl solution. The organic layer was dried over Na_2_SO_4_, filtered and the solvent was removed under reduced pressure. The crude product was purified by column chromatography (CH_2_Cl_2_/MeOH 0-10%) to yield the photocaged cytostatic.

## 7.3.4 General Procedure D for Steglich Esterification

The desired antifolate, **DEACM-OH**, 4-dimethylaminopyridine (DMAP) and *N*-ethyl-*N*’-(3-dimethylaminopropyl)carbodiimide hydrochloride (EDC-HCl) were suspended in dry CH_2_Cl_2_. The mixture was stirred overnight at ambient temperature, and the solvent was removed under reduced pressure. The crude product was purified by column chromatography (CH_2_Cl_2_/MeOH).

## 7.4 Chemical Synthesis

## 7.4.1 4-(((9*H*-Purin-6-yl)thio)methyl)-7-(diethylamino)-2*H*-chromen-2-one (6MP-DEACM, 1)

Mercaptopurine (100 mg, 657 µmol) was combined with **DEACM-Br** (306 mg, 0.986 mmol,) and Cs_2_CO_3_ (321 mg, 0.986 mmol) in CH_2_Cl_2_/MeCN (1:1) and stirred overnight at ambient temperature. The reaction was diluted with CH_2_Cl_2_ and transferred into a separation funnel, washed with saturated NaHCO_3_ solution and water, dried over Na_2_SO_4_, filtered and all volatiles were removed. Column chromatography (CH_2_Cl_2_/MeOH 0-10%) was carried out to obtain **1** (25 mg, 0.066 mmol, 12%). A double substituted product could also be observed.

**^1^H-NMR** (500 MHz, DMSO-*d*_6_) δ 13.58 (br s, 1H, N-*H*), 8.76 (s, 1H, Ar-*H*), 8.47 (s, 1H, Ar-*H*), 7.68 (d, *J* = 9.0 Hz, 1H, DEACM-*H*5), 6.71 (dd, *J* = 9.1, 2.6 Hz, 1H, DEACM-*H*6), 6.53 (d, *J* = 2.5 Hz, 1H, DEACM-*H*8), 6.17 (s, 1H, DEACM-*H*3), 4.79 (s, 2H, DEACM-C*H*_2_), 3.42 (q, *J* = 7.0 Hz, 4H, NC*H*_2_CH_3_), 1.11 (t, *J* = 7.0 Hz, 6H, NCH_2_C*H*_3_).

**^13^C-NMR** (126 MHz, DMSO-*d*_6_) δ 160.65, 156.33, 156.01, 152.30, 151.50, 150.52, 149.73, 143.75, 129.63, 126.04, 108.77, 107.76, 106.55, 96.92, 43.99, 27.38, 12.31.

**HRMS:** *m/z* calculated for C_19_H_20_N_5_O_2_S^+^ [M-H^+^] 382.1332, found 382.1340 [M-H^+^].

**TLC:** R*_f_* = 0.2 (CH_2_Cl_2_/MeOH 9:1).

## 7.4.2 5-Fluoro-1-(tetrahydrofuran-2-yl)-4-(1*H*-1,2,4-triazol-1-yl)pyrimidin-2(1*H*)-one (Tegafur-Triazole, 2b.1)

To a suspension of 1,2,4-1*H*-triazole (1.38 g, 20.0 mmol) in 12 mL of dry MeCN POCl_3_ (0.46 mL, 5.0 mmol) was added dropwise at 0 °C. Triethylamine (2.8 mL, 20 mmol) was added over 5 minutes and the resulting solution was stirred for 10 minutes. Tegafur (0.200 g, 0.999 mmol) was dissolved in 12 mL of dry MeCN and added dropwise over an hour to the solution at 0 °C. The resulting mixture was stirred overnight at ambient temperature. 10 mL of saturated NaHCO_3_ solution was added, the MeCN was removed under reduced pressure and the resulting aqueous phase was extracted three times with CH_2_Cl_2_. The organic phase was dried over Na_2_SO_4_ and concentrated under reduced pressure. The desired compound was obtained as an orange solid and used without further purification (0.239 g, 0.949 mmol, 95%).

**^1^H NMR** (500 MHz, DMSO-*d*_6_) δ 9.37 (s, 1H, Triazole-*H*), 8.60 (d, *J_H-F_* = 6.7 Hz, 1H, Ar-*H*), 8.43 (s, 1H, Triazole-*H*), 5.99 – 5.89 (m, 1H, C*H*CH_2_CH_2_CH_2_), 4.54 – 4.36 (m, 1H, CHCH_2_CH_2_C*H*_2_), 4.02 – 3.86 (m, 1H, CHCH_2_CH_2_C*H*_2_), 2.40 – 2.30 (m, 1H, CHC*H*_2_CH_2_CH_2_), 2.15 – 2.05 (m, 1H, CHC*H*_2_CH_2_CH_2_), 2.00 – 1.91 (m, 1H, CHCH_2_C*H*_2_CH_2_), 1.89 – 1.79 (m, 1H, CHCH_2_C*H*_2_CH_2_).

**^13^C NMR** (126 MHz, DMSO-*d_6_*) δ 154.18, 154.15, 151.24, 148.38 (d, *J*_C-F_ = 9.3 Hz), 145.53, 145.50, 135.98 (d, *J*_C-F_ = 247 Hz), 135.59 (d, *J*_C-F_ = 33.8 Hz), 89.03, 70.43, 32.28, 22.63.

**^19^F-NMR** (471 MHz, DMSO-*d*_6_) δ -160.06.

**HRMS:** *m/z* calculated for C_10_H_11_FN_5_O_2_^+^ [M-H^+^] 252.0891, found 252.0899 [M-H^+^].

**TLC:** R*_f_* = 0.01 (EtOAc/*c*-Hex 1:1).

## 7.4.3 4-((7-(Diethylamino)-2-oxo-2*H*-chromen-4-yl)methoxy)-5-fluoro-1-(tetrahydrofuran-2-yl)pyrimidin-2(1*H*)-one (DEACM-tegafur, 2b)

To a solution prepared of tegafur-derivative **2b.1** (0.245 g, 0.975 mmol) and **DEACM-OH** (0.241 g, 0.975 mmol) in 5 mL dry MeCN, 1,8-diazabicyclo[5.4.0]undec-7-ene (0.17 mL, 1.2 mmol) was added. The resulting solution was stirred overnight at ambient temperature, afterwards the solvent was removed under reduced pressure. The residue was purified by column chromatography (EtOAc/CH_2_Cl_2_/*c*-Hex 2:1:1, silica deactivated with 1% NEt_3_). The desired product was obtained as an orange solid (0.185 g, 0.431 mmol, 44%).

**^1^H-NMR** (500 MHz, DMSO-*d*_6_) δ 8.21 (d, *J_H-F_* = 6.2 Hz, 1H, Ar-*H*), 7.50 (d, *J* = 9.0 Hz, 1H, DEACM-*H*5), 6.72 (dd, *J* = 9.1, 2.6 Hz, 1H, DEACM-*H*6), 6.57 (d, *J* = 2.5 Hz, 1H, DEACM-*H*8), 6.02 (s, 1H, DEACM-*H*3), 5.93 – 5.86 (m, 1H, C*H*CH_2_CH_2_CH_2_), 5.61 (s, 2H, DEACM-C*H*_2_), 4.38 – 4.29 (m, 1H, CHCH_2_CH_2_C*H*_2_), 3.91 – 3.81 (m, 1H, CHCH_2_CH_2_C*H*_2_), 3.44 (q, *J* = 7.0 Hz, 4H, NC*H*_2_CH_3_), 2.34 – 2.22 (m, 1H, CHC*H*_2_CH_2_CH_2_), 2.05 – 1.96 (m, 1H, CHC*H*_2_CH_2_CH_2_), 1.96 – 1.81 (m, 2H, CHCH_2_C*H*_2_CH_2_), 1.12 (t, *J* = 7.0 Hz, 6H, NCH_2_C*H*_3_).

**^13^C-NMR** (126 MHz, DMSO-*d*_6_) δ 160.54, 160.48 (d, *J*_C-F_ = 13.2 Hz), 155.86, 152.01, 150.54, 149.77, 135.47 (d, *J*_C-F_ = 242 Hz), 129.81 (d, *J*_C-F_ = 31.0 Hz), 125.57, 108.84, 105.58, 105.22, 96.89, 87.88, 69.86, 63.98, 43.99, 32.21, 22.97, 12.29.

**^19^F-NMR** (471 MHz, DMSO-*d*_6_) δ -170.97.

**HRMS:** *m/z* calculated for C_22_H_25_FN_3_O_5_^+^ [M-H^+^] 430.17728, found 430.17708 [M-H^+^].

**TLC:** R*_f_* = 0.26 (EtOAc/CH_2_Cl_2_/*c*-Hex 2:1:1).

## 7.4.4 4-((7-(Diethylamino)-2-oxo-2*H*-chromen-4-yl)methoxy)-5-fluoropyrimidin-2(1*H*)-one (DEACM-5FU, 2a)

To a suspension of **DEACM-tegafur** (**2b**) (0.051 g, 0.12 mmol) in 5 mL dry THF *p*-toluenesulfonic acid (0.035 g, 0.20 mmol) was added. After stirring for several hours at ambient temperature, the solvent was removed under reduced pressure. The resulting residue was purified two times by column chromatography (1.: Acetone, 2.: CH_2_Cl_2_/MeOH 10:1) to yield the desired compound as a yellow powder (0.015 g, 0.03 mmol, 35 %).

**^1^H-NMR** (400 MHz, DMSO-*d*_6_) δ 11.42 (s, 1H, N-*H*), 8.11 (d, *J* = 5.5 Hz, 1H, Ar-*H*), 7.50 (d, *J* = 9.0 Hz, 1H, DEACM-*H*5), 6.72 (dd, *J* = 9.0, 2.6 Hz, 1H, DEACM-*H*6), 6.57 (d, *J* = 2.6 Hz, 1H, DEACM-*H*8), 6.01 (d, *J* = 1.2 Hz, 1H, DEACM-*H*3), 5.60 (d, *J* = 1.2 Hz, 2H, DEACM-C*H*_2_), 3.44 (q, *J* = 7.0 Hz, 4H, NC*H*_2_CH_3_), 1.12 (t, *J* = 7.0 Hz, 6H, NCH_2_C*H*_3_).

**^13^C-NMR** (126 MHz, DMSO-*d*_6_) δ 160.99 (d, *J*_C-F_ = 12.5 Hz), 160.56, 155.87, 153.94, 150.55, 149.91, 135.55 (d, *J*_C-F_ = 240 Hz), 131.57 (d, *J*_C-F_ = 38.3 Hz), 125.57, 108.85, 105.51, 105.24, 96.89, 63.65, 43.99, 12.29.

**^19^F-NMR** (471 MHz, DMSO-*d*_6_) δ -173.31.

**HRMS:** *m/z* calculated for C_18_H_19_FN_3_O_4_^+^ [M-H^+^] 360.13541, found 360.13541 [M-H^+^].

**TLC:** R*_f_* = 0.52 (Acetone), 0.57 (CH_2_Cl_2_/MeOH 10:1).

## 7.4.5 1‑((2*R*,4*S*,5*R*)‑4‑((*tert*‑Butyldimethylsilyl)oxy)‑5‑(((*tert*‑butyldimethylsilyl)oxy)methyl)tetrahydrofuran-2-yl)-5-fluoropyrimidine-2,4(1*H*,*3H*)-dione (TBDMS-Floxuridine)

Following the general procedure A with floxuridine (1.90 g, 7.73 mmol), imidazole (1.604 g, 23.56 mmol) and TBDMSCl (2.700 g, 17.91 mmol) in 15 mL dry DMF. The mixture was stirred for two days at ambient temperature. In this case saturated NaHCO_3_ solution and water were added and the solution was extracted with EtOAc. The organic phase was washed one time with water and two times with saturated NaCl solution. The solvent was removed under reduced pressure and the desired compound was obtained as a colorless gum (3.67 g, 7.73 mmol, quant). The product´s analytics corresponds to known literature.^[17]^

## 7.4.6 1‑((2*R*,4*S*,5*R*)‑4‑((*tert*‑Butyldimethylsilyl)oxy)‑5‑(((*tert*‑butyldimethylsilyl)oxy)methyl)tetrahydrofuran-2-yl)-5-fluoro-4-(1*H*-1,2,4-triazol-1-yl)pyrimidin-2(1*H*)-one (Triazoyl-TBDMS-Floxuridine, 2c.1)

To a suspension of 1,2,4-1*H*-triazole (1.50 g, 21.7 mmol) in 15 mL of dry MeCN POCl_3_ (0.50 mL, 5.4 mmol) was added dropwise at 0 °C. Triethylamine (3.0 mL, 22 mmol) was added over 15 minutes and **TBDMS-floxuridine** (0.515 g, 1.08 mmol) dissolved in 15 mL of dry MeCN was added dropwise to the solution at 0 °C. The resulting mixture was stirred overnight at ambient temperature. Saturated NaHCO_3_ solution, NaCl solution, water, CH_2_Cl_2_ were added and the aqueous phase was extracted seven times with CH_2_Cl_2_. The organic phase was dried over Na_2_SO_4_ and the desired compound was obtained as a yellow solid (0.467 g, 0.893 mmol, 82 %). The product´s analytics corresponds to known literature.^[18]^

## 7.4.7 1‑((2*R*,4*S*,5*R*)‑4‑((*tert*‑Butyldimethylsilyl)oxy)‑5‑(((*tert*‑butyldimethylsilyl)oxy)methyl)tetrahydrofuran-2-yl)-4-((7-(diethylamino)-2-oxo-2*H*-chromen-4-yl)methoxy)-5-fluoropyrimidin-2(1*H*)-one (TBDMS-DEACM-Floxuridine, 2c.2)

Floxuridine derivative **2c.1** (0.510 g, 0.970 mmol) was dissolved in dry MeCN with **DEACM-OH** (0.240 g, 0.970 mmol), 1,8-diazabicyclo[5.4.0]undec-7-ene (0.17 mL, 1.2 mmol) was added and the mixture was stirred for two days at ambient temperature. The solvent was removed under reduced pressure and the residue was purified by column chromatography (EtOAc/*c*-Hex 1:1). The desired compound was obtained as an orange solid (0.382 g, 0.543 mmol, 56 %).

**^1^H-NMR** (500 MHz, DMSO-*d*_6_) δ 8.29 (d, *J* = 6.2 Hz, 1H, Ar-*H*), 7.50 (d, *J* = 9.0 Hz, 1H, DEACM-*H*5), 6.71 (dd, *J* = 9.2, 2.6 Hz, 1H, DEACM-*H*6), 6.57 (d, *J* = 2.6 Hz, 1H, DEACM-*H*8), 6.08 – 6.03 (m, 1H, 1‘-*H*), 6.03 (s, 1H, DEACM-*H*3), 5.64 – 5.59 (m, 2H, DEACM-C*H*_2_), 4.41 – 4.34 (m, 1H, 3‘-*H*), 3.91 – 3.84 (m, 2H, 4‘-*H* + 5‘-*H*_a_), 3.78 – 3.70 (m, 1H, 5‘-*H*_b_), 3.44 (q, *J* = 7.0 Hz, 4H, 4H, NC*H*_2_CH_3_), 2.33 – 2.25 (m, 1H, 2‘-*H*_a_), 2.25 – 2.17 (m, 1H, 2‘-*H*_b_), 1.12 (t, *J* = 7.0 Hz, 6H, NCH_2_C*H*_3_), 0.88 (s, 9H, *t*Bu-C*H*_3_), 0.87 (s, 9H, *t*Bu-C*H*_3_), 0.09 (s, 3H, Si-C*H*_3_), 0.09 (s, 3H, Si-C*H*_3_), 0.08 (s, 6H, 2 x Si-C*H*_3_).

**^13^C-NMR** (126 MHz, DMSO- *d*_6_) δ 160.54 (d, *J*_C-F_ = 13.5 Hz), 160.52, 155.86, 151.94, 150.54, 149.65, 135.49 (d, *J*_C-F_ = 242 Hz), 129.08 (d, *J*_C-F_ = 31.2 Hz), 125.58, 108.82, 105.65, 105.21, 96.89, 87.11, 86.03, 70.53, 64.10, 61.88, 43.99, 40.57, 25.71, 25.62, 17.99, 17.64, 12.29, -4.75, -5.04,-5.61, -5.66.

**^19^F-NMR** (471 MHz, DMSO-*d*_6_) δ -170.14.

**TLC:** R*_f_* = 0.67 (EtOAc/*c*-Hex 1:1).

**HRMS:** *m/z* calculated for C_35_H_55_FN_3_O_7_Si_2_^+^ [M-H^+^] 704.3557, found 704.3784 [M-H^+^].

## 7.4.8 4‑((7‑(Diethylamino)‑2‑oxo‑2*H*‑chromen‑4‑yl)methoxy)‑5‑fluoro‑1‑((2*R*,4*S*,*5R*)‑4-hydroxy-5-(hydroxymethyl)tetrahydrofuran-2-yl)pyrimidin-2(1*H*)-one (DEACM-Floxuridine, 2c)

Following general procedure C with **TBDMS-DEACM-floxuridine** (**2c.2**, 0.120 g, 0.170 mmol) in 12.5 mL THF/AcOH (4:1), TBAF (1 mol/L, in THF, 0.38 mL, 0.38 mmol) was added over 20 minutes. The solution was stirred at ambient temperature and additional TBAF (0.20 mL, 0.20 mmol) was added. After two days (in this case no aqueous workup) the solvents were removed under reduced pressure and the residue was purified by column chromatography (gradient: EtOAc/Acetone 2:1 - EtOAc/Acetone 1:1). The desired compound was obtained as a yellow solid (0.058 g, 0.12 mmol, 72 %).

**^1^H-NMR** (500 MHz, DMSO-*d*_6_) δ 8.59 (d, *J* = 6.5 Hz, 1H, Ar-*H*), 7.50 (d, *J* = 9.0 Hz, 1H, DEACM-*H*5), 6.72 (dd, *J* = 9.1, 2.6 Hz, 1H, DEACM-*H*6), 6.57 (d, *J* = 2.6 Hz, 1H, DEACM-*H*8), 6.08 (dd, *J* = 6.1, 1.7 Hz, 1H, 1‘-*H*), 6.03 (d, *J* = 1.2 Hz, 1H, DEACM-*H*3), 5.68 – 5.54 (m, 2H, DEACM-C*H*_2_), 5.26 (d, *J* = 4.4 Hz, 1H, 3‘-O*H*), 5.21 (t, *J* = 5.0 Hz, 1H, 5‘-O*H*), 4.28 – 4.21 (m, 1H, 3‘-*H*), 3.86 – 3.81 (m, 1H, 4‘-*H*), 3.71 – 3.64 (m, 1H, 5‘-*H*_a_), 3.63 – 3.56 (m, 1H, 5‘-*H*_b_), 3.44 (q, *J* = 7.0 Hz, 4H, NC*H*_2_CH_3_), 2.32 – 2.24 (m, 1H, 2‘-*H*_a_), 2.15 – 2.05 (m, 1H, 2‘-*H*_b_), 1.12 (t, *J* = 7.1 Hz, 6H, NCH_2_C*H*_3_).

**^13^C-NMR** (126 MHz, DMSO-*d*_6_) δ 160.56, 160.48 (d, *J*_C-F_ = 12.8 Hz), 155.87, 152.09, 150.55, 149.74, 135.49 (d, *J*_C-F_ = 242 Hz), 129.74 (d, *J*_C-F_ = 31.6 Hz), 125.60, 108.85, 105.60, 105.22, 96.89, 87.80, 86.21, 69.46, 64.06, 60.53, 44.01, 40.73, 12.31.

**^19^F-NMR** (471 MHz, DMSO-*d*_6_) δ -170.19.

**HRMS:** *m/z* calculated for C_23_H_26_FN_3_NaO_7_^+^ [M-Na^+^] 498.16470, found 498.16407 [M-Na^+^].

**TLC:** R*_f_* = 0.34 (EtOAc/Acetone 2:1).

## 7.4.9 1‑((2*R*,4*S*,5*R*)‑4‑((*tert*‑Butyldimethylsilyl)oxy)‑5‑(((*tert*‑butyldimethylsilyl)oxy)methyl)tetrahydrofuran‑2‑yl)‑4‑((4‑((7‑(diethylamino)‑2‑oxo‑2*H*‑chromen‑4‑yl)methoxy)benzyl)oxy)-5-fluoropyrimidin-2(1*H*)-one (TBDMS-DEACM-Bn-Floxuridine, 6.2)

A flask was charged with **triazoyl-TBDMS-floxuridine 2c.1** (120 mg, 0.228 mmol) and 1,8‑diazabicyclo[5.4.0]undec-7-ene (34 µmol, 0.23 mmol) in 2 mL dry CH_2_Cl_2_. A solution of **DEACM‑Bn‑OH** (73 mg, 0.21 mmol) in 2 mL dry CH_2_Cl_2_ was added. The reaction mixture was stirred for 24 h. The solvent was removed under reduced pressure and the crude product was purified by column chromatography (*c*-Hex/EtOAc 3:1, silica gel deactivated with 1% NEt_3_) to obtain the desired product as a yellow solid (94 mg, 0.94 mmol, 51%).

**^1^H-NMR** (500 MHz, DMSO-*d*_6_) δ 8.22 (d, *J* = 6.3 Hz, 1H, Ar-*H*), 7.58 (d, *J* = 9.0 Hz, 1H, DEACM-*H*5), 7.45 (d, *J* = 8.7 Hz, 2H, Ph-*H*), 7.14 (d, *J* = 8.7 Hz, 2H, Ph-*H*), 6.72 (dd, *J* = 9.1, 2.6 Hz, 1H, DEACM-*H*6), 6.55 (d, *J* = 2.5 Hz, 1H, DEACM-*H*8), 6.13 (s, 1H, DEACM-*H*3), 6.06 (td, *J* = 6.0, 1.7 Hz, 1H, 1’-*H*), 5.39 – 5.31 (m, 4H, DEACM-C*H*_2_ + Ph-C*H*_2_), 4.42 – 4.35 (m, 1H, 3‘-*H*), 3.89 – 3.83 (m, 2H, 4‘-*H* + 5‘-*H*_a_), 3.74 (dd, *J* = 12.5, 4.1 Hz, 1H, 5‘-*H*_b_), 3.44 (q, *J* = 7.0 Hz, 4H, NC*H*_2_CH_3_), 2.30 – 2.17 (m, 2H, 2‘-*H*_a_*H*_b_), 1.12 (t, *J* = 7.0 Hz, 6H, NCH_2_C*H*_3_), 0.87 (s, 9H, *t*Bu-C*H*_3_), 0.87 (s, 9H, *t*Bu-C*H*_3_), 0.08 (s, 3H, Si-C*H*_3_), 0.08 (s, 9H, 3 x Si-C*H*_3_).

**^13^C-NMR** (126 MHz, DMSO-*d*_6_) δ 161.12 (d, *J*_C-F_ = 12.8 Hz), 160.70, 157.91, 155.87, 152.14, 151.39, 150.45, 135.61 (d, *J*_C-F_ = 242 Hz), 130.61, 128.42 (d, *J*_C-F_ = 31.3 Hz), 127.93, 125.70, 114.87, 108.69, 105.40, 105.25, 96.84, 87.03, 85.85, 70.56, 68.43, 65.30, 61.89, 43.97, 40.53, 25.70, 25.62, 17.98, 17.64, 12.30, -4.76, -5.05, -5.63, -5.67.

**^19^F-NMR** (471 MHz, DMSO-*d*_6_) δ -169.87.

**HRMS:** *m/z* calculated for C_42_H_61_FN_3_O_8_Si_2_^+^ [M-H^+^] 810.3976, found 810.4001 [M-H^+^].

**TLC:** R*_f_* = 0.36 (*c*-Hex/EtOAc 3:1).

## 7.4.10 4‑((4‑((7‑(Diethylamino)‑2‑oxo‑2*H*‑chromen‑4‑yl)methoxy)benzyl)oxy)‑5‑fluoro‑1-((2*R*,4*S*,5*R*)-4-hydroxy-5-(hydroxymethyl)tetrahydrofuran-2-yl)pyrimidin-2(1*H*)-one (DEACM-Bn-Floxuridine, 6)

Following the general procedure C, **TBDMS-Bn-DEACM-Floxuridine** (**6.2**, 30 mg, 0.037 mmol) was dissolved in 2 mL THF/AcOH (4:1) and TBAF (1 mol/L solution, 0.23 mL, 0.23 mmol) was added. It was stirred in the dark overnight. The crude product was purified by column chromatography (CH_2_Cl_2_/MeOH 95:5, silica gel deactivated with 1% NEt_3_) and after another wash with saturated NaHCO_3_ solution the desired product was obtained as a green solid (12 mg, 0.021 mmol, 56%).

**^1^H-NMR** (500 MHz, DMSO-*d*_6_) δ 8.50 (d, *J* = 6.6 Hz, 1H, Ar-*H*), 7.58 (d, *J* = 9.0 Hz, 1H, DEACM-*H*5), 7.45 (d, *J* = 8.7 Hz, 2H, Ph-*H*), 7.14 (d, *J* = 8.7 Hz, 2H, Ph-*H*), 6.72 (dd, *J* = 9.1, 2.6 Hz, 1H, DEACM-*H*6), 6.55 (d, *J* = 2.5 Hz, 1H, DEACM-*H*8), 6.13 (s, 1H, DEACM-*H*3), 6.08 (td, *J* = 6.2, 1.8 Hz, 1H, 1’-*H*), 5.39 – 5.32 (m, 4H, DEACM-C*H*_2_ + Ph-C*H*_2_), 5.25 (d, *J* = 4.3 Hz, 1H, 3‘-O*H*), 5.17 (t, *J* = 5.0 Hz, 1H, 5‘-O*H*), 4.27 – 4.20 (m, 1H, 3‘-*H*), 3.83 (q, *J* = 3.5 Hz, 1H, 4‘-*H*), 3.69 – 3.63 (m, 1H, 5‘-*H*_a_), 3.62 – 3.56 (m, 1H, 5‘-*H*_b_), 3.44 (q, *J* = 7.0 Hz, 4H, NC*H*_2_CH_3_), 2.29 – 2.22 (m, 1H, 2‘-*H*_a_), 2.10 – 2.03 (m, 1H, 2‘-*H*_b_), 1.13 (t, *J* = 7.0 Hz, 6H, NCH_2_C*H*_3_).

**^13^C-NMR** (126 MHz, DMSO-*d*_6_) δ 161.06 (d, *J*_C-F_ = 12.8 Hz), 160.72, 157.91, 155.87, 152.26, 151.41, 150.46, 135.59 (d, *J*_C-F_ = 242 Hz), 130.62, 129.07 (d, *J*_C-F_ = 31.9 Hz), 127.97, 125.72, 114.88, 108.70, 105.41, 105.25, 96.84, 87.74, 86.05, 69.53, 68.36, 65.31, 60.57, 43.98, 40.70, 12.30.

**^19^F-NMR** (471 MHz, DMSO-*d*_6_) δ -169.96.

**HRMS:** *m/z* calculated for C_30_H_33_FN_3_O_8_^+^ [M-H^+^] 582.2246, found 582.2256 [M-H^+^].

**TLC:** R*_f_* = 0.22 (CH_2_Cl_2_/MeOH 95:5).

## 7.4.11 (7-(Diethylamino)-2-oxo-2*H*-chromen-4-yl)methyl(((2*R*,3*S*,5*R*)-5-(5-fluoro-2,4-dioxo-3,4-dihydropyrimidin-1(2*H*)-yl)-3-hydroxytetrahydrofuran-2-yl)methyl) carbonate (5’-DEACM-Floxuridine, 5a)

Floxuridine (0.085 g, 0.34 mmol), 4-dimethylaminopyridine (0.001 g, 0.01 mmol) and **DEACM-PFPC** (0.125 g, 0.273 mmol) were dissolved in 12 mL dry DMF and stirred at ambient temperature overnight. The solvent was removed under reduced pressure and the crude product was purified with flash-column chromatography (CH_2_Cl_2_ 100% - CH_2_Cl_2_/MeOH 95:5). The desired product was obtained as a yellow solid (0.030 g, 0.058 mmol, 21%).

**^1^H-NMR** (500 MHz, DMSO-*d*_6_) δ 11.85 (s, 1H, N-*H*), 7.88 (d, *J* = 7.0 Hz, 1H, Ar-*H*), 7.46 (d, *J* = 9.0 Hz, 1H, DEACM-*H*5), 6.69 (dd, *J* = 9.1, 2.6 Hz, 1H, DEACM-*H*6), 6.54 (d, *J* = 2.5 Hz, 1H, DEACM-*H*8), 6.16 (td, *J* = 6.8, 1.8 Hz, 1H, 1‘-*H*), 5.97 (s, 1H, DEACM-*H*3), 5.47 (d, *J* = 4.4 Hz, 1H, 3‘-O*H*), 5.36 (s, 2H, DEACM-C*H*_2_), 4.47 – 4.29 (m, 2H, 5‘-*H*_a_*H*_b_), 4.30 – 4.18 (m, 1H, 3‘-*H*), 3.98 – 3.93 (m, 1H, 4‘-*H*), 3.43 (q, *J* = 7.0 Hz, 4H, NC*H*_2_CH_3_), 2.26 – 2.08 (m, 2H, 2‘-*H*_a_*H*_b_), 1.12 (t, *J* = 7.0 Hz, 6H, NCH_2_C*H*_3_).

**^13^C-NMR** (126 MHz, DMSO-*d*_6_) δ 160.51, 157.15 (d, *J*_C-F_ = 26.0 Hz), 155.86, 153.97, 150.53, 149.75, 149.14, 140.16 (d, *J*_C-F_ = 232 Hz), 125.55, 124.59 (d, *J*_C-F_ = 34.3 Hz), 108.77, 105.34, 105.10, 96.87, 84.53, 83.49, 69.87, 67.86, 64.83, 44.00, 38.46, 12.30.

**^19^F-NMR** (471 MHz, DMSO-*d*_6_) δ -166.71.

**HRMS:** *m/z* calculated for C_24_H_27_FN_3_O_9_^+^ [M-H^+^] 520.1726, found 520.1735 [M-H^+^].

**TLC:** R*_f_* = 0.15 (CH_2_Cl_2_/MeOH 19:1).

## 7.4.12 4‑Amino‑1‑((2*R*,4*R*,5*R*)‑4‑((*tert*‑butyldimethylsilyl)oxy)‑5‑(((*tert*‑butyldimethylsilyl)oxy)methyl)-3,3-difluorotetrahydrofuran-2-yl)pyrimidin-2(1*H*)-one (TBDMS-Gemcitabine, 3a.1)

Following the general procedure A, **TBDMS-Gemcitabine** (**3a.1**) was obtained from the protection of gemcitabine (500 mg, 1.89 mmol) with imidazole (515 mg, 7.57 mmol) and TBDMSCl (1.71 g, 11.4 mmol) in dry DMF (10 mL) for 16 h, as a colorless solid (650 mg, 1.32 mmol, 70%) after column chromatography (EtOAc/MeOH 95:5).

**^1^H-NMR** (500 MHz, DMSO-*d*_6_) δ 7.53 (d, *J* = 7.5 Hz, 1H, Ar-*H*), 7.40 (s, 2H, N-*H*_2_), 6.17 (t, *J* = 8.0 Hz, 1H, 1‘‑*H*), 5.77 (d, *J* = 7.5 Hz, 1H, Ar-*H*), 4.35 – 4.37 (m, 1H, 3‘-*H*), 3.94 (dd, *J* = 12.2, 2.3 Hz, 1H, 5‘-*H*_a_), 3.89 (dt, *J* = 8.4, 2.7 Hz, 1H, 4‘-*H*), 3.78 (dd, *J* = 12.1, 3.1 Hz, 1H, 5‘-*H*_b_), 0.90 (s, 9H, *t*Bu-C*H*_3_), 0.89 (s, 9H, *t*Bu-C*H*_3_), 0.11 (s, 3H, Si-C*H*_3_), 0.10 (s, 3H, Si-C*H*_3_), 0.09 (s, 3H, Si-C*H*_3_), 0.09 (s, 3H, Si-C*H*_3_).

**^13^C-NMR** (126 MHz, DMSO-*d*_6_) δ 165.57, 154.46, 139.79, 122.45 (t, *J_C-F_* = 259 Hz), 94.68, 83.52 (m), 79.68, 69.57 (t, *J_C-F_* = 22.3 Hz), 60.26, 25.66, 25.33, 17.91, 17.66, -5.00, -5.33, -5.60.

**^19^F-NMR** (471 MHz, DMSO-*d*_6_) δ -115.73.

**HRMS:** *m/z* calculated for C_21_H_40_F_2_N_3_O_4_Si_2_^+^ [M-H^+^] 492.2520, found 492.2540 [M-H^+^].

**TLC:** R*_f_* = 0.31 (EtOAc/MeOH 95:5).

## 7.4.13 4‑Amino‑1‑((2*R*,4*S*,5*R*)‑4‑((*tert*‑butyldimethylsilyl)oxy)‑5‑(((*tert*‑butyldimethylsilyl)oxy)methyl)tetrahydrofuran-2-yl)-1,3,5-triazin-2(1*H*)-one (TBDMS-Decitabine, 3c.1)

Following the general procedure A, **TBDMS-decitabine** (**3c.1**) was obtained from the protection of decitabine (100 mg, 0.440 mmol) and imidazole (239 mg, 3.51 mmol) and TBDMSCl (528 mg, 3.51 mmol) in dry DMF (10 mL) for 4 d, as a colorless solid (180 mg, 0.39 mmol, 90%).

**^1^H-NMR** (500 MHz, DMSO-*d*_6_) δ 8.35 (s, 1H, Ar-*H*), 7.51 (s, 2H, N-*H*_2_), 6.02 (t, *J* = 6.3 Hz, 1H, 1‘-*H*), 4.39 (dt, *J* = 5.9, 4.3 Hz, 1H, 3‘-*H*), 3.86 – 3.78 (m, 2H, 4‘-*H* + 5‘-*H*_a_), 3.71 (dd, *J* = 11.0, 3.1 Hz, 1H, 5‘-*H*_b_), 2.30 – 2.18 (m, 2H, 2‘-*H*_a_*H*_b_), 0.88 (s, 9H, *t*Bu-C*H*_3_), 0.87 (s, 9H, *t*Bu-C*H*_3_), 0.08 (s, 6H, 2 x Si-C*H*_3_), 0.07 (s, 6H, 2 x Si-C*H*_3_).

**^13^C-NMR** (126 MHz, DMSO-*d*_6_) δ 165.88, 155.47, 152.98, 86.93, 84.95, 71.10, 62.09, 40.43, 25.78, 25.63, 17.97, 17.66, -4.79, -5.02, -5.53, -5.61.

**HRMS:** *m/z* calculated for C_20_H_41_N_4_O_4_Si_2_^+^ [M-H^+^] 457.2661, found 457.2657 [M-H^+^].

**TLC:** R*_f_* = 0.93 (EtOAc).

## 7.4.14 (7‑(Diethylamino)‑2‑oxo‑2*H*‑chromen‑4‑yl)methyl (1‑((2*R*,4*R*,5*R*)‑4‑((*tert*‑butyldimethylsilyl)oxy)‑5‑(((*tert*‑butyldimethylsilyl)oxy)methyl)‑3,3‑difluorotetrahydrofuran-2-yl)-2-oxo-1,2-dihydropyrimidin-4-yl)carbamate (TBDMS-DEACM-Gemcitabine, 3a.2)

Following general procedure B, **TBDMS-DEACM-gemcitabine** (**3a.2**) was obtained from **TBDMS-gemcitabine** (**3a.1**) (160 mg, 0.325 mmol) and **DEACM-CDI** (111 mg, 0.325 mmol) with LiHMDS (0.04 mL, 0.4 mmol) in dry THF (11 mL), as a pale yellow solid (100 mg, 0.130 mmol, 40%) after column chromatography (*c*-Hex/EtOAc 1:2, silica gel deactivated with 1% NEt_3_).

**^1^H-NMR** (500 MHz, DMSO-*d*_6_) δ 11.26 (s, 1H, N-*H*), 8.06 (d, *J* = 7.6 Hz, 1H, Ar-*H*), 7.46 (d, *J* = 9.1 Hz, 1H, DEACM-*H*5), 7.12 (d, *J* = 7.7 Hz, 1H, Ar-*H*), 6.71 (dd, *J* = 9.1, 2.6 Hz, 1H, DEACM-*H*6), 6.56 (d, *J* = 2.5 Hz, 1H, DEACM-*H*8), 6.22 (t, *J* = 7.3 Hz, 1H, 1‘-*H*), 6.16 (s, 1H, DEACM-*H*3), 5.40 (s, 2H, DEACM-C*H*_2_), 4.43 – 4.32 (m, 1H, 3‘-*H*), 4.07 – 3.93 (m, 2H, 4‘-*H* + 5‘-*H*_a_), 3.82 (dd, *J* = 12.5, 3.3 Hz, 1H, 5‘-*H*_b_), 3.44 (q, *J* = 7.1 Hz, 4H, NC*H*_2_CH_3_), 1.12 (t, *J* = 7.0 Hz, 6H, NCH_2_C*H*_3_), 0.91 (s, 9H, *t*Bu-C*H*_3_), 0.89 (s, 9H, *t*Bu-C*H*_3_), 0.11 (s, 3H, Si-C*H*_3_), 0.10 (m, 9H, 3 x Si-C*H*_3_).

**^13^C-NMR** (126 MHz, DMSO-*d*_6_) δ 163.40, 160.77, 155.81, 153.89, 152.56, 150.56, 150.42, 143.62, 125.43, 122.36 (t, *J_C-F_* = 261 Hz), 108.84, 105.09, 104.81, 96.91, 94.97, 84.40 (m), 80.44, 69.37 (m), 62.42, 60.33, 44.05, 25.72, 25.37, 17.97, 17.71, 12.34, -4.94, -5.28, -5.54, -5.57.

**^19^F-NMR** (471 MHz, DMSO-*d*_6_) δ -115.87.

**HRMS:** *m/z* calculated for C_36_H_55_F_2_N_4_O_8_Si_2_^+^ [M-H^+^] 765.3521, found 765.3540 [M-H^+^].

**TLC:** R*_f_* = 0.63 (*c*-Hex /EtOAc 1:2).

## 7.4.15 4-((7-(Diethylamino)-2-oxo-2*H*-chromen-4-yl)methoxy)benzyl 1*H*-imidazole-1-carboxylate (DEACM-Bn-CDI)

A microwave vessel was charged with **DEACM-Bn-OH** (30 mg, 0.085 mmol) and 1,1‘‑carbonyldiimidazole (21 mg, 0.13 mmol) and combined with 2 mL dry CH_2_Cl_2_. The reaction was heated at 50 °C for 2 h in a microwave reactor. Afterwards, it was transferred into a separation funnel, diluted with CH_2_Cl_2_ and extracted with distilled H_2_O three times. The organic layer was dried over Na_2_SO_4_ and the solvent was removed under reduced pressure to obtain the desired product as a yellow-brownish solid (38 mg, 0.085 mmol, quant.).

**^1^H-NMR** (600 MHz, DMSO-*d*_6_) δ 8.26 (t, *J* = 1.0 Hz, 1H, Imidazole-*H*), 7.60 (t, *J* = 1.5 Hz, 1H, Imidazole-*H*), 7.58 (d, *J* = 9.1 Hz, 1H, DEACM-*H*5), 7.49 (d, *J* = 8.7 Hz, 2H, Ph -*H*), 7.15 (d, *J* = 8.7 Hz, 2H, Ph -*H*), 7.07 (dd, *J* = 1.7, 0.9 Hz, 1H, Imidazole-*H*), 6.72 (dd, *J* = 9.1, 2.6 Hz, 1H, DEACM-*H*6), 6.55 (d, *J* = 2.6 Hz, 1H, DEACM-*H*8), 6.12 (t, *J* = 1.3 Hz, 1H, DEACM-*H*3), 5.39 (s, 2H, Ph-C*H*_2_), 5.35 (d, *J* = 1.3 Hz, 2H, DEACM-C*H*_2_), 3.44 (q, *J* = 7.0 Hz, 4H, NC*H*_2_CH_3_), 1.12 (t, *J* = 7.1 Hz, 6H, NCH_2_C*H*_3_).

**^13^C-NMR** (151 MHz, DMSO-*d*_6_) δ 160.69, 158.03, 155.85, 151.36, 150.45, 148.30, 137.20, 130.49, 130.33, 127.39, 125.69, 117.50, 114.93, 108.68, 105.39, 105.24, 96.84, 69.09, 65.29, 43.95, 12.28.

**HRMS:** *m/z* calculated for C_25_H_26_N_3_O_5_^+^ [M-H^+^] 448.1867, found 448.1866 [M-H^+^].

**TLC:** R*_f_* = 0.29 (*c*-Hex/EtOAc 1:2).

## 7.4.16 4‑((7‑(Diethylamino)‑2‑oxo‑2H‑chromen‑4‑yl)methoxy)benzyl (1‑((2*R*,4*R*,*5R*)-4-((*tert*-butyldimethylsilyl)oxy)-5-(((*tert*-butyldimethylsilyl)oxy)methyl)-3,3-difluorotetrahydrofuran-2-yl)-2-oxo-1,2-dihydropyrimidin-4-yl)carbamate (TBDMS-DEACM-Bn-Gemcitabine, 7.1)

Following the general procedure B, **TBDMS-DEACM-Bn-gemcitabine** (**7.1**) was obtained from **TBDMS-gemcitabine** (**3a.1**, 44 mg, 0.089 mmol) and **DEACM-Bn-CDI** (40 mg, 0.089 mmol) with LiHMDS (0.11 mL, 0.11 mmol) in dry THF (4 mL), as a reddish brown solid (57 mg, 0.065 mmol, 73%) after column chromatography (*c*‑Hex/EtOAc 1:2, silica gel deactivated with 1% NEt_3_).

**^1^H-NMR** (500 MHz, DMSO-*d*_6_) δ 10.96 (s, 1H, N-*H*), 8.01 (d, *J* = 7.6 Hz, 1H, Ar-*H*), 7.58 (d, *J* = 9.0 Hz, 1H, DEACM-*H*5), 7.39 (d, *J* = 8.7 Hz, 2H, Ph-*H*), 7.15 – 7.10 (m, 3H, Ar-*H*, Ph-*H*), 6.71 (dd, *J* = 9.1, 2.6 Hz, 1H, DEACM-*H*6), 6.55 (d, *J* = 2.5 Hz, 1H, DEACM-*H*8), 6.21 (t, *J* = 7.3 Hz, 1H, 1’-*H*), 6.11 (d, *J* = 1.4 Hz, 1H, DEACM-*H*3), 5.33 (d, *J* = 1.3 Hz, 2H, DEACM-C*H*_2_), 5.14 (s, 2H, Ph-C*H*_2_), 4.43 – 4.30 (m, 1H, 3‘-*H*), 4.01 – 3.93 (m, 2H, 4‘-*H* + 5‘-*H*_a_), 3.85 – 3.77 (m, 1H, 5‘-*H*_b_), 3.44 (q, *J* = 7.0 Hz, 4H, NC*H*_2_CH_3_), 1.12 (t, *J* = 7.0 Hz, 6H, NCH_2_C*H*_3_), 0.90 (s, 9H, *t*Bu-C*H*_3_), 0.88 (s, 9H, *t*Bu-C*H*_3_), 0.11 (s, 3H, Si-C*H*_3_), 0.10 (s, 3H, Si-C*H*_3_), 0.10 (s, 6H, 2 x Si-C*H*_3_).

**^13^C-NMR** (126 MHz, DMSO-*d*_6_) δ 163.42, 160.71, 157.72, 155.86, 153.82, 153.04, 151.42, 150.45, 143.33, 130.07, 128.45, 125.69, 122.28 (t, *J_C-F_* = 259 Hz), 114.85, 108.68, 105.39, 105.21, 96.84, 94.93, 84.10 (m), 80.33, 69.38 (t, *J_C-F_* = 22.1 Hz), 66.58, 65.27, 60.28, 43.97, 25.66, 25.31, 17.92, 17.64, 12.29, -5.01, -5.35, -5.60, -5.62.

**^19^F-NMR** (471 MHz, DMSO-*d*_6_) δ -115.89.

**HRMS:** *m/z* calculated for C_43_H_61_F_2_N_4_O_9_Si_2_^+^ [M-H^+^] 871.3940, found 871.3963 [M-H^+^].

**TLC:** R*_f_* = 0.56 (*c*-Hex/EtOAc 1:2).

## 7.4.17 (7-(Diethylamino)-2-oxo-2*H*-chromen-4-yl)methyl (5-((2*R*,4*S*,*5R*)-4-((*tert*-butyldimethylsilyl)oxy)-5-(((*tert*-butyldimethylsilyl)oxy)methyl)tetrahydrofuran-2-yl)-4-oxo-4,5-dihydro-1,3,5-triazin-2-yl)carbamate (TBDMS-DEACM-Decitabine, 3c.2)

Following the general procedure B, **TBDMS-DEACM-decitabine** (**3c.2**) was obtained from **TBDMS-decitabine** (**3c.1**, 100 mg, 0.219 mmol) and **DEACM-CDI** (75 mg, 0.22 mmol) with LiHMDS (0.26 mL, 0.26 mmol) in dry THF (5 mL), as a reddish brown solid (63 mg, 0.086 mmol, 40%) after column chromatography (*c*-Hex/EtOAc 1:2, silica gel deactivated with 1% NEt_3_).

**^1^H-NMR** (400 MHz, DMSO-*d*_6_) δ 11.03 (s, 1H, N-*H*), 8.66 (s, 1H, Ar-*H*), 7.47 (d, *J* = 9.1 Hz, 1H, DEACM-*H*5), 6.69 (dd, *J* = 9.1, 2.6 Hz, 1H, DEACM-*H*6), 6.55 (d, *J* = 2.5 Hz, 1H, DEACM-*H*8), 6.20 (s, 1H, DEACM-*H*3), 5.99 (t, *J* = 5.8 Hz, 1H, 1’-*H*), 5.37 (s, 2H, DEACM-C*H*_2_), 4.44 – 4.36 (m, 1H, 3‘-*H*), 3.90 (q, *J* = 3.8 Hz, 1H, 4‘-*H*), 3.85 (dd, *J* = 11.4, 3.9 Hz, 1H, 5‘-*H*_a_), 3.77 – 3.71 (m, 1H, 5‘-*H*_b_), 3.43 (q, *J* = 7.0 Hz, 4H, NC*H*_2_CH_3_), 2.42 – 2.27 (m, 2H, 2‘-*H*_a_*H*_b_), 1.12 (t, *J* = 7.0 Hz, 6H, NCH_2_C*H*_3_), 0.87 (s, 9H, *t*Bu-C*H*_3_), 0.87 (s, 9H, *t*Bu-C*H*_3_), 0.08 (s, 6H, 2 x Si-C*H*_3_), 0.07 (s, 6H, 2 x Si-C*H*_3_).

**^13^C-NMR** (101 MHz, DMSO-*d*_6_) δ 163.40, 160.72, 157.14, 155.73, 152.91, 150.65, 150.45, 150.14, 125.37, 108.72, 105.05, 104.63, 96.85, 87.34, 86.32, 70.49, 61.93, 61.75, 43.99, 40.30, 25.78, 25.63, 17.98, 17.64, 12.30, -4.77, -5.05, -5.54, -5.60.

**HRMS:** *m/z* calculated for C_35_H_56_N_5_O_8_Si_2_^+^ [M-H^+^] 730.3662, found 730.3659 [M-H^+^].

**TLC:** R*_f_* = 0.61 (*c*-Hex/EtOAc 1:2).

## 7.4.18 (7-(Diethylamino)-2-oxo-2*H*-chromen-4-yl)methyl (1-((2*R*,4*R*,5*R*)-3,3-difluoro-4-hydroxy-5-(hydroxymethyl)tetrahydrofuran-2-yl)-2-oxo-1,2-dihydropyrimidin-4-yl)carbamate (DEACM-Gemcitabine, 3a)

Method 1:

Following the general procedure C, **DEACM-gemcitabine** (**3a**) was obtained from the deprotection of **TBDMS-DEACM-gemcitabine** (**3a.2**, 90 mg, 0.12 mmol) with TBAF (1 mol/L solution, 0.70 mL, 0.70 mmol) in THF/AcOH (5 mL, 4:1) for 16 h, as bright green solid (30 mg, 0.035 mmol, 48%) after column chromatography (CH_2_Cl_2_/MeOH 0-10%, silica gel deactivated with 1% NEt_3_).

Method 2:

Gemcitabine (100 mg, 0.380 mmol) was dissolved in 3 mL dry pyridine and stirred for 15 min at ambient temperature. **DEACM-PFPC** (87 mg, 0.18 mmol) was added and the mixture stirred in the dark for 18 h. The reaction mixture was transferred into a separation funnel and washed with water. The organic phases were collected, dried over Na_2_SO_4_, filtered, the solvent was removed under reduced pressure and the crude product was purified by column chromatography (CH_2_Cl_2_/MeOH 5-10%, silica gel deactivated with 0.5% NEt_3_) to obtain the desired product as a green solid (19 mg, 0.035 mmol, 9%).

**^1^H-NMR** (500 MHz, DMSO-*d*_6_) δ 11.21 (s, 1H, N-*H*), 8.26 (d, *J* = 7.7 Hz, 1H, Ar-*H*), 7.47 (d, *J* = 9.1 Hz, 1H, DEACM-*H*5), 7.10 (d, *J* = 7.6 Hz, 1H, Ar-*H*), 6.70 (dd, *J* = 9.1, 2.6 Hz, 1H, DEACM-*H*6), 6.56 (d, *J* = 2.5 Hz, 1H, DEACM-*H*8), 6.31 (d, *J* = 6.5 Hz, 1H, 3‘-O*H*), 6.22 – 6.14 (m, 2H, 1‘-*H* + DEACM-*H3*), 5.40 (s, 2H, DEACM-C*H*_2_), 5.29 (t, *J* = 5.5 Hz, 1H, 5‘-O*H*), 4.29 – 4.11 (m, 1H, 3‘-*H*), 3.91 – 3.87 (m, 1H, 4‘-*H*), 3.85 – 3.76 (m, 1H, 5‘-*H*_a_), 3.73 – 3.60 (m, 1H, 5‘-*H*_b_), 3.44 (q, *J* = 7.1 Hz, 4H, NC*H*_2_CH_3_), 1.12 (t, *J* = 7.0 Hz, 6H, NCH_2_C*H*_3_).

**^13^C-NMR** (126 MHz, DMSO) δ 163.31, 160.70, 155.76, 153.98, 152.46, 150.49, 150.40, 144.65, 125.39, 122.95 (t, *J_C-F_* = 259 Hz), 108.77, 105.05, 104.78, 96.87, 94.83, 84.13 (m), 81.02 (m), 68.40 (t, *J_C-F_* = 22.3 Hz), 62.30, 58.80, 44.00, 12.30.

**^19^F-NMR** (471 MHz, DMSO-*d*_6_) δ -116.85.

**HRMS:** *m/z* calculated for C_24_H_27_F_2_N_4_O_8_^+^ [M-H^+^] 537.1792, found 537.1783 [M-H^+^].

**TLC:** R*_f_* = 0.21 (CH_2_Cl_2_/MeOH 95:5).

## 7.4.19 4-((7-(Diethylamino)-2-oxo-2*H*-chromen-4-yl)methoxy)benzyl (1-((2*R*,4*R*, 5*R*)-3,3-difluoro-4-hydroxy-5-(hydroxymethyl)tetrahydrofuran-2-yl)-2-oxo-1,2-dihydropyrimidin-4-yl)carbamate (DEACM-Bn-Gemcitabine, 7)

Following the general procedure C, the desired **DEACM-Bn-gemcitabine** (**7**) was obtained by the deprotection of the TBDMS-protected compound **7.1** (57 mg, 0.065 mmol) with TBAF (1 mol/L solution, 0.40 mL, 0.40 mmol) in THF/AcOH (3 mL, 4:1) for 16 h, as a bright green solid (21 mg, 0.065 mmol, 50%) after column chromatography (CH_2_Cl_2_/MeOH 0-10%, silica gel deactivated with 1% NEt_3_).

**^1^H-NMR** (500 MHz, DMSO-*d*_6_) δ 10.91 (s, 1H, N-*H*), 8.22 (d, *J* = 7.7 Hz, 1H, Ar-*H*), 7.58 (d, *J* = 9.0 Hz, 1H, DEACM-*H*5), 7.40 (d, *J* = 8.7 Hz, 2H, Ph-*H*), 7.17 – 7.09 (m, 3H, Ar-*H* + Ph-*H*), 6.72 (dd, *J* = 9.1, 2.6 Hz, 1H, DEACM-*H*6), 6.55 (d, *J* = 2.6 Hz, 1H, DEACM-*H*8), 6.30 (d, *J* = 6.5 Hz, 1H, 3‘-O*H*), 6.16 (t, *J* = 7.5 Hz, 1H, 1’-*H*), 6.11 (d, *J* = 1.3 Hz, 1H, DEACM-*H*3), 5.34 (d, *J* = 1.4 Hz, 2H, DEACM-C*H*_2_), 5.29 (t, *J* = 5.5 Hz, 1H, 5‘-O*H*), 5.14 (s, 2H, Ph-C*H*_2_), 4.25 – 4.13 (m, 1H, 3‘-*H*), 3.91 – 3.85 (m, 1H, 4‘-*H*), 3.85 – 3.77 (m, 1H, 5‘-*H*_a_), 3.69 – 3.61 (m, 1H, 5‘-*H*_b_), 3.44 (q, *J* = 7.0 Hz, 4H, NC*H*_2_CH_3_), 1.12 (t, *J* = 7.0 Hz, 6H, NCH_2_C*H*_3_).

^1^**^3^C-NMR** (126 MHz, DMSO-*d*_6_) δ 163.36, 160.72, 157.71, 155.87, 153.97, 153.06, 151.44, 150.46, 144.39, 130.10, 128.50, 125.71, 122.94 (t, *J_C-F_* = 259 Hz), 114.85, 108.70, 105.40, 105.21, 96.84, 94.88, 84.08 (m), 80.98 (m), 68.38 (t, *J_C-F_* = 22.5 Hz), 66.53, 65.27, 58.79, 43.98, 12.30.

**^19^F-NMR** (377 MHz, DMSO-*d*_6_) δ -116.90.

**HRMS:** *m/z* calculated for C_31_H_33_F_2_N_4_O_9_^+^ [M-H^+^] 643.2210, found 643.2214 [M-H^+^].

**TLC:** R*_f_* = 0.17 (CH_2_Cl_2_/MeOH 95:5).

## 7.4.20 (7-(Diethylamino)-2-oxo-2*H*-chromen-4-yl)methyl (5-((2*R*,4*S*,5*R*)-4‑hydroxy-5-(hydroxymethyl)tetrahydrofuran-2-yl)-4-oxo-4,5-dihydro-1,3,5-triazin-2-yl)carbamate (DEACM-decitabine, 3c)

Following the general procedure C, **DEACM-decitabine** (**3c**) was obtained in the deprotection of **TBDMS-DEACM-decitabine** (**3c.2**, 44 mg, 0.060 mmol) with TBAF (1M solution, 0.37 mL, 0.37 mmol) in THF/AcOH (2.5 mL, 4:1) for 20 h, as a bright green solid (12 mg, 0.024 mmol, 40%) after column chromatography (CH_2_Cl_2_/MeOH 0-10%, silica gel deactivated with 1% NEt_3_). The product´s analytics correspond with known literature.^[3]^

## 7.4.21 (7-(Diethylamino)-2-oxo-2*H*-chromen-4-yl)methyl (1-((2*R*,3*S*,4*S*,5*R*)-3,4-dihydroxy-5-(hydroxymethyl)tetrahydrofuran-2-yl)-2-oxo-1,2-dihydropyrimidin-4-yl)carbamate (DEACM-Cytarabine, 3b)

Cytarabine (100 mg, 0.411 mmol) was dissolved in 3 mL dry pyridine and stirred for 15 min at ambient temperature. **DEACM-PFPC** (94 mg, 0.21 mmol) was added and the mixture stirred in the dark for 18 h. The solvent was removed under reduced pressure and the crude product was purified by column chromatography (CH_2_Cl_2_/MeOH 5-10%, silica gel deactivated with 1% NEt_3_) to obtain the desired product **3b** as a brownish-yellow solid (72 mg, 0.14 mmol, 34%).

**^1^H-NMR** (600 MHz, DMSO-*d*_6_) δ 11.01 (s, 1H, N-*H*), 8.08 (d, *J* = 7.5 Hz, 1H, Ar-*H*), 7.46 (d, *J* = 9.0 Hz, 1H, DEACM-*H*5), 7.01 (d, *J* = 7.5 Hz, 1H, Ar-*H*), 6.70 (dd, *J* = 9.1, 2.6 Hz, 1H, DEACM-*H*6), 6.55 (d, *J* = 2.5 Hz, 1H, DEACM-*H*8), 6.15 (s, 1H, DEACM-*H3*), 6.06 (d, *J* = 3.9 Hz, 1H, 1‘-*H*), 5.48 (d, *J* = 5.4 Hz, 1H, 2‘-O*H*), 5.46 (d, *J* = 4.1 Hz, 1H, 3‘-O*H*), 5.39 (t, *J* = 1.3 Hz, 2H, DEACM-C*H*_2_), 5.04 (t, 1H, *J* = 5.6 Hz, 5‘-O*H*), 4.07 (dd, *J* = 4.0, 2.3 Hz, 1H, 2‘-*H*), 3.93 (t, *J* = 2.6 Hz, 1H, 3‘-*H*), 3.84 (td, *J* = 5.4, 2.8 Hz, 1H, 4‘-*H*), 3.62 (d, *J* = 5.5 Hz, 2H, 5‘-*H*_a_*H*_b_), 3.43 (q, *J* = 7.0 Hz, 4H, NC*H*_2_CH_3_), 1.12 (t, *J* = 7.0 Hz, 6H, NCH_2_C*H*_3_).

**^13^C-NMR** (151 MHz, DMSO-*d*_6_) δ 162.47, 160.68, 155.74, 154.17, 152.61, 150.51, 150.47, 146.61, 125.32, 108.75, 105.06, 104.70, 96.86, 93.11, 86.96, 85.78, 76.14, 74.59, 62.07, 61.02, 43.97, 12.27.

**HRMS:** *m/z* calculated for C_24_H_29_N_4_O_9_^+^ [M-H^+^] 517.1929, found 517.1930 [M-H^+^].

**TLC:** R*_f_* = 0.12 (CH_2_Cl_2_/MeOH 85:15).

## 7.4.22 9‑((2*R*,3*S*,4*R*,5*R*)‑4‑((*tert*‑Butyldimethylsilyl)oxy)‑5‑(((*tert‑*butyldimethylsilyl)oxy)methyl)-3-fluorotetrahydrofuran-2-yl)-2-chloro-9*H*-purin-6-amine (TBDMS-Clofarabine, 4a.1)

Following the general procedure A, **TBDMS-clofarabine** (**4a.1**) was obtained from the protection of clofarabine (100 mg, 0.330 mmol) with imidazole (179 mg, 2.63 mmol) and TBDMSCl (298 mg, 1.98 mmol) in dry DMF (3 mL) for 24 h, as a colourless oil (148 mg, 0.280 mmol, 85%) after column chromatography (EtOAc/ *c*-Hex 1:1).

**^1^H-NMR** (500 MHz, DMSO-*d*_6_) δ 8.18 (d, *J* = 1.7 Hz, 1H, Ar-*H*), 7.89 (s, 2H, N-*H*_2_), 6.35 (dd, *J* = 10.2, 5.4 Hz, 1H, 1‘-*H*), 5.38 (dt, *J* = 53.0, 5.2 Hz, 1H, 2‘-*H*), 4.71 (dt, *J* = 19.1, 5.5 Hz, 1H, 3‘-*H*), 3.90 – 3.81 (m, 3H, 5‘-*H*_a_*H*_b_ + 4‘-*H*), 0.89 (s, 9H, *t*Bu-C*H*_3_), 0.89 (s, 9H, *t*Bu-C*H*_3_), 0.13 (s, 3H, Si-C*H*_3_), 0.12 (s, 3H, Si-C*H*_3_), 0.06 (s, 3H, Si-C*H*_3_), 0.05 (s, 3H, Si-C*H*_3_).

**^13^C-NMR** (126 MHz, DMSO-*d*_6_) δ 156.82, 153.31, 150.17, 139.67, 139.65, 117.46, 95.25 (d, *J_C-F_* = 193.7 Hz), 81.83 (d, *J_C-F_* = 7.6 Hz), 80.73 (d, *J_C-F_* = 17.2 Hz), 73.36 (d, *J_C-F_* = 23.1 Hz), 61.51, 25.75, 25.50, 18.02, 17.64, -4.84, -5.15, -5.50, -5.53.

**^19^F-NMR** (471 MHz, DMSO-*d*_6_) δ -198.41.

**HRMS:** *m/z* calculated for C_22_H_40_ClFN_5_O_3_Si_2_^+^ [M-H^+^] 532.2337, found 532.2337 [M-H^+^].

**TLC:** R*_f_* = 0.95 (EtOAc).

## 7.4.23 9‑((2*R*,4*S*,5*R*)‑4‑((t*ert*‑Butyldimethylsilyl)oxy)‑5‑(((*tert*‑butyldimethylsilyl)oxy)methyl)tetrahydrofuran-2-yl)-2-chloro-9*H*-purin-6-amine (TBDMS-Cladribine, 4b.1)

Following the general procedure A, **TBDMS-Cladribine** (**4b.1**) was obtained from the protection of cladribine (100 mg, 0.350 mmol) with imidazole (191 mg, 2.80 mmol) and TBDMSCl (317 mg, 2.10 mmol) in dry DMF (3 mL) for 24 h, as a colourless solid (160 mg, 0.312 mmol, 89%).

**^1^H-NMR** (600 MHz, DMSO-*d*_6_) δ 8.29 (s, 1H, Ar-*H*), 7.79 (s, 2H, N-*H*_2_), 6.24 (t, *J* = 6.5 Hz, 1H, 1‘‑*H*), 4.70 – 4.62 (m, 1H, 3‘-*H*), 3.85 – 3.82 (m, 1H, 4‘-*H*), 3.82 – 3.78 (m, 1H, 5‘-*H*_a_), 3.65 (dd, *J* = 10.8, 4.1 Hz, 1H, 5‘-*H*_b_), 2.89 (dt, *J* = 13.2, 6.2 Hz, 1H, 2‘-*H*_a_), 2.35 – 2.28 (m, 1H, 2‘-*H*_b_), 0.90 (s, 9H, *t*Bu-C*H*_3_), 0.83 (s, 9H, *t*Bu-C*H*_3_), 0.12 (s, 6H, 2 x Si-C*H*_3_), 0.01 (s, 3H, Si-C*H*_3_), -0.01 (s, 3H, Si-C*H*_3_).

**^13^C-NMR** (151 MHz, DMSO-*d*_6_) δ 156.79, 152.92, 149.93, 140.24, 118.34, 86.95, 83.55, 71.75, 62.33, 38.22, 25.70, 25.69, 17.91, 17.70, -4.78, -4.99, -5.59.

**HRMS:** *m/z* calculated for C_22_H_41_ClN_5_O_3_Si_2_^+^ [M-H^+^] 514.2431, found 514.2440 [M-H^+^].

**TLC:** R*_f_* = 0.89 (EtOAc).

## 7.4.24 (7-(Diethylamino)-2-oxo-2*H*-chromen-4-yl)methyl (9-((2*R*,3*S*,4*R*,5*R*)-4-((*tert*‑butyldimethylsilyl)oxy)‑5‑(((*tert*‑butyldimethylsilyl)oxy)methyl)‑3‑fluorotetrahydrofuran-2-yl)-2-chloro-9*H*-purin-6-yl) carbamate (TBDMS-DEACM-Clofarabine, 4a.2)

Following the general procedure B, **TBDMS-DEACM-clofarabine** (**4a.2**) was obtained from **TBDMS-clofarabine** (**4a.1**, 148 mg, 0.278 mmol) and **DEACM-CDI** (95 mg, 0.28 mmol) with LiHMDS (1 mol/L solution, 0.34 mL, 0.34 mmol) in dry THF (5 mL), as a brownish oil (208 mg, 0.260 mmol, 93%) after column chromatography (*c*-Hex/EtOAc 1:1, silica gel deactivated with 1% NEt_3_).

**^1^H-NMR** (500 MHz, DMSO-*d*_6_) δ 11.43 (s, 1H, N-*H*), 8.51 (d, *J* = 1.7 Hz, 1H, Ar-*H*), 7.48 (d, *J* = 9.0 Hz, 1H, DEACM-*H*5), 6.71 (dd, *J* = 9.1, 2.6 Hz, 1H, DEACM-*H*6), 6.56 (d, *J* = 2.6 Hz, 1H, DEACM-*H*8), 6.49 (dd, *J* = 10.5, 5.4 Hz, 1H, 1‘-*H*), 6.36 (s, 1H, DEACM-*H3*), 5.43 (dt + s, *J* = 53.0 Hz, 5.1 Hz, 3H, DEACM-C*H*_2_ + 2‘-*H*), 4.73 (dt, *J* = 18.9, 5.1 Hz, 1H, 3‘-*H*), 3.96 – 3.81 (m, 3H, 5‘-*H*_a_*H*_b_ + 4‘-*H*), 3.44 (q, *J* = 7.0 Hz, 4H, NC*H*_2_CH_3_), 1.12 (t, *J* = 7.0 Hz, 6H, NCH_2_C*H*_3_), 0.90 (s, 9H, *t*Bu-C*H*_3_), 0.88 (s, 9H, *t*Bu-C*H*_3_), 0.14 (s, 3H, Si-C*H*_3_), 0.13 (s, 3H, Si-C*H*_3_), 0.06 (s, 3H, Si-C*H*_3_), 0.05 (s, 3H, Si-C*H*_3_).

**^13^C-NMR** (126 MHz, DMSO-*d*_6_) δ 160.78, 155.73, 152.71, 152.23, 150.82, 150.73, 150.57, 150.43, 143.04, 125.31, 121.64, 108.73, 105.08, 104.81, 96.86, 95.25 (d, *J_C-F_* = 194 Hz), 82.19 (d, *J_C-F_* = 7.1 Hz), 81.20 (d, *J_C-F_* = 17.1 Hz), 73.47 (d, *J_C-F_* = 23.1 Hz), 62.23, 61.61, 43.98, 25.75, 25.50, 18.04, 17.65, 12.29, -4.85, -5.13, -5.48, -5.52.

**^19^F-NMR** (471 MHz, DMSO-*d*_6_) δ -198.01.

**HRMS:** *m/z* calculated for C_37_H_55_ClFN_6_O_7_Si_2_^+^ [M-H^+^] 805.3338, found 805.3401 [M-H^+^].

**TLC:** R*_f_* = 0.42 (*c*-Hex/EtOAc 1:1).

## 7.4.25 (7-(Diethylamino)-2-oxo-2*H*-chromen-4-yl)methyl (9-((2*R*,4*S*,5*R*)-4-((*tert*-butyldimethylsilyl)oxy)-5-(((*tert*-butyldimethylsilyl)oxy)methyl)tetrahydrofuran-2-yl)-2-chloro-9*H*-purin-6-yl)carbamate (TBDMS DEACM-Cladribine, 4b.2)

Following the general procedure B, **TBDMS-DEACM-cladribine** (**4b.2**) was obtained from **TBDMS-cladribine** (**4b.1**, 110 mg, 0.213 mmol) and **DEACM-CDI** (73 mg, 0.21 mmol) with LiHMDS (1 mol/L solution, 0.26 mL, 0.26 mmol) in dry THF (5 mL), as a brownish oil (152 mg, 0.193 mmol, 90%) after column chromatography (*c*-Hex/EtOAc 1:1, silica gel deactivated with 1% NEt_3_).

**^1^H-NMR** (400 MHz, DMSO-*d*_6_) δ 11.35 (s, 1H, N-*H*), 8.62 (s, 1H, Ar-*H*), 7.48 (d, *J* = 9.0 Hz, 1H, DEACM-*H*5), 6.71 (dd, *J* = 9.1, 2.5 Hz, 1H, DEACM-*H*6), 6.56 (d, *J* = 2.5 Hz, 1H, DEACM-*H*8), 6.43 – 6.29 (m, 2H, 1‘-*H* + DEACM-*H*3), 5.43 (d, *J* = 1.4 Hz, 2H, DEACM-C*H*_2_), 4.70 (q, *J* = 4.7 Hz, 1H, 3‘-*H*), 3.92 – 3.78 (m, 2H, 4‘-*H* + 5‘-*H*_a_), 3.67 (dd, *J* = 11.0, 4.2 Hz, 1H, 5‘-*H*_b_), 3.44 (q, *J* = 7.0 Hz, 4H, NC*H*_2_CH_3_), 2.95 (dt, *J* = 12.5, 6.0 Hz, 1H, 2‘-*H*_a_), 2.42 – 2.33 (m, 1H, 2‘-*H*_b_), 1.13 (t, *J* = 7.0 Hz, 6H, NCH_2_C*H*_3_), 0.91 (s, 9H, *t*Bu-C*H*_3_), 0.81 (s, 9H, *t*Bu-C*H*_3_), 0.13 (s, 6H, 2 x Si-C*H*_3_), 0.00 (s, 3H, Si-C*H*_3_), -0.04 (s, 3H, Si-C*H*_3_).

**^1^H-NMR** (400 MHz, Chloroform-*d*) δ 8.44 (s, 1H), 8.32 (s, 1H), 7.33 (d, *J* = 9.0 Hz, 1H, DEACM-*H*5), 6.58 (dd, *J* = 9.0, 2.6 Hz, 1H, DEACM-*H*6), 6.51 (d, *J* = 2.5 Hz, 1H, DEACM-*H*8), 6.43 (t, *J* = 6.3 Hz, 1H, 1‘-*H*), 6.28 (d, *J* = 1.2 Hz, 1H, DEACM-*H*3), 5.40 (d, *J* = 1.2 Hz, 2H, DEACM-C*H*_2_), 4.62 (dt, *J* = 5.8, 3.8 Hz, 1H, 3‘-*H*), 4.02 (q, *J* = 3.5 Hz, 1H, 4‘-*H*), 3.90 (dd, *J* = 11.3, 4.1 Hz, 1H, 5‘-*H*_a_), 3.77 (dd, *J* = 11.3, 3.1 Hz, 1H, 5‘-*H*_b_), 3.41 (q, *J* = 7.1 Hz, 4H, NC*H*_2_CH_3_), 2.69 – 2.58 (m, 1H, 2‘-*H*_a_), 2.50 – 2.43 (m, 1H, 2‘-*H*_b_), 1.21 (t, *J* = 7.1 Hz, 6H, NCH_2_C*H*_3_), 0.91 (s, 9H, *t*Bu-C*H*_3_), 0.90 (s, 9H, *t*Bu-C*H*_3_), 0.11 (s, 6H, 2 x Si-C*H*_3_), 0.09 (s, 3H, Si-C*H*_3_), 0.09 (s, 3H, Si-C*H*_3_).

**^13^C-NMR** (101 MHz, Chloroform-*d*) δ 161.83, 156.49, 153.98, 152.41, 150.85, 150.26, 149.53, 148.66, 142.43, 124.69, 121.59, 108.86, 107.40, 106.05, 97.99, 88.31, 85.01, 71.84, 63.50, 62.78, 44.92, 41.55, 26.09, 25.90, 18.56, 18.15, 12.58, -4.51, -4.68, -5.27, -5.35.

**HRMS:** *m/z* calculated for C_37_H_56_ClN_6_O_7_Si_2_^+^ [M-H^+^] 787.3432, found 787.3459 [M-H^+^].

**TLC:** R*_f_* = 0.62 (*c*-Hex/EtOAc 1:1).

## 7.4.26 (7-(Diethylamino)-2-oxo-2*H*-chromen-4-yl)methyl (2-chloro-9-((2*R*,3*S*,4*R*, 5*R*)-3-fluoro-4-hydroxy-5-(hydroxymethyl)tetrahydrofuran-2-yl)-9*H*-purin-6-yl) carbamate (DEACM-Clofarabine, 4a)

Following the general procedure C, **DEACM-Clofarabine** (**4a**) was obtained in the deprotection of **TBDMS-DEACM-clofarabine** (**4a.2**, 87 mg, 0.11 mmol) with TBAF (1 mol/L solution, 0.65 mL, 0.65 mmol) in THF/AcOH (2.5 mL, 4:1) for 20 h, as a bright green solid (37 mg, 0.064 mmol, 59%) after column chromatography (CH_2_Cl_2_/MeOH 0-10%, silica gel deactivated with 1% NEt_3_).

**^1^H-NMR** (500 MHz, DMSO-*d*_6_) δ 11.43 (s, 1H, N-*H*), 8.63 (d, *J* = 1.9 Hz, 1H, Ar-*H*), 7.49 (d, *J* = 9.0 Hz, 1H, DEACM-*H*5), 6.71 (dd, *J* = 9.1, 2.6 Hz, 1H, DEACM-*H*6), 6.56 (d, *J* = 2.5 Hz, 1H, DEACM-*H*8), 6.46 (dd, *J* = 13.2, 4.7 Hz, 1H, 1‘-*H*), 6.35 (s, 1H, DEACM-*H3*), 5.99 (d, *J* = 5.1 Hz, 1H, 3‘-O*H*), 5.44 (d, *J* = 1.5 Hz, 2H, DEACM-C*H*_2_), 5.30 (dt, *J* = 52.5, 4.3 Hz, 1H, 2‘-*H*), 5.11 (t, *J* = 5.7 Hz, 1H, 5‘-O*H*), 4.46 (ddd, *J* = 19.1, 9.5, 5.4 Hz, 1H, 3‘-*H*), 3.89 (q, *J* = 4.9 Hz, 1H, 4‘-*H*), 3.76 – 3.62 (m, 2H, 5‘-*H*_a_*H*_b_), 3.44 (q, *J* = 7.0 Hz, 4H, NC*H*_2_CH_3_), 1.13 (t, *J* = 7.0 Hz, 6H, NCH_2_C*H*_3_).

**^13^C-NMR** (101 MHz, DMSO-*d*_6_) δ 160.81, 155.75, 152.78, 152.18, 150.85, 150.62, 150.61, 150.45, 143.35, 143.31, 125.33, 121.55, 108.75, 105.09, 104.79, 96.87, 95.33 (d, *J_C-F_* = 193 Hz), 83.71 (d, *J_C-F_* = 5.7 Hz), 81.72 (d, *J_C-F_* = 17.1 Hz), 72.40 (d, *J_C-F_* = 23.0 Hz), 62.24, 60.27, 44.00, 12.31.

**^19^F-NMR** (471 MHz, DMSO-*d*_6_) δ -197.96.

**HRMS:** *m/z* calculated for C_25_H_27_ClFN_6_O_7_^+^ [M-H^+^] 577.1608, found 577.1601 [M-H^+^].

**TLC:** R*_f_* = 0.21 (CH_2_Cl_2_/MeOH 95:5).

## 7.4.27 (7-(Diethylamino)-2-oxo-2*H*-chromen-4-yl)methyl (2-chloro-9-((2*R*,4*S*,5*R*)-4-hydroxy-5-(hydroxymethyl)tetrahydrofuran-2-yl)-9*H*-purin-6-yl)carbamate (DEACM-Cladribine, 4b)

Following the general procedure C, **DEACM-cladribine** (**4b**) was obtained in the deprotection of **TBMDS-DEACM-cladribine** (**4b.2**, 127 mg, 0.161 mmol) with TBAF (1 mol/L solution, 1.45 mL, 1.45 mmol) in THF/AcOH (2.5 mL, 4:1) for 20 h, as a bright green solid (16 mg, 0.029 mmol, 18%) after column chromatography (CH_2_Cl_2_/MeOH 0-10%, silica gel deactivated with 1% NEt_3_).

**^1^H-NMR** (400 MHz, DMSO-*d*_6_) δ 11.36 (s, 1H, N-*H*), 8.70 (s, 1H, Ar-*H*), 7.48 (d, *J* = 9.0 Hz, 1H, DEACM-*H*5), 6.71 (dd, *J* = 9.1, 2.6 Hz, 1H, DEACM-*H*6), 6.56 (d, *J* = 2.5 Hz, 1H, DEACM-*H*8), 6.37 (t, *J* = 6.2 Hz, 1H, 1‘-*H*), 6.35 (s, 1H, DEACM-*H3*), 5.43 (s, 2H, DEACM-C*H*_2_), 5.35 (d, *J* = 4.3 Hz, 1H, 3‘-O*H*), 4.94 (t, *J* = 5.5 Hz, 1H, 5‘-O*H*), 4.42 (m, 1H, 3‘-*H*), 3.91 – 3.86 (m, 1H, 4‘-*H*), 3.66 – 3.58 (m, 1H, 5‘-*H*_a_), 3.56 – 3.49 (m, 1H, 5‘-*H*_b_), 3.44 (q, *J* = 7.0 Hz, 4H, NC*H*_2_CH_3_), 2.77 – 2.65 (m, 1H, 2‘-*H*_a_), 2.40 – 2.30 (m, 1H, 2‘-*H*_b_), 1.12 (t, *J* = 7.0 Hz, 6H, NCH_2_C*H*_3_).

**^13^C-NMR** (101 MHz, DMSO-*d*_6_) δ 160.81, 155.74, 152.77, 151.85, 150.87, 150.65, 150.46, 150.45, 143.17, 125.32, 122.26, 108.75, 105.08, 104.77, 96.87, 88.05, 83.74, 70.45, 62.19, 61.40, 44.00, 12.31.

**HRMS:** *m/z* calculated for C_25_H_28_ClN_6_O_7_^+^ [M-H^+^] 559.1703, found 559.1698 [M-H^+^].

**TLC:** R*_f_* = 0.22 (CH_2_Cl_2_/MeOH 95:5).

## 7.4.28 ((2*R*,3*R*,4*S*,5*R*)-5-(6-Amino-2-fluoro-9*H*-purin-9-yl)-3,4-bis((*tert*-butyldi-methylsilyl)oxy)tetrahydrofuran-2-yl)methanol (TBDMS-Fludarabine, 5b.1)

Following the general procedure A, **TBDMS-fludarabine** (**5b.1**) was obtained from the protection of fludarabine (0.500 g, 1.75 mmol), imidazole (0.590 g, 8.76 mmol) and TBDMSCl (1.32 g, 8.76 mmol) in 10 mL dry DMF. The solution was stirred at ambient temperature for four hours during which additional TBDMSCl (1.32 g, 8.76 mmol) was added in two parts, afterwards the solution was heated to 45 °C. The mixture was stirred overnight at ambient temperature and the reaction was stopped by the addition of 20 mL MeOH. Residual MeOH was removed under reduced pressure, CH_2_Cl_2_ and NaHCO_3_ solution were added and the aqueous phase was extracted 5 times. After the removal of solvents, the crude product was purified by column chromatography (EtOAc/*c*-Hex 3:1), the desired product was obtained as a white solid (0.470 g, 0.91 mmol, 52%)

**^1^H-NMR** (500 MHz, DMSO-*d*_6_) δ 8.10 (s, 1H, Ar-*H*), 7.81 (s, 2H, N-*H*_2_), 6.16 (d, *J* = 4.8 Hz, 1H, 1‘-*H*), 5.02 (t, *J* = 5.5 Hz, 1H, 5‘-O*H*), 4.35 (t, *J* = 4.0 Hz, 1H, 3‘-*H*), 4.30 (t, *J* = 4.4 Hz, 1H, 2‘-*H*), 3.83 (q, *J* = 5.0 Hz, 1H, 4‘-*H*), 3.69 – 3.61 (m, 2H, 5‘-*H*_a_*H*_b_), 0.91 (s, 9H, *t*Bu-C*H*_3_), 0.61 (s, 9H, *t*Bu-C*H*_3_), 0.15 (s, 3H, Si-C*H*_3_), 0.14 (s, 3H, Si-C*H*_3_), -0.03 (s, 3H, Si-C*H*_3_), -0.37 (s, 3H, Si-C*H*_3_).

**^13^C-NMR** (126 MHz, DMSO-*d*_6_) δ 158.59 (d, *J_C-F_* = 204 Hz), 157.48 (d, *J_C-F_* = 21.4 Hz), 150.42 (d, *J_C-F_* = 20.4 Hz), 140.26 (d, *J_C-F_* = 2.7 Hz), 116.71 (d, *J_C-F_* = 4.7 Hz), 84.90, 83.69, 76.83, 76.45, 60.48, 25.65, 25.23, 17.56, 17.17, ‑4.51, ‑4.69, -5.26, -5.75.

**^19^F-NMR** (471 MHz, DMSO-*d*_6_) δ -52.27.

**TLC:** R*_f_* = 0.69 (EtOAc/*c*-Hex 3:1).

**HRMS:** *m/z* calculated for C_22_H_41_FN_5_O_4_Si_2_^+^ [M-H^+^] 514.2676, found 514.2688 [M-H^+^].

## 7.4.29 ((2*R*,3*R*,4*S*,5*R*)-5-(6-Amino-2-fluoro-9*H*-purin-9-yl)-3,4-bis((*tert*-butyldi-methylsilyl)oxy)tetrahydrofuran-2-yl)methyl 1*H*-imidazole-1-carboxylate (TBDMS-CDI-Fludarabine, 5b.2 )

**TBDMS-fludarabine** (**5b.1**, 0.300 g, 0.584 mmol) and 1,1‘-carbonyldiimidazole (0.116 g, 0.717 mmol) were dissolved in 16 mL of dry CH_2_Cl_2_ in a microwave vessel and heated to 50 °C for 30 minutes in a microwave reactor. 1,1‘-carbonyldiimidazole (0.116 g, 0.717 mmol) was added and the resulting solution was heated for 90 minutes in a microwave reactor. Water, NaHCO_3_ solution and CH_2_Cl_2_ were added and the aqueous phase was extracted 3 times with CH_2_Cl_2_. The crude product (off-white powder, quant.) was used without further purification in the next step.

**^1^H-NMR** (500 MHz, DMSO-*d*_6_) δ 8.31 (t, *J* = 1.1 Hz, 1H, Imidazole-*H*), 8.20 (s, 1H, Ar-*H*), 7.83 (br s, 2H, N-*H*_2_), 7.63 (t, *J* = 1.5 Hz, 1H, Imidazole-*H*), 7.08 (dd, *J* = 1.7, 0.9 Hz, 1H, Imidazole-*H*), 6.24 (d, *J* = 4.6 Hz, 1H, 1‘-*H*), 4.79 – 4.66 (m, 2H, 5‘-*H*_a_*H*_b_), 4.54 (t, *J* = 3.7 Hz, 1H, 3‘-*H*), 4.36 – 4.29 (m, 1H, 2‘-*H*), 4.24 – 4.18 (m, 1H, 4‘-*H*), 0.92 (s, 9H, *t*Bu-C*H*_3_), 0.66 (s, 9H, *t*Bu-C*H*_3_), 0.17 (s, 6H, 2 x Si-C*H*_3_), -0.02 (s, 3H, Si-C*H*_3_), -0.41 (s, 3H, Si-C*H*_3_).

**^13^C-NMR** (126 MHz, DMSO-*d*_6_) δ 158.61 (d, *J_C-F_* = 204 Hz), 157.50 (d, *J_C-F_* = 21.5 Hz), 150.32 (d, *J_C-F_* = 20.6 Hz), 148.34, 140.54 (d, *J_C-F_* = 2.9 Hz), 137.35, 130.36, 117.56, 116.79 (d, *J_C-F_* = 4.7 Hz), 84.16, 81.51, 76.66, 76.25, 66.82, 25.61, 25.30, 17.50, 17.23, -4.59, -4.88, -5.43, -5.83.

**^19^F-NMR** (471 MHz, DMSO-*d*_6_) δ -52.19.

**TLC:** R*_f_* = 0.11 (EtOAc/*c*-Hex 1:1).

**HRMS:** *m/z* calculated for C_26_H_43_FN_7_O_5_Si_2_^+^ [M-H^+^] 608.2843, found 608.2855 [M-H^+^].

## 7.4.30 ((2*R*,3*R*,4*S*,5*R*)-5-(6-Amino-2-fluoro-9*H*-purin-9-yl)-3,4-bis((*tert*-butyldime-thylsilyl)oxy)tetrahydrofuran-2-yl)methyl ((7-(diethylamino)-2-oxo-2*H*-chrom-en-4-yl)methyl) carbonate (TBDMS-DEACM-Fludarabine, 5b.3)

**TBDMS-CDI-fludarabine** (**5b.2**, 0.350 g, 0.576 mmol), **DEACM-OH** (0.240 g, 0.971 mmol) and 4-dimethylaminopyridine (0.148 g, 1.21 mmol) were dissolved in 18 mL of dry CH_2_Cl_2_ and heated to 60 °C in a microwave reactor. After 6 hours at 60 °C the solvent was removed under reduced pressure and the crude product was purified by column chromatography (EtOAc/*c*-Hex 1:1). The desired product was obtained as a yellow solid (0.200 g, 0.254 mmol, 44%).

**^1^H-NMR** (500 MHz, DMSO-*d*_6_) δ 8.11 (s, 1H, Ar-*H*), 7.83 (br s, 2H, N-*H*_2_), 7.45 (d, *J* = 9.0 Hz, 1H, DEACM-*H*5), 6.65 (dd, *J* = 9.0, 2.6 Hz, 1H, DEACM-*H*6), 6.54 (d, *J* = 2.5 Hz, 1H, DEACM-*H*8), 6.22 (d, *J* = 4.4 Hz, 1H, 1‘-*H*), 5.97 (s, 1H, DEACM-*H*3), 5.50 – 5.29 (m, 2H, DEACM-C*H*_2_), 4.60 – 4.53 (m, 1H, 5‘-*H*_a_), 4.44 (dd, *J* = 11.5, 4.4 Hz, 1H, 5‘-*H*_b_), 4.39 (t, *J* = 3.4 Hz, 1H, 3‘-*H*), 4.31 – 4.28 (m, 1H, 2‘-*H*), 4.14 – 4.06 (m, 1H, 4‘-*H*), 3.42 (q, *J* = 7.1 Hz, 4H, NC*H*_2_CH_3_), 1.11 (t, *J* = 7.0 Hz, 6H, NCH_2_C*H*_3_), 0.90 (s, 9H, *t*Bu-C*H*_3_), 0.64 (s, 9H, *t*Bu-C*H*_3_), 0.14 (s, 6H, 2 x Si-C*H*_3_), -0.04 (s, 3H, Si-C*H*_3_), -0.42 (s, 3H, Si-C*H*_3_).

**^13^C-NMR** (126 MHz, DMSO-*d*_6_) δ 160.45, 158.61 (d, *J_C-F_* = 204 Hz), 157.52 (d, *J_C-F_* = 21.4 Hz), 155.86, 154.01, 150.49, 150.26 (d, *J_C-F_* = 20.3 Hz), 149.63, 140.26, 125.55, 116.77 (d, *J_C-F_* = 4.8 Hz), 108.72, 105.60, 105.12, 96.87, 84.34, 81.91, 76.97, 76.26, 67.33, 64.89, 43.97, 25.59, 25.26, 17.52, 17.18, 12.26, ‑4.67, ‑4.91, ‑5.44, ‑5.88.

**^19^F-NMR** (471 MHz, DMSO-*d*_6_) δ -52.11.

**HRMS:** *m/z* calculated for C_37_H_56_FN_6_O_8_Si_2_^+^ [M-H^+^] 787.3677, found 787.3710 [M-H^+^].

**TLC:** R*_f_* = 0.42 (EtOAc/*c*-Hex 1:1).

## 7.4.31 **((2*R*,3*S*,4*S*,5*R*)-5-(6-Amino-2-fluoro-9*H*-purin-9-yl)-3,4-dihydroxytetra-hydrofuran-2-yl)methyl ((7-(diethylamino)-2-oxo-2*H*-chromen-4-yl)methyl) carbonate (DEACM-Fludarabine, 5b)**

Following the general procedure C, **TBDMS-DEACM-fludarabine** (**5b.3**, 0.150 g, 0.191 mmol) was dissolved in 6 mL THF/AcOH (4:1) and TBAF (1 mol/L, 0.95 mL, 0.95 mmol) was added. After one day additional TBAF (1 mol/L, 0.95 mL, 0.95 mmol) was added, the solution was stirred for another day at ambient temperature. Workup was conducted as described previously. The crude product was purified by column chromatography (CH_2_Cl_2_/MeOH 100:5, silica deactivated with 1% NEt_3_), compound **5b** was obtained as a yellow solid (0.088 g, 0.16 mmol, 83%).

**^1^H-NMR** (500 MHz, DMSO-*d*_6_) δ 8.11 (s, 1H, Ar-*H*), 7.80 (br s, 2H, N-*H*_2_), 7.44 (d, *J* = 9.0 Hz, 1H, DEACM-*H*5), 6.66 (dd, *J* = 9.1, 2.6 Hz, 1H, DEACM-*H*6), 6.54 (d, *J* = 2.6 Hz, 1H, DEACM-*H*8), 6.18 (d, *J* = 4.5 Hz, 1H, 1‘-*H*), 5.98 (s, 1H, DEACM-*H3*), 5.81 (d, *J* = 4.6 Hz, 1H, 2‘-O*H*), 5.76 (d, *J* = 4.3 Hz, 1H, 3‘-O*H*), 5.44 – 5.26 (m, 2H, DEACM-C*H*_2_), 4.59 – 4.51 (m, 1H, 5‘-*H*_a_), 4.47 – 4.39 (m, 1H, 5‘-*H*_b_), 4.24 – 4.11 (m, 2H, 2‘-*H* + 3‘-*H*), 4.10 – 3.96 (m, 1H, 4‘-*H*), 3.42 (q, *J* = 7.0 Hz, 4H, NC*H*_2_CH_3_), 1.10 (t, *J* = 7.0 Hz, 6H, NCH_2_C*H*_3_).

**^13^C-NMR** (126 MHz, DMSO-*d*_6_) δ 160.50, 158.58 (d, *J_C-F_* = 204 Hz), 157.49 (d, *J_C-F_* = 21.3 Hz), 155.84, 154.06, 150.61 (d, *J_C-F_* ≈ 20 Hz), 150.51, 149.74, 140.60 (d, *J_C-F_* = 3.6 Hz), 125.53, 116.66 (d, *J_C-F_* = 5.1 Hz), 108.78, 105.46, 105.12, 96.86, 84.03, 80.96, 75.40, 74.91, 68.02, 64.77, 43.98, 12.28.

**^19^F-NMR** (471 MHz, DMSO-*d*_6_) δ -52.32.

**HRMS:** *m/z* calculated for C_25_H_28_FN_6_O_8_^+^ [M-H^+^] 559.1947, found 559.1948 [M-H^+^].

**TLC:** R*_f_* = 0.08 (CH_2_Cl_2_/MeOH 100:5).

## 7.4.32 (*S*)-Bis((7-(Diethylamino)-2-oxo-2*H*-chromen-4-yl)methyl) 2-(4-(((2,4-diamino‑pteridin‑6‑yl)methyl)(methyl)amino)benzamido)pentanedioate (DEACM‑Methotrexate, 8)

Following general procedure D with methotrexate (0.198 g, 0.436 mmol), **DEACM-OH** (0.151 g, 0.610 mmol), 4-dimethylaminopyridine (0.064 g, 0.52 mmol) and *N*-ethyl-*N*’-(3-dimethylaminopropyl)carbodiimide hydrochloride (0.092 g, 0.48 mmol) in 5 mL of dry CH_2_Cl_2_. The crude product was purified by column chromatography (CH_2_Cl_2_/MeOH 10:1, 1% AcOH). The desired product (**8**) was obtained as a yellow solid (0.061 g, 0.067 mmol, 15%).

**^1^H-NMR** (500 MHz, DMSO-*d*_6_) δ 8.58 (s, 1H, Ar-*H*), 8.52 (d, *J* = 7.3 Hz, 1H, CON-*H*), 7.75 (d, *J* = 9.0 Hz, 2H, Ph-*H*), 7.65 (s, 1H, N4-*H*_a_H_b_), 7.44 – 7.38 (m, 3H, 2 x DEACM-*H*5 + 4N-H_a_*H*_b_), 6.83 (d, *J* = 9.0 Hz, 2H, Ph-*H*), 6.64 (dd, *J* = 9.1, 2.5 Hz, 1H, DEACM-*H*6), 6.60 (s, 2H, N2-*H*_2_), 6.52 (d, *J* = 2.5 Hz, 1H, DEACM-*H*8), 6.51 – 6.49 (m, 2H, DEACM-*H*6 + DEACM-*H*8), 6.06 (s, 1H, DEACM-*H*3), 5.99 (s, 1H, DEACM-*H*3), 5.32 – 5.27 (m, 2H, DEACM-C*H*_2_), 5.26 (s, 2H, DEACM-C*H*_2_), 4.77 (s, 2H, C*H*_2_NCH_3_), 4.60 – 4.46 (m, 1H, C*H*CH_2_CH_2_CO_2_), 3.40 (q, *J* = 7.0 Hz, 4H, NC*H*_2_CH_3_), 3.35 (q, *J* = 7.1 Hz, 4H, NC*H*_2_CH_3_), 3.21 (s, 3H, CH_2_NC*H*_3_), 2.63 (t, *J* = 7.4 Hz, 2H, CHCH_2_C*H*_2_CO_2_), 2.26 – 2.17 (m, 1H, CHC*H*_2_CH_2_CO_2_), 2.16 – 2.07 (m, 1H, CHC*H*_2_CH_2_CO_2_), 1.10 (t, *J* = 7.0 Hz, 6H, NCH_2_C*H*_3_), 1.07 (t, *J* = 7.0 Hz, 6H, NCH_2_C*H*_3_).

**^13^C-NMR** (126 MHz, DMSO-*d*_6_) δ 171.88, 171.59, 166.66, 162.87, 162.70, 160.59, 160.57, 155.78, 155.75, 155.23, 151.09, 150.42, 150.37, 150.34, 150.16, 149.19, 145.90, 129.04, 125.61, 125.45, 121.40, 120.62, 111.00, 108.71, 108.61, 105.55, 105.31, 105.27, 96.82, 96.77, 62.04, 61.33, 54.90, 54.82, 52.05, 43.95, 43.90, 39.08, 30.01, 25.56, 25.10, 12.26, 12.23.

**HRMS:** *m/z* calculated for C_48_H_53_N_10_O_9_^+^ [M-H^+^] 913.39915, found 913.39866 [M-H^+^].

**TLC:** R*_f_* = 0.41 (CH_2_Cl_2_/MeOH 10:1).

## 7.4.33 (*S*)-Bis((7-(Diethylamino)-2-oxo-2*H*-chromen-4-yl)methyl) 2-(5-(methyl((2-methyl-4-oxo-1,4-dihydroquinazolin-6-yl)methyl)amino)thiophene-2-carbox-amido)pentanedioate (DEACM-Raltitrexed, 9)

Following general procedure D with raltitrexed (75 mg, 164 µmol), **DEACM-OH** (81 mg, 327 µmol, 2 equiv.), DMAP (40 mg, 0.33 mmol) and *N*-ethyl-*N*’-(3-dimethylaminopropyl)carbodiimide hydrochloride (63 mg, 0.33 mmol) in 2 mL dry CH_2_Cl_2_ (in this case stirred for two days). Purified by flash chromatography (CH_2_Cl_2_/MeOH 0 – 15%), the desired product was obtained as a green brownish solid (114 mg, 0.124 mmol, 76%).

**^1^H-NMR** (600 MHz, DMSO-*d*_6_) δ 12.19 (s, 1H, quinazoline-N*H*), 8.40 (d, *J* = 7.4 Hz, 1H, CON*H*), 7.94 (d, *J* = 2.1 Hz, 1H, quinazoline-*H*5), 7.65 (dd, *J* = 8.4, 2.1 Hz, 1H, quinazoline-*H*7), 7.57 (d, *J* = 4.2 Hz, 1H, thiophene-*H*3), 7.55 (d, *J* = 8.3 Hz, 1H, quinazoline-*H*8), 7.42 (d, *J* = 9.0 Hz, 2H, 2 x DEACM-*H*5), 6.65 (dd, *J* = 9.1, 2.6 Hz, 1H, DEACM-*H*6), 6.56 (dd, *J* = 9.1, 2.6 Hz, 1H, DEACM-*H*6), 6.52 (d, *J* = 2.5 Hz, 1H, DEACM-*H*8), 6.51 (d, *J* = 2.5 Hz, 1H, DEACM-*H*8), 6.05 (d, *J* = 1.3 Hz, 1H, DEACM-*H*3), 6.01 – 5.98 (m, 2H, thiophene-*H*4 + DEACM-*H*3) 5.36 – 5.26 (m, 2H, DEACM-C*H*_2_), 5.25 – 5.22 (m, 2H, DEACM-C*H*_2_), 4.64 (s, 2H, quinazoline-C*H*_2_), 4.57 – 4.46 (m, 1H, C*H*CH_2_CH_2_CO_2_), 3.39 (m, 8H, NC*H*_2_CH_3_), 3.04 (s, 3H, N-C*H*_3_), 2.63 (t, *J* = 7.5 Hz, 2H, CHCH_2_C*H*_2_CO_2_), 2.33 (s, 3H, C*H*_3_), 2.23 – 2.15 (m, 1H, CHC*H*_2_CH_2_CO_2_), 2.12 – 2.04 (m, 1H, CHC*H*_2_CH_2_CO_2_), 1.09 (m, 12H, NCH_2_C*H*_3_).

**^13^C-NMR** (151 MHz, DMSO-*d*_6_) δ 171.82, 171.51, 162.14, 162.01, 161.56, 160.57, 160.54, 155.76, 154.11, 150.42, 150.38, 150.31, 150.11, 148.29, 134.97, 133.46, 130.61, 126.95, 125.58, 125.41, 123.89, 120.52, 120.02, 108.71, 108.61, 105.51, 105.30, 105.28, 102.08, 96.82, 96.78, 62.05, 61.33, 57.28, 51.81, 43.94, 43.92, 40.11, 29.97, 25.71, 21.36, 12.25.

**HRMS:** *m/z* calculated for C_49_H_53_N_6_O_10_S^+^ [M-H^+^] 917.3538, found 917.3572 [M-H^+^].

**TLC:** R*_f_* = 0.26 (CH_2_Cl_2_/MeOH 95:5).

## 7.4.34 (*S*)-Bis((7-(Diethylamino)-2-oxo-2*H*-chromen-4-yl)methyl) 2-(4-(2-(2-amino-4-oxo-4,7-dihydro-3*H*-pyrrolo[2,3-*d*]pyrimidin-5‑yl)ethyl)benzamido)pentanedi-oate (DEACM-Pemetrexed, 10)

Following general procedure D with pemetrexed (0.100 g, 0.234 mmol), **DEACM-OH** (0.116 g, 0.468 mmol), 4-dimethylaminopyridine (0.057 g, 0.47 mmol) and *N*-ethyl-*N*’-(3-dimethylaminopropyl)carbodiimide hydrochloride (0.090 g, 0.47 mmol) in 5 mL of dry CH_2_Cl_2_ (starting materials dried under reduced pressure for three hours). The crude product was purified by column chromatography (CH_2_Cl_2_/MeOH 10:1). The desired product was obtained as a yellow solid (0.056 g, 0.063 mmol, 27%).

**^1^H-NMR** (500 MHz, DMSO-*d*_6_) δ 10.61 (d, *J* = 2.2 Hz, 1H, pyrrole N-*H*), 10.14 (s, 1H, pyrimidine N-*H*), 8.84 (d, *J* = 7.2 Hz, 1H, CON*H*), 7.80 (d, *J* = 8.2 Hz, 2H, Ph-*H*), 7.43 (d, *J* = 9.0 Hz, 1H, DEACM-*H*5), 7.42 (d, *J* = 9.0 Hz, 1H, DEACM-*H*5), 7.29 (d, *J* = 8.2 Hz, 2H, Ph-*H*), 6.65 (dd, *J* = 9.1, 2.6 Hz, 1H, DEACM-*H*6), 6.55 (dd, *J* = 9.0, 2.6 Hz, 1H, DEACM-*H*6), 6.53 (d, *J* = 2.5 Hz, 1H, DEACM-*H*8), 6.51 (d, *J* = 2.5 Hz, 1H, DEACM-*H*8), 6.31 (d, *J* = 2.1 Hz, 1H, Ar-*H*), 6.07 (s, 1H, DEACM-*H*3), 6.01 (s, 1H, DEACM-*H*3), 5.99 (s, 2H, N-*H*_2_) 5.39 – 5.28 (m, 2H, DEACM-C*H*_2_), 5.26 (s, 2H, DEACM-C*H*_2_), 4.64 – 4.56 (m, 1H, C*H*CH_2_CH_2_CO_2_), 3.47 – 3.36 (m, 8H, NC*H*_2_CH_3_), 3.04 – 2.91 (m, 2H, CH_2_C*H*_2_), 2.92 – 2.80 (m, 2H, CH_2_C*H*_2_), 2.71 – 2.61 (m, 2H, CHCH_2_C*H*_2_CO_2_), 2.32 – 2.06 (m, 2H, CHC*H*_2_CH_2_CO_2_), 1.12 – 1.07 (m, 12H, NCH_2_C*H*_3_).

**^13^C-NMR** (126 MHz, DMSO-*d*_6_) δ 171.88, 171.35, 166.89, 160.62, 160.60, 159.28, 155.80, 155.79, 152.20, 151.35, 150.45, 150.40, 150.38, 150.17, 146.45, 130.93, 128.20, 127.48, 125.60, 125.48, 117.64, 113.41, 108.74, 108.65, 105.51, 105.30, 105.28, 105.25, 98.74, 96.84, 96.79, 62.10, 61.36, 52.15, 43.97, 43.95, 36.18, 30.00, 28.00, 25.50, 12.28.

**HRMS:** *m/z* calculated for C_48_H_52_N_7_O_10_^+^ [M-H^+^] 886.3770, found 886.3812 [M-H^+^].

**TLC:** R*_f_* = 0.41 (CH_2_Cl_2_/MeOH 10:1).

## 7.4.35 1‑(Pyridin‑4‑yl)ethyl(1‑((2*R*,4*R*,5*R*)‑3,3‑difluoro‑4‑hydroxy‑5‑(hydroxymethyl)tetrahydrofuran‑2‑yl)‑2‑oxo‑1,2‑dihydropyrimidin‑4‑yl)carbamate (Gemcitabine-Pyridine, 11a)

A dried schlenk-flask was equipped with gemcitabine (0.095 g, 0.364 mmol) under the exclusion of air. 5 mL of dry THF were added and the resulting solution was cooled to -78 °C. Afterwards LiHMDS (1 mol/L in THF, 0.35 mL, 0.35 mmol) was added, the cooling bath was removed and the resulting solution stirred at ambient temperature for 30 minutes. 4-Nitrophenyl(1-(pyridin-4-yl)ethyl) carbonate^[5]^ (0.105 g, 0.364 mmol) was added and the mixture was stirred overnight at ambient temperature. The solvent was removed under reduced pressure and the crude product was purified by column chromatography (CH_2_Cl_2_/MeOH 98:2 - CH_2_Cl_2_/MeOH 9:1). The desired product was obtained as a white solid (0.060 g, 0.15 mmol, 40%).

**^1^H-NMR** (500 MHz, DMSO-*d*_6_) δ 11.08 (s, 1H, N-*H*), 8.60 – 8.55 (m, 2H, Pyridine-*H*), 8.22 (d, *J* = 7.6 Hz, 1H, Cytidine-*H*), 7.50 – 7.32 (m, 2H, Pyridine-*H*), 7.04 (d, *J* = 7.6 Hz, 1H, Cytidine-*H*), 6.31 (dd, *J* = 6.5, 1.5 Hz, 1H, 3‘-O*H*), 6.16 (t, *J* = 7.5 Hz, 1H, 1‘-*H*), 5.82 (q, *J* = 6.5 Hz, 1H, CH_3_-C*H*), 5.29 (td, *J* = 5.5, 1.9 Hz, 1H, 5‘-O*H*), 4.22 – 4.12 (m, 1H, 3‘-*H*), 3.93 – 3.86 (m, 1H, 4‘-*H*), 3.83 – 3.75 (m, 1H, 5‘-*H_a_*), 3.70 – 3.61 (m, 1H, 5‘-*H*_b_), 1.52 (d, *J* = 6.7 Hz, 3H, C*H*_3_-CH).

**^13^C-NMR** (126 MHz, DMSO-*d*_6_) δ 163.31, 153.98, 152.28, 150.26, 150.22, 149.86, 144.49, 122.94 (t, *J_C‑F_* = 258 Hz), 120.39, 120.36, 94.87, 84.10 (m), 80.99, 71.86, 68.38 (m), 58.78, 21.91.

**^19^F-NMR** (471 MHz, DMSO-*d*_6_) δ -116.91.

**HRMS:** *m/z* calculated for C_17_H_19_F_2_N_4_O_6_^+^ [M-H^+^] 413.1267, found 413.1276 [M-H^+^].

**TLC:** R*_f_* = 0.24 CH_2_Cl_2_/MeOH 10:1.

## 7.4.36 4-(1-(((1-((2*R*,4*R*,5*R*)-3,3-Difluoro-4-hydroxy-5-(hydroxymethyl)tetrahydro-furan‑2‑yl)‑2‑oxo‑1,2‑dihydropyrimidin‑4‑yl)carbamoyl)oxy)ethyl)‑1‑(2,4‑dinitro-phenyl)pyridin-1-ium triflate (Gemcitabine-zincke salt, 11b)

**Gemcitabine-pyridine** (**11a**, 0.173 g, 0.420 mmol) and 2,4-dinitrophenyl triflate^[19]^ (0.146 g, 0.462 mmol) were transferred to a round bottom flask (25 mL) and flushed with argon, 0.6 mL of dry MeCN was added. The resulting mixture was stirred overnight at ambient temperature. Dry Et_2_O (1.7 mL) was added and the product was left to precipitate while stirring for 2 hours. The supernatant was removed and the precipitate was washed with 3.4 mL of dry Et_2_O. The crude product (0.300 g) was used in the following step without further purification.

**HRMS:** calculated for C_23_H_21_F_2_N_6_O_10_^+^ [M^+^] 579.1282 found 579.1303 [M^+^].

## 7.4.37 2-((1*E*,3*Z*,5*E*,7*E*)-4-(1-(((1-((2*R*,4*R*,5*R*)-3,3-Difluoro-4-hydroxy-5-(hydroxy-methyl) tetrahydrofuran-2-yl)-2-oxo-1,2-dihydropyrimidin-4‑yl)carbamoyl)oxy) ethyl)-7-(1,3,3-trimethylindolin-2-ylidene)hepta-1,3,5-trien-1-yl)-1,3,3-trimethyl-3*H*-indol-1-ium (Gem-Cy-tabine, 11)

Gemcitabine zincke salt (**11b**, 0.193 g, 0.265 mmol), 1,2,3,3-tetramethyl-3*H*-indolium iodide (0.240 g, 0.795 mmol) and AcOK (0.156 g, 1.59 mmol) were dissolved in 2.4 mL of dry MeCN. The resulting dark purple solution turned green quickly and was stirred overnight at ambient temperature covered from light. The solvent was removed under reduced pressure the residue was washed two times with 0.8 mL and one time with 1.6 mL of dry Et_2_O. The crude product was purified two times by column chromatography (1.: CH_2_Cl_2_/MeOH 25:1 - 10:1; 2.: CH_2_Cl_2_/MeOH 10:1). The resulting green residue was washed extensively with small aliquots of water and Et_2_O. The crude product was then dissolved in CH_2_Cl_2_ (≈ 0.5 mL/ 5 mg crude product) and precipitated by the addition of Et_2_O (≈ 10 mL/ 5 mg crude product) overnight at 4 °C, this process had to be repeated several times. The desired product was obtained as a green solid (0.013 g, 0.015 mmol, 5.6%).

**^1^H-NMR** (500 MHz, Methanol-*d*_4_, mixture of diastereomers) δ 8.30 (2 x d, *J* = 7.8, 1H, Cytidine-*H*), 8.22 (2 x dd, *J* = 13.5, 13.4, 2H, Cy7-*H*), 7.49 (d, *J* = 7.4 Hz, 2H, Ar-*H*), 7.42 (td, *J* = 7.7, 1.2 Hz, 2H, Ar-*H*), 7.32 – 7.23 (m, 5H, Ar-*H* + Cytidine-*H*), 6.68 (2 x d, *J* = 13.4, 2H, Cy7-*H*), 6.40 (d, *J* = 13.3 Hz, 2H, Cy7-*H*), 6.28 – 6.20 (m, 1H, 1‘-*H*), 6.10 (q, *J* = 6.8 Hz, 1H, CH_3_-C*H*), 4.32 – 4.22 (m, 1H), 3.99 – 3.92 (m, 2H), 3.82 – 3.76 (m, 1H), 3.65 (s, 6H, N-C*H*_3_), 1.73 (s, 6H, C-C*H*_3_), 1.72 (s, 6H, C-C*H*_3_), 1.69 (2 x d, *J* = 6.8, 3H, C*H*_3_-CH).

**^1^H-NMR** (500 MHz, DMSO-*d*_6_, mixture of diastereomers) δ 11.06 (2 x s, 1H, N-*H*), 8.20 (d, *J* = 7.7 Hz, 1H, Cytidine-*H*), 8.03 (dd, *J* = 13.5, 13.2 Hz, 2H, Cy7-*H*), 7.59 (d, *J* = 7.4 Hz, 2H, Ar-*H*), 7.46 – 7.37 (m, 4H, Ar-*H*), 7.25 (td, *J* = 7.0, 1.8 Hz, 2H, Ar-*H*), 7.02 (2 x d, *J* = 7.6 Hz, 1H, Cytidine-*H*), 6.68 (d, *J* = 13.4 Hz, 2H, Cy7-*H*), 6.42 (d, *J* = 13.4 Hz, 2H, Cy7-*H*), 6.30 (2 x d, *J* = 6.5 Hz, 1H, 3‘-O*H*), 6.18 – 6.13 (m, 1H, 1‘-*H*), 6.08 (q, 1H, CH_3_-C*H*), 5.28 – 5.22 (m, 1H, 5‘-O*H*), 4.21 – 4.09 (m, 1H, 3‘-*H*), 3.91 – 3.85 (m, 1H, 4‘-*H*), 3.82 – 3.75 (m, 1H, 5‘-*H*_a_), 3.68 – 3.59 (m, 7H, 5‘-*H*_b_ and N-C*H*_3_), 1.65 (2 x s, 12H, C-C*H*_3_), 1.59 (2 x d, *J* = 6.7 Hz, 3H, C*H*_3_-CH).

**^13^C-NMR** (126 MHz, Methanol-*d*_4_) δ 173.84, 165.26, 161.25, 161.22, 157.35, 153.80, 145.86, 145.20, 145.16, 144.46, 142.52, 129.81, 126.23, 123.92 (t, *J_C‑F_* = 259 Hz), 123.30, 121.74, 111.83, 106.28, 96.94, 86.41 (m), 82.84, 72.46, 70.24 (m), 60.27, 50.40, 31.70, 28.27, 28.21, 21.64.

**^19^F-NMR** (471 MHz, Methanol-*d*_4_) δ -119.32.

**HRMS:** *m/z* calculated for C_41_H_46_F_2_N_5_O_6_^+^ [M^+^] 742.3411, found 742.3443 [M^+^].

**TLC:** R*_f_* = 0.28 CH_2_Cl_2_/MeOH 10:1.

# 8 Appendix

## 8.3 NMR Spectra

### 8.3.1 6MP-DEACM, Compound 1

Figure S38: 500 MHz ^1^H-NMR spectrum of compound **1** in DMSO-*d_6_*

Figure S39: 126 MHz ^13^C-NMR spectrum of compound **1** in DMSO-*d_6_*

### 8.3.2 Tegafur-Triazole, Compound 2b.1

Figure S40: 500 MHz ^1^H-NMR spectrum of compound **2b.1** in DMSO-*d_6_*

Figure S41: 126 MHz ^13^C-NMR spectrum of compound **2b.1** in DMSO-*d_6_*

Figure S42: 471 MHz ^19^F-NMR spectrum of compound **2b.1** in DMSO-*d_6_*

### 8.3.3 DEACM-Tegafur, Compound 2b

Figure S43: 500 MHz ^1^H-NMR spectrum of compound **2b** in DMSO-*d_6_*

Figure S44: 126 MHz ^13^C-NMR spectrum of compound **2b** in DMSO-*d_6_*

Figure S45: 471 MHz ^19^F-NMR spectrum of compound **2b** in DMSO-*d_6_*

### 8.3.4 DEACM-5FU, Compound 2a

Figure S46: 500 MHz ^1^H-NMR spectrum of compound **2a** in DMSO-*d_6_*

Figure S47: 126 MHz ^13^C-NMR spectrum of compound **2a** in DMSO-*d_6_*

Figure S48: 471 MHz ^19^F-NMR spectrum of compound **2a** in DMSO-*d_6_*

### 8.3.5 TBDMS-DEACM-Floxuridine, Compound 2c.2

Figure S49: 500 MHz ^1^H-NMR spectrum of compound **2c.2** in DMSO-*d_6_*

Figure S50: 126 MHz ^13^C-NMR spectrum of compound **2c.2** in DMSO-*d_6_*

Figure S51: 471 MHz ^19^F-NMR spectrum of compound **2c.2** in DMSO-*d_6_*

### 8.3.6 DEACM-Floxuridine, Compound 2c

Figure S52: 500 MHz ^1^H-NMR spectrum of compound **2c** in DMSO-*d_6_*

Figure S53: 126 MHz ^13^C-NMR spectrum of compound **2c** in DMSO-*d_6_*

Figure S54: 471 MHz ^19^F-NMR spectrum of compound **2c** in DMSO-*d_6_*

### 8.3.7 TBDMS-DEACM-Bn-Floxuridine, Compound 6.2

Figure S 55: 500 MHz ^1^H-NMR spectrum of compound **6.2** in DMSO-*d_6_*

Figure S56: 126 MHz ^13^C-NMR spectrum of compound **6.2** in DMSO-*d_6_*

Figure S57: 471 MHz ^19^F-NMR spectrum of compound **6.2** in DMSO-*d_6_*

### 8.3.8 DEACM-Bn-Floxuridine, Compound 6

Figure S58: 500 MHz ^1^H-NMR spectrum of compound **6** in DMSO-*d_6_*

Figure S59: 126 MHz ^13^C-NMR spectrum of compound **6** in DMSO-*d_6_*

Figure S60: 471 MHz ^19^F-NMR spectrum of compound **6** in DMSO-*d_6_*

### 8.3.9 5’-DEACM-Floxuridine, Compound 5a

Figure S61: 500 MHz ^1^H-NMR spectrum of compound **5a** in DMSO-*d_6_*

Figure S62: 126 MHz ^13^C-NMR spectrum of compound **5a** in DMSO-*d_6_*

Figure S63: 471 MHz ^19^F-NMR spectrum of compound **5a** in DMSO-*d_6_*

### 8.3.10 TBDMS-Gemcitabine, Compound 3a.1

Figure S64: 500 MHz ^1^H-NMR spectrum of compound **3a.1** in DMSO-*d_6_*

Figure S65: 126 MHz ^13^C-NMR spectrum of compound **3a.1** in DMSO-*d_6_*

Figure S66: 471 MHz ^19^F-NMR spectrum of compound **3a.1** in DMSO-*d_6_*

### 8.3.11 TBDMS-Decitabine, Compound 3c.1

Figure S67: 500 MHz ^1^H-NMR spectrum of compound **3c.1** in DMSO-*d_6_*

Figure S68: 126 MHz ^13^C-NMR spectrum of compound **3c.1** in DMSO-*d_6_*

### 8.3.12 TBDMS-DEACM-Gemcitabine, Compound 3a.2

Figure S69: 500 MHz ^1^H-NMR spectrum of compound **3a.2** in DMSO-*d_6_*

Figure S70: 126 MHz ^13^C-NMR spectrum of compound **3a.2** in DMSO-*d_6_*

Figure S71: 471 MHz ^19^F-NMR spectrum of compound **3a.2** in DMSO-*d_6_*

### 8.3.13 DEACM-Bn-CDI

Figure S72: 400 MHz ^1^H-NMR spectrum of compound **DEACM-Bn-CDI** in DMSO-*d_6_*

Figure S73: 101 MHz ^13^C-NMR spectrum of compound **DEACM-Bn-CDI** in DMSO-*d_6_*

### 8.3.14 TBDMS-DEACM-Bn-Gemcitabine, Compound 7.1

Figure S74: 500 MHz ^1^H-NMR spectrum of compound **7.1** in DMSO-*d_6_*

Figure S75: 126 MHz ^13^C-NMR spectrum of compound **7.1** in DMSO-*d_6_*

Figure S76: 471 MHz ^19^F-NMR spectrum of compound **7.1** in DMSO-*d_6_*

### 8.3.15 TBDMS-DEACM-Decitabine, Compound 3c.2

Figure S77: 400 MHz ^1^H-NMR spectrum of compound **3c.2** in DMSO-*d_6_*

Figure S78: 101 MHz ^13^C-NMR spectrum of compound **3c.2** in DMSO-*d_6_*

### 8.3.16 DEACM-Gemcitabine, Compound 3a

Figure S79: 500 MHz ^1^H-NMR spectrum of compound **3a** in DMSO-*d_6_*

Figure S80: 126 MHz ^13^C-NMR spectrum of compound **3a** in DMSO-*d_6_*

Figure S81: 471 MHz ^19^F-NMR spectrum of compound **3a** in DMSO-*d_6_*

### 8.3.17 DEACM-Bn-Gemcitabine, Compound 7

Figure S82: 500 MHz ^1^H-NMR spectrum of compound **7** in DMSO-*d_6_*

Figure S83: 126 MHz ^13^C-NMR spectrum of compound **7** in DMSO-*d_6_*

Figure S84: 377 MHz ^19^F-NMR spectrum of compound **7** in DMSO-*d_6_*

### 8.3.18 DEACM-Cytarabine, Compound 3b

Figure S85: 600 MHz ^1^H-NMR spectrum of compound **3b** in DMSO-*d_6_*

Figure S86: 151 MHz ^13^C-NMR spectrum of compound **3b** in DMSO-*d_6_*

### 8.3.19 TBDMS-Clofarabine, Compound 4a.1

Figure S87: 500 MHz ^1^H-NMR spectrum of compound **4a.1** in DMSO-*d_6_*

Figure S88: 126 MHz ^13^C-NMR spectrum of compound **4a.1** in DMSO-*d_6_*

Figure S89: 471 MHz ^19^F-NMR spectrum of compound **4a.1** in DMSO-*d_6_*

### 8.3.20 TBDMS-Cladribine, Compound 4b.1

Figure S90: 600 MHz ^1^H-NMR spectrum of compound **4b.1** in DMSO-*d_6_*

Figure S91: 151 MHz ^13^C-NMR spectrum of compound **4b.1** in DMSO-*d_6_*

### 8.3.21 TBDMS-DEACM-Clofarabine, Compound 4a.2

Figure S92: 500 MHz ^1^H-NMR spectrum of compound **4a.2** in DMSO-*d_6_*

Figure S93: 126 MHz ^13^C-NMR spectrum of compound **4a.2** in DMSO-*d_6_*

Figure S94: 471 MHz ^19^F-NMR spectrum of compound **4a.2** in DMSO-*d_6_*

### 8.3.22 TBDMS DEACM-Cladribine, Compound 4b.2

Figure S95: 400 MHz ^1^H-NMR spectrum of compound **4b.2** in DMSO-*d_6_*

Figure S96: 400 MHz ^1^H-NMR spectrum of compound **4b.2** in Chloroform-*d*

Figure S97: 101 MHz ^13^C-NMR spectrum of compound **4b.2** in Chloroform-*d*

### 8.3.23 DEACM-Clofarabine, Compound 4a

Figure S98: 500 MHz ^1^H-NMR spectrum of compound **4a** in DMSO-*d_6_*

Figure S99: 101 MHz ^13^C-NMR spectrum of compound **4a** in DMSO-*d_6_*

Figure S 100: 471 MHz ^19^F-NMR spectrum of compound **4a** in DMSO-*d_6_*

### 8.3.24 DEACM-Cladribine, Compound 4b

Figure S101: 400 MHz ^1^H-NMR spectrum of compound **4b** in DMSO-*d_6_*

Figure S102: 101 MHz ^13^C-NMR spectrum of compound **4b** in DMSO-*d_6_*

### 8.3.25 TBDMS-Fludarabine, Compound 5b.1

Figure S103: 500 MHz ^1^H-NMR spectrum of compound **5b.1** in DMSO-*d_6_*

Figure S104: 126 MHz ^13^C-NMR spectrum of compound **5b.1** in DMSO-*d_6_*

Figure S105: 471 MHz ^19^F-NMR spectrum of compound **5b.1** in DMSO-*d_6_*

### 8.3.26 TBDMS-CDI-Fludarabine, Compound 5b.2

Figure S106: 500 MHz ^1^H-NMR spectrum of compound **5b.2** in DMSO-*d_6_*

Figure S107: 126 MHz ^13^C-NMR spectrum of compound **5b.2** in DMSO-*d_6_*

Figure S108: 471 MHz ^19^F-NMR spectrum of compound **5b.2** in DMSO-*d_6_*

### 8.3.27 TBDMS-DEACM-Fludarabine, Compound 5b.3

Figure S109: 500 MHz ^1^H-NMR spectrum of compound **5b.3** in DMSO-*d_6_*

Figure S110: 126 MHz ^13^C-NMR spectrum of compound **5b.3** in DMSO-*d_6_*

Figure S111: 471 MHz ^19^F-NMR spectrum of compound **5b.3** in DMSO-*d_6_*

### 8.3.28 **DEACM-Fludarabine,** Compound 5b

Figure S112: 500 MHz ^1^H-NMR spectrum of compound **5b** in DMSO-*d_6_*

Figure S113: 126 MHz ^13^C-NMR spectrum of compound **5b** in DMSO-*d_6_*

Figure S114: 471 MHz ^19^F-NMR spectrum of compound **5b** in DMSO-*d_6_*

### 8.3.29 DEACM‑Methotrexate, Compound 8

Figure S115: 500 MHz ^1^H-NMR spectrum of compound **8** in DMSO-*d_6_*

Figure S116: 126 MHz ^13^C-NMR spectrum of compound **8** in DMSO-*d_6_*

### 8.3.30 DEACM-Raltitrexed, Compound 9

Figure S117: 600 MHz ^1^H-NMR spectrum of compound **9** in DMSO-*d_6_*

Figure S118: 151 MHz ^13^C-NMR spectrum of compound **9** in DMSO-*d_6_*

### 8.3.31 DEACM-Pemetrexed, Compound 10

Figure S119: 500 MHz ^1^H-NMR spectrum of compound **10** in DMSO-*d_6_*

Figure S120: 126 MHz ^13^C-NMR spectrum of compound **10** in DMSO-*d_6_*

### 8.3.32 Gemcitabine-Pyridine, Compound 11a

Figure S121: 500 MHz ^1^H-NMR spectrum of compound **11a** in DMSO-*d_6_*

Figure S122: 126 MHz ^13^C-NMR spectrum of compound **11a** in DMSO-*d_6_*

Figure S123: 471 MHz ^19^F-NMR spectrum of compound **11a** in DMSO-*d_6_*

### 8.3.33 Gem-Cy-tabine, Compound 11

Figure S124: 500 MHz ^1^H-NMR spectrum of compound **11** in Methanol-*d_4_*

Figure S125: 500 MHz ^1^H-NMR spectrum of compound **11** in DMSO-*d_6_*

Figure S126: 126 MHz ^13^C-NMR spectrum of compound **11** in Methanol-*d_4_*

Figure S127: 471 MHz ^19^F-NMR spectrum of compound **11** in Methanol-*d_4_*

## 8.4 MS Spectra

### 8.4.1 DEACM-6MP, Compound 1

### 8.4.2 Tegafur-Triazole, Compound 2b.1

### 8.4.3 DEACM-tegafur, Compound 2b

### 8.4.4 DEACM-5FU, Compound 2a

### 8.4.5 TBDMS-DEACM-Floxuridine, Compound 2c.2

### 8.4.6 DEACM-Floxuridine, Compound 2c

### 8.4.7 **TBDMS-DEACM-Bn-Floxuridine, Compound 6.2**

### 8.4.8 DEACM-Bn-Floxuridine, Compound 6

### 8.4.9 5’-DEACM-Floxuridine, Compound 5a

### 8.4.10 TBDMS-Gemcitabine, Compound 3a.1

### 8.4.11 TBDMS-Decitabine, Compound 3c.1

### 8.4.12 TBDMS-DEACM-Gemcitabine, Compound 3a.2

### 8.4.13 DEACM-Bn-CDI

### 8.4.14 TBDMS DEACM-Bn-Gem

### 8.4.15 TBDMS-DEACM-Decitabine, Compound 3c.2

### 8.4.16 DEACM-Gemcitabine, Compound 3a

### 8.4.17 DEACM-Bn-Gemcitabine, Compound 7

### 8.4.18 DEACM-Cytarabine, Compound 3b

### 8.4.19 TBDMS-Clofarabine, Compound 4a.1

### 8.4.20 TBDMS-Cladribine, Compound 4b.1

### 8.4.21 TBDMS-DEACM-Clofarabine, Compound 4a.2

### 8.4.22 TBDMS-DEACM-Cladribine, Compound 4b.2

### 8.4.23 DEACM-Clofarabine, Compound 4a

### 8.4.24 DEACM-Cladribine, Compound 4b

### 8.4.25 TBDMS-Fludarabine, Compound 5b.1

### 8.4.26 TBDMS-CDI-Fludarabine, Compound 5b.2

### 8.4.27 TBDMS-DEACM-Fludarabine, Compound 5b.3

### 8.4.28 DEACM-Fludarabine, Compound 5b

### 8.4.29 DEACM-Methotrexate, Compound 8

### 8.4.30 DEACM-Raltitrexed, Compound 9

### 8.4.31 DEACM-Pemetrexed, Compound 10

### 8.4.32 Gemcitabine-Pyridine, Compound 11a

### 8.4.33 Gemcitabine-zincke salt, Compound 11b

### 8.4.34 Gem-Cy-tabine, Compound 11

# 9. References

[1]	O. Werz, D. Steinhilber, *Eur. J. Biochem.* **1996**, *242*, 90–97, DOI /10.1111/j.1432-1033.1996.0090r.x.

[2]	S. Tang, J. Cannon, K. Yang, M. F. Krummel, J. R. Baker, S. K. Choi, *J. Org. Chem.* **2020**, *85*, 2945–2955, DOI 10.1021/acs.joc.9b02617.

[3]	H. P. Nguyen, S. Stewart, M. N. Kukwikila, S. F. Jones, D. Offenbartl-Stiegert, S. Mao, S. Balasubramanian, S. Beck, S. Howorka, *Angew. Chem. Int. Ed.* **2019**, *58*, 6620–6624, DOI /10.1002/anie.201901139.

[4]	M. Reinfelds, J. von Cosel, K. Falahati, C. Hamerla, T. Slanina, I. Burghardt, A. Heckel, *Chem. - Eur. J.* **2018**, *24*, 13026–13035, DOI 10.1002/chem.201802390.

[5]	M. Russo, H. Janeková, D. Meier, M. Generali, P. Štacko, *J. Am. Chem. Soc.* **2024**, *146*, 8417–8424, DOI 10.1021/jacs.3c14197.

[6]	X.-J. Tang, Y. Wu, R. Zhao, X. Kou, Z. Dong, W. Zhou, Z. Zhang, W. Tan, X. Fang, *Angew. Chem. Int. Ed.* **2020**, *59*, 18386–18389, DOI 10.1002/anie.202005310.

[7]	R. Klimek, M. Asido, V. Hermanns, S. Junek, J. Wachtveitl, A. Heckel, *Chem. - Eur. J.* **2022**, *28*, e202200647, DOI https://doi.org/10.1002/chem.202200647.

[8]	T. Weinrich, M. Gränz, C. Grünewald, T. F. Prisner, M. W. Göbel, *Eur. J. Org. Chem.* **2017**, *2017*, 491–496, DOI 10.1002/ejoc.201601174.

[9]	W. E. Bauta, B. E. Schulmeier, B. Burke, J. F. Puente, W. R. Cantrell, D. Lovett, J. Goebel, B. Anderson, D. Ionescu, R. Guo, *Org. Process Res. Dev.* **2004**, *8*, 889–896, DOI 10.1021/op049884n.

[10]	Y. Cen, A. A. Sauve, *Nucleosides, Nucleotides Nucleic Acids* **2010**, *29*, 113–122, DOI 10.1080/15257771003597758.

[11]	X.-H. Xu, G.-M. Yao, Y.-M. Li, J.-H. Lu, C.-J. Lin, X. Wang, C.-H. Kong, *J. Nat. Prod.* **2003**, *66*, 285–288, DOI 10.1021/np020034f.

[12]	Z. Pan, Z. Fan, B. Lu, J. Cheng, *Adv. Synth. Catal.* **2018**, *360*, 1761–1767, DOI https://doi.org/10.1002/adsc.201800006.

[13]	K. Brown, A. Weymouth-Wilson, B. Linclau, *Carbohydr. Res.* **2015**, *406*, 71–75, DOI 10.1016/j.carres.2015.01.001.

[14]	L. W. Hertel, J. S. Kroin, J. W. Misner, J. M. Tustin, *J. Org. Chem.* **1988**, *53*, 2406–2409, DOI 10.1021/jo00246a002.

[15]	I. Tsybulskaya, T. Kulak, E. Kalinichenko, A. Baranovsky, S. Bogushevich, M. Golubeva, B. Kuzmitsky, *Bioorg. Med. Chem.* **2015**, *23*, 3287–3296, DOI 10.1016/j.bmc.2015.04.059.

[16]	I. D. Konstantinova, K. V. Antonov, I. V. Fateev, A. I. Miroshnikov, V. A. Stepchenko, A. V. Baranovsky, I. A. Mikhailopulo, *Synthesis* **2011**, 1555–1560, DOI 10.1055/s-0030-1260010.

[17]	G. N. Sansom, N. S. Kirk, C. P. Guise, R. F. Anderson, J. B. Smaill, A. V. Patterson, M. J. Kelso, *Bioorg. Med. Chem. Lett.* **2019**, *29*, 1215–1219, DOI 10.1016/j.bmcl.2019.03.015.

[18]	K. Morihiro, T. Ishinabe, M. Takatsu, H. Osumi, T. Osawa, A. Okamoto, *J. Am. Chem. Soc.* **2021**, *143*, 3340–3347, DOI 10.1021/jacs.0c10732.

[19]	L. Neuville, A. Bigot, M. E. Tran Huu Dau, J. Zhu, *J. Org. Chem.* **1999**, *64*, 7638–7642, DOI 10.1021/jo990559l.
